# Comparative proteomics analysis of Tibetan hull-less barley under osmotic stress via data-independent acquisition mass spectrometry

**DOI:** 10.1093/gigascience/giaa019

**Published:** 2020-03-03

**Authors:** Yulin Wang, Zha Sang, Shaohang Xu, Qijun Xu, Xingquan Zeng, Dunzhu Jabu, Hongjun Yuan

**Affiliations:** 1 State Key Laboratory of Hulless Barley and Yak Germplasm Resources and Genetic Improvement, No.130 Jinzhu West Road, Chengguan District, Lhasa 850002, Tibet, China; 2 Institute of Agricultural Research, Tibet Academy of Agricultural and Animal Husbandry Sciences, No.130 Jinzhu West Road, Chengguan District, Lhasa 850002, Tibet, China; 3 Deepxomics Co., Ltd, No.2082 Shenyan Road, Yantian District., Shenzhen 518000, Guangdong, China

**Keywords:** Tibetan hull-less barley, osmotic stress, proteomics, DIA, quantification, abiotic stress

## Abstract

**Background:**

Tibetan hull-less barley (*Hordeum vulgare* L. var. *nudum*) is one of the primary crops cultivated in the mountains of Tibet and encounters low temperature, high salinity, and drought. Specifically, drought is one of the major abiotic stresses that affect and limit Tibetan barley growth. Osmotic stress is often simultaneously accompanied by drought conditions. Thus, to improve crop yield, it is critical to explore the molecular mechanism governing the responses of hull-less barley to osmotic/drought stress conditions.

**Findings:**

In this study, we used quantitative proteomics by data-independent acquisition mass spectrometry to investigate protein abundance changes in tolerant (XL) and sensitive (DQ) cultivars. A total of 6,921 proteins were identified and quantified in all samples. Two distinct strategies based on pairwise and time-course comparisons were utilized in the comprehensive analysis of differentially abundant proteins. Further functional analysis of differentially abundant proteins revealed that some hormone metabolism–associated and phytohormone abscisic acid–induced genes are primarily affected by osmotic stress. Enhanced regulation of reactive oxygen species (may promote the tolerance of hull-less barley under osmotic stress. Moreover, we found that some regulators, such as GRF, PR10, MAPK, and AMPK, were centrally positioned in the gene regulatory network, suggesting that they may have a dominant role in the osmotic stress response of Tibetan barley.

**Conclusions:**

Our findings highlight a subset of proteins and processes that are involved in the alleviation of osmotic stress. In addition, this study provides a large-scale and multidimensional proteomic data resource for the further investigation and improvement of osmotic/drought stress tolerance in hull-less barley or other plant species.

## Background

Plant growth is often affected by several environmental abiotic or biotic stresses, which induce various biochemical and physiological responses in plants [1]. Among the abiotic stresses, drought is one of the most prevalent and complex environmental threats presently affecting agriculture [2]. The drought-afflicted agricultural areas are estimated to double by the end of the 21st century [3]. Severe drought can result in a significant reduction in crop yields due to adverse impacts on plant growth and development [4]. Lesk et al. used a statistical method to examine disasters from 1964 to 2007 and reported that drought and extreme heat environmental conditions would significantly reduce national cereal production by 9–10% [5]. Therefore, it is important to develop drought-tolerant and well-adapted cultivars under water deficit conditions to improve crop yield [3].

To cope with drought stress, plants have developed a variety of mechanisms to confront threats from adverse environmental factors. The adaptive responses of these plants are dynamic and contain both reversible and irreversible changes, including alterations of membranes, changes in cell wall architecture, and adjustments in mitosis [6–8]. In addition, drought can trigger a variety of physiological or biological responses for their adaptation to arid environments. These responses include stomatal closure, inhibition of cell growth, regulation of photosynthesis, and adjustment of respiration [9]. Plants have also evolved various mechanisms to overcome water-limited conditions at both the cellular and molecular levels, such as the accumulation of osmolytes or antioxidants [10]. In addition, a previous review reported that the phytohormone abscisic acid (ABA) core signalling pathway could mediate several rapid responses to improve tolerance in drought conditions, including gene regulation, stomatal closure, and plant growth modulation [11]. To date, many genes have been recognized and shown to function in stress conditions. These genes consist of transcription factors (AREB, NAC, bZIP, MYC, and MYB) and signalling protein kinases (mitogen-activated protein kinase [MAPK], receptor protein kinase, transcription regulation protein kinase, calcium-dependent protein kinase, and ribosomal protein kinase) [1, 12–16].

Tibetan hull-less barley (*Hordeum vulgare* L. var. *nudum*, NCBI:txid4513), also named “Qingke” in Chinese, is a major cereal grain grown on the Tibetan Plateau. Zeng et al. completed the draft genome of Tibetan hull-less barley and made a number of findings regarding its adaptation to harsh environments on the Tibetan highlands [17]. Next, 2 transcriptome datasets were generated to explore the expression changes in nitrogen deprivation [18] and drought stress [19]. To the best of our knowledge, no large-scale proteomic research of Tibetan hull-less barley has been performed under drought stress. Indeed, messenger RNA (mRNA) expression is not always a good predictor of protein abundance because low correlations between mRNA and protein abundance are often observed [20–22]. Thus, precise measurement of the proteome is meaningful for understanding the underlying biological mechanisms of Tibetan hull-less barley under osmotic/drought stress.

In recent years, data-independent acquisition mass spectrometry (DIA-MS) has emerged as an important technique in quantitative proteomics [23, 24]. Compared with shotgun proteomics in data-dependent acquisition (DDA) mode, data-independent acquisition (DIA) could offer a potentially deeper coverage of the data in shorter analysis times. The data obtained through this method show fewer missing values, higher precision, and better reproducibility across replicates [25]. In this study, we used polyethylene glycol (PEG)-induced osmotic stress to simulate drought conditions. Next, we used the DIA-MS method to perform a comprehensive proteomic profiling of Tibetan hull-less barley under osmotic stress. Time-course and pairwise comparison analyses of all samples at each time point were conducted with the protein abundance. Then, we examined the physiological or biological processes of differentially abundant proteins (DAPs) in each comparison. Our analysis revealed several important stress-responsive genes and functional modules, such as hormone metabolism, including ethylene, salicylic acid (SA), and cytokinin; cell wall or cell architecture associated with membrane stability; reactive oxygen species (ROS)–scavenging enzymes; and ABA-induced signalling genes. We further selected some known drought stress–responsive genes from public databases or articles and explored their distribution curves at each time point. Finally, using a machine-learning approach, we constructed a gene regulatory network and revealed several key regulatory elements associated with osmotic stress tolerance.

## Data Description

### Plant materials and cultivation

Two Tibetan hull-less barley inbred lines, drought-sensitive (DQ) and drought-resistant (XL), were used for our experiments. Specifically, we acquired the DQ cultivar from the Institute of Agricultural Research, Tibet Academy of Agricultural and Animal Husbandry Sciences, Lhasa, Tibet, China, and the XL cultivar from the Tibet Autonomous Region Xigaze Agricultural Science Research Institute, Xigaze, Tibet, China. Seeds of the 2 cultivars were sown with nutritional soil and maintained in plant growth incubators at 25°C, 2,000 µmol m^−2^ s^−1^. In the 2–3 leaf stage, seedlings were removed from the tray and cultivated in half-strength Hoagland's nutrient solution [26]. Specifically, a PEG solution with a concentration of 21% was used to simulate osmotic stress caused by drought. For each cultivar, half of the plants were replaced with PEG6000 embedding medium after 7 days of growth. Next, fresh leaves from 2 cultivars in the control group (CK) and stress treatment group (ST) were sampled at 1, 4, 8, 12, 24, and 48 h, respectively. For the individual plants with specific sampling time points and treatments, 3 replicates were collected and then kept at −80°C until they were analysed.

### Protein extraction and digestion

For each plant tissue sample, a 1-g subsample was weighed and homogenized by grinding in liquid nitrogen. The powdered samples were moved to 50 cm^3^ tubes with 25 cm^3^ precooled acetone (−20°C) containing 10% (v/v) trichloroacetic acid and 10  mM dithiothreitol. After thorough mixing, the homogenate was precipitated overnight at −20°C and then centrifuged (20,000*g*, 4°C) for 30  min. The pellet was then washed twice with 20 cm^3^ chilled acetone (−20°C) and left at −20°C for 30  min followed by centrifugation (20,000*g*, 4°C) for 30  min. The precipitate was dissolved with lysis buffer (4% sodium dodecyl sulfate, 100  mM Tris-HCl, 10 mM dithiothreitol, pH  8.0) and sonicated for 5  min at 60  W (5  s sonication followed by 10  s break) followed by 30  min centrifugation (20,000*g*, 20°C). The supernatant was collected, and the protein concentration in the lysate was estimated using a bicinchoninic acid protein assay kit (Beyotime Institute of Biotechnology, China).

Protein digestion was conducted using the FASP (filter-aided sample preparation) procedure [27]. In brief, protein extracts in an ultrafiltration filtrate tube (30  kDa cut-off, Sartorius, Germany) were mixed with 200  mm^3^ UA buffer (8  M urea, 150  mM Tris-HCl, pH  8.0) and centrifuged at 14,000*g* at 20°C for 40  min. Samples were washed twice by adding 200  mm^3^ UA to the filter unit and centrifuged at 14,000*g* at 20°C for 40  min. After discarding the flow-through from the collection tube, 100 mm^3^ IAM solution (10  mM IAM in UA buffer) was added to the filter tube and incubated for 30 min. Samples were washed twice with 100 mm^3^ of UA to the filter unit. After centrifuging with 14,000*g* at 20°C for 40  min, 100 mm^3^ of ABC (0.05 M NH_4_HCO_3_ in water) was added into the filter unit and centrifuged at 14,000*g*. The protein suspension in the filtrate tube was subjected to enzyme digestion with 40 mm^3^ ABC with trypsin (Promega, USA) and incubated for 18 h at 37°C. The filtrate was used for liquid chromatography–mass spectrometry (LC-MS) analysis after centrifugation at 14,000*g* for 10 min. The quality control (QC) mixture was formed by pooling equal amounts of peptides from all individuals, which was used to evaluate the reproducibility of the quantitative LC-MS analysis.

### Peptide fractionation by high-pH reversed-phase

Digested peptides were separated on an LC-20AB HPLC system (Shimadzu, Kyoto, Japan) with a high-pH reversed-phase column (Phenomenon, Torrance, CA, USA). Peptides were eluted at a flow rate of 0.8  cm^3^ min^−1^. Buffer A consisted of 10  mM ammonium acetate (pH  10.0), and buffer B consisted of 10  mM ammonium acetate and 90% v/v acetonitrile (pH  10.0). The following gradient was applied to perform separation: 100% buffer A for 40  min, 0–5% buffer B for 3  min, 5–35% buffer B for 30  min, 35–70% buffer B for 10  min, 70–75% buffer B for 10  min, 75–100% buffer B for 7  min, 100% buffer B for 15  min, and, finally, 100% buffer A for 15  min. The elution process was monitored by measuring absorbance at 214  nm, and fractions were collected every 75  s. Finally, collected fractions (∼40) were combined into 12 pools. Each fraction was concentrated via vacuum centrifugation and reconstituted in 40 mm^3^ of 0.1% v/v formic acid. All samples were stored at −20°C until further analysis.

### LC-MS analysis

Peptides were separated with a Dionex UltiMate 3000 RSLCnano system with an Acclaim PepMap C18 (3 μm, 100 Å, 75 μm x 50 cm) and emitted into a Thermo Q-Exactive HF tandem mass spectrometer. Solvent A was 0.1% formic acid in water, while solvent B was 0.1% formic acid in 98% acetonitrile. For each injection, 3 mm^3^ (∼3  μg) was loaded and eluted using a 90-minute gradient from 5 to 35% B followed by a 40-min washing gradient. Data were acquired using either DDA or DIA.

For library generation, the Thermo Q-Exactive HF was set to positive mode in a top-20 configuration to acquire data in DDA mode. Precursor spectra (375–1,400*m*/*z*) were collected at 120,000 resolution to reach an automatic gain control (AGC) target of 3e6. The maximum injection time was set to 20  ms. Fragment spectra were collected at 30,000 resolution to reach an AGC target of 1e5 with a maximum injection time of 60  ms. The isolation width was set to 1.6*m*/*z* with a normalized collision energy of 25. Only precursors charged between +2 and +6 that achieved a minimum AGC of 2e3 were acquired. Dynamic exclusion was set to 30 s and to exclude all isotopes in a cluster.

For quantitative samples, the Thermo Q-Exactive HF was configured to acquire 55  ×  16 *m/z* DIA spectra (16 *m/z* precursor isolation windows at 30,000 resolution, AGC target 1e6, maximum injection time 55  ms). Precursor spectra (400–1,250*m/z*) were collected at 120,000 resolution to reach an AGC target of 3e6. The maximum injection time was set to 50  ms. To evaluate the reproducibility of the LC-MS system during the whole DIA acquisition, the samples and QCs were analysed following this scenario: 1 QC injection followed by 10 experimental samples until all were measured.

### Library generation and quantitative data analyses

MaxQuant (version 1.6.2.6; RRID:SCR_014485) software [28, 29] was used to analyse the DDA MS/MS data with the following settings: enzyme: trypsin/P; maximum missed cleavages: 2; fixed modification: carbamidomethyl (C); variable modifications: oxidation (M) and acetyl (protein N-term); precursor mass tolerance: 20 ppm; fragment mass tolerance: 0.05 Da; second peptide search was enabled. All other parameters were in default. The MS/MS data were searched against the *Hordeum vulgare* (barley) protein sequences, which were downloaded from the UniProt database (version 2018.7, 210,953 entries), appended with the Biognosys indexed retention time (iRT) peptide sequences. The false discovery rate (FDR) threshold was set as 1% at both peptide spectrum match and protein levels. Subsequently, the MaxQuant search result was imported into Spectronaut Pulsar (12.0.20491.4, Biognosys, Schlieren, Switzerland) to generate a spectra library with the default settings.

Spectronaut Pulsar was used to analyse the DIA data with the spectra library based on DDA MS/MS data. Local regression normalization was used for protein quantification normalization. Dynamic MS1 and MS2 mass tolerance strategies were applied for data extraction with a correction factor of 1. A dynamic extracted ion chromatogram retention time extraction window with a local nonlinear iRT strategy was chosen for calibration. Interference correction was enabled to automatically remove fragments that interfere with other ions across several runs. The decoy method in the feature identification was configured as “mutated” with a decoy limit strategy of “dynamic” and library size fraction of 0.1. The results were filtered by 1% FDR, and only those protein groups that passed these filter criteria were used in downstream analysis. The DIA raw data and the corresponding results were deposited into the iProX database [30].

### Bioinformatic data analysis

Statistical analysis and graphical display were performed with the R language environment (version 3.5.0). Hierarchical clustering was performed using the R package pheatmap [31]. Principal component analysis (PCA) was performed using the FactoMineR package [32]. A *t*-test was used for statistical differential analysis, and a cut-off of *P*-value ≤0.05 and fold change ≥2 was used to select statistically differentially abundant proteins. Hypergeometric-based enrichment analysis with KEGG (KEGG, RRID:SCR_012773) [33], Gene Ontology [34, 35], and MapMan (MapMan, RRID:SCR_003543) [36–38] was performed to annotate protein sequences individually. The abundance curve of the target gene was depicted with protein abundance. A LOESS method implemented in the R environment was used to fit the smooth curves by a set of data points [39]. For network analysis, the target genes of plant transcription factors and protein kinases are classified by the iTAK program [40]. The Arboreto computational framework integrated with the GRNBoost2 [41] algorithm was used to reconstruct relevant regulatory relationships in each ecotype. The igraph package was used to visualize networks [42].

## Analyses

### Quality control analysis of the barley proteome

In this study, we identified a total of 6,921 proteins with a 1% FDR in all samples, with a maximum of 6,313 proteins being quantified in a single non-QC sample (i.e., the replicate No. II sample of XL treatment group at 48 h in Fig. S1). The MS platform was stable and repeatable as evaluated by QC runs during the entire data-collecting period. The coefficient of variation (CV), reflecting the magnitude of variability in protein abundance, accounted for a mean value of 20% in each sample (Fig. S2a). The relationship between CV and the log area is illustrated in Fig. S2b, and proteins were assorted into 12 intervals according to their log area values in descending order. The results revealed that proteins with higher intensity always showed smaller CVs, which is in accordance with a previous study [43]. The hierarchical clustering-based heat map and PCA based on quantified protein abundances in each sample were used for further quality control, as illustrated in Fig. S2c and d. The 9 QC samples were clustered together, which indicates that the MS platform was stable and the quality of DIA data was high.

### Pairwise differential abundance analysis

**Figure 1:**
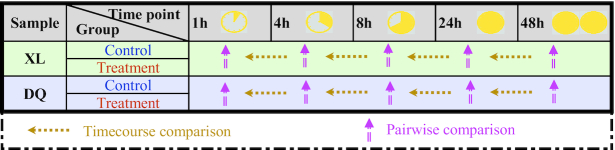
Comparison of differentially abundant proteins. The pink arrows indicate the comparison between the treatment and control groups. The yellow arrows indicate the comparison in the consecutive developmental stages over time points.

**Figure 2:**
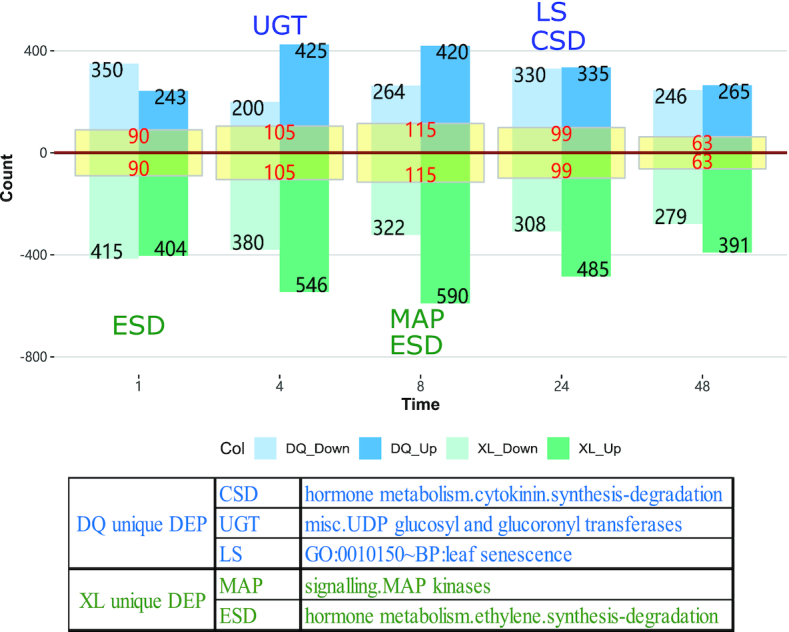
Downregulated and upregulated proteins in the DQ and XL cultivars between the treatment and control groups. The graph is based on the differential abundance analysis by pairwise comparison, showing the number of proteins that are significantly differentially expressed. The blue bars represent the DQ samples, and the green bars represent the XL samples. Among them, the dark and light colours denote the upregulated and downregulated proteins, respectively. The yellow area in the centre of the bar provides the intersection number of DAPs between XL and DQ. The abbreviations beside the bars are the unique annotated functional entries from XL or DQ, and they are manually selected according to the correlation with the osmotic stress resistance.

To explore proteins associated with osmotic stress, 2 types of analysis were performed. According to the time point experimental design of XL and DQ, all cultivars were divided into 10 comparison groups (Fig. 1). Each group consisted of a treatment-control pair, and the relative fold change of protein was calculated for each paired group. The statistical significance of the observed fold change was determined by paired *t*-test for all the DAPs, and the threshold of *P*-value ≤0.05 and fold change ≥2 was used. As shown in Fig. 2, the DAP numbers varied significantly at different time points together with a relatively low number of common changes (yellow area), indicating highly diverse dynamics of protein expression regulation in XL and DQ. Compared with downregulated proteins, more upregulated proteins were found at 4 and 8 h.

To explore the biological processes in each DAP group, we conducted hypergeometric-based enrichment analysis based on MapMan and Gene Ontology (GO) databases. The threshold of an adjusted *P*-value ≤0.05 was used to define significantly enriched annotation categories. To highlight the key function terms, we manually reviewed the biological function terms in Supplementary Tables S1–S3. Unique osmotic-related entries of DL or XL were selected and labelled beside the related bars with short abbreviations. In particular, “cytokinin synthesis degradation,” “UDP glucosyl and glucoronyl transferases (UGTs),” and “leaf senescence” were the dominant responses in DAPs of the DQ cultivar, whereas “MAP kinases” and “ethylene synthesis degradation” were 2 key terms in DAPs of the XL cultivar. More details can be obtained through the table below the diagram in Fig. 2.

**Figure 3:**
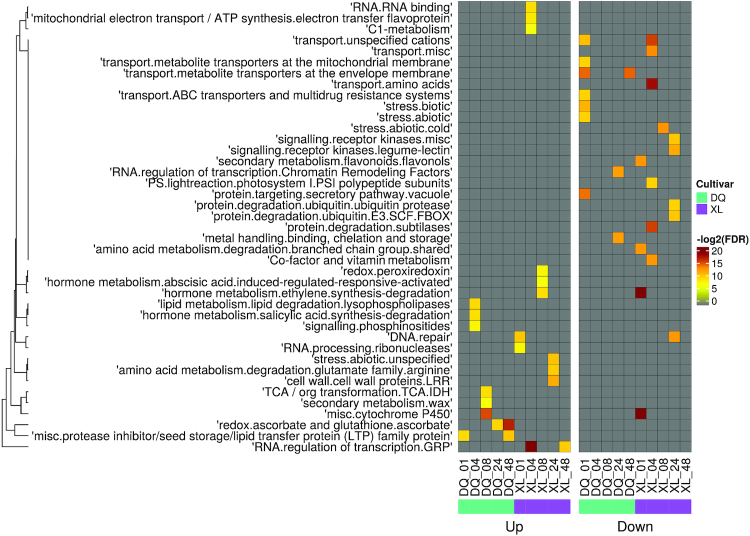
MapMan enriched heat map for DAPs in pairwise comparison. The left map shows the annotation of upregulated proteins, and the right map shows the annotation of downregulated proteins. Row names represent the samples from 5 time points in the DQ and XL cultivars. Column names are the enriched functional categories in the Mapman database. The legend shows the colour scaling with FDR values. Specifically, the coloured cells are the significantly enriched terms with FDR ≤ 0.01, and the grey cells are not.

To further explore the biological functions of DAPs, we divided the upregulated and downregulated genes into independent gene sets and reannotated them separately. Based on the Mapman annotations, we used a hierarchical heat map to represent the relationship between time stages and relevant annotated entries. As shown in Fig. 3, the colour scale was graded to reflect the enrichment scores (log_2_-transformed FDR). Among these functional terms, 2 hormone metabolism terms, “abscisic acid induced-regulated-responsive-activate” and “ethylene synthesis-degradation,” were significantly enriched in the XL upregulated gene set at 8 h. Another hormone metabolism term, “salicylic acid synthesis-degradation,” was upregulated in the sensitive cultivar (DQ) at 4 h. Moreover, some proteins involved in cell wall formation were upregulated at 24 h in the XL cultivar, and some proteins involved in wax biosynthesis were also upregulated at 8 h in the DQ cultivar. Interestingly, cytochrome P450, an important protein-coding gene family involved in growth and drought stress responses [44], was upregulated in DQ but downregulated in the XL cultivar. A similar result from Wendelboe-Nelson and Morris showed that cytochrome P450 was downregulated in tolerant cultivars [45].

We then conducted gene ontology (GO) enrichment analysis. The assigned functions of these genes covered a broad range of GO categories (Fig. S3). Specifically, in the biological process category, sulfate assimilation, cellular response to oxidative stress, and chitin catabolic process were the major functional terms for the osmotic stress response in the DQ cultivar. In contrast, the ethylene biosynthetic process, toxin catabolic process, and photosynthesis of light harvesting in photosystem I may be involved in osmotic stress tolerance in the XL cultivar. For the genes enriched in categories related to cellular components, several photosynthesis terms of photosystem I and photosystem II were upregulated in several stages of XL. For the molecular function category, many potential osmotic stress–induced genes were classified into a series of redox-related functional items, including glutathione peroxidase activity, glutathione transferase activity, peroxiredoxin activity, pigment binding, and chlorophyll binding. Additionally, we identified some chitin-binding proteins related to the pathogenesis-related gene family, which may contribute to the defence response of plants under osmotic stress [46]. Finally, based on the MapMan and GO annotations of pairwise analysis, we generated Supplementary Tables S7 and S8 to summarize the differences in response to osmotic stress between DQ and XL.

### Time-course differential abundance analysis

**Figure 4:**
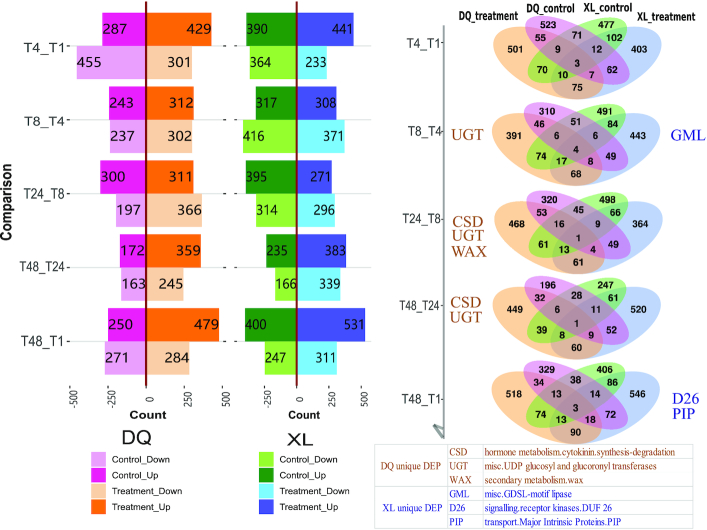
Downregulated and upregulated proteins in the DQ and XL cultivars compared over 5 time points. The pink and orange bars correspond to the control and treatment groups of the DQ samples, and the green and blue bars correspond to the control and treatment groups of the DQ XL samples. Of these, the dark and light colours denote upregulated and downregulated proteins, respectively. The Venn diagrams show the overlap of 4 groups in each comparison. In particular, each group contained both upregulated and downregulated DAPs. The abbreviations beside the circles provide the unique annotated functional entries of XL treatment or DQ treatment, and they are manually selected according to the correlation with the osmotic stress resistance.

To investigate the impact of stress degree differences on protein abundance in the consecutive developmental stages over time, stepwise comparisons (e.g., T4 vs T1, T8 vs T4, T24 vs T8, T48 vs T24, and T48 vs T1) were performed in the treatment and control groups of DQ and XL samples separately (Figs 1 and 4). DAPs were selected on the basis of the threshold of protein abundance fold changes ≥2 and *P*-value ≤0.05. To explore the relationship of significant DAPs in different treatments of XL and DQ, we generated 5 Venn diagrams. Next, we carried out the functional characterization of unique DAPs in the DQ and XL treatments individually. Several potential osmotic stress–induced entries unique to DQ or XL treatments are manually selected and labelled beside the diagram with abbreviations. The complete annotation list can be obtained in Supplementary Tables S4–S6. For instance, “cytokinin synthesis degradation,” “UDP glucosyl and glucoronyl transferases,” and “wax-related” were likely to be an exclusive response in the DQ cultivar, whereas “GDSL-motif lipase,” “DUF26 kinase,” and “plasma membrane intrinsic protein (PIP)” were 3 main functional terms in the XL cultivar.

### Core genes in the plant defence response

**Figure 5:**
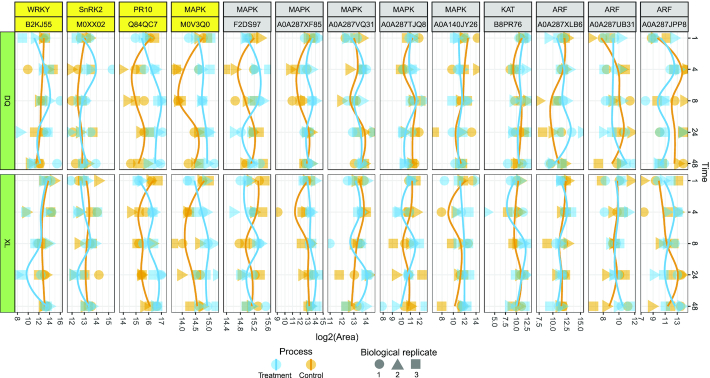
Protein abundance changes of 6 core genes in the plant defence response. The labels in the first row of the top panel are the target gene names, and the labels in the second row are the relevant UniProt accession of *Hordeum vulgare* based on BLAST alignment. The biological replicates from the same sample are represented by 3 different symbols. The treatment and control groups are illustrated with blue and orange colours, respectively.

To discover potential osmotic stress–induced genes, we explored the abundances of some well-known genes related to the plant defence response, such as ARF, KAT, MAPK, PR10, SnRK2, and WRKY. With BLAST alignment, we obtained the UniProt accession that is relevant to the candidate genes. Next, we examined the individual abundance levels of these genes and depicted the abundance profiles in Fig. 5. Through closer examination of these genes, we found that MAPK (M0V3Q0) and PR10 (Q84QC7) exhibited higher abundance in the treatment group over all time points of XL and DQ, indicating that the 2 genes might play important roles in plant defence against osmotic stress. Additionally, SnRK2 (M0XX02) and WRKY (B2KJ55) also showed a similar trend, which were upregulated at 4 and 8 h of DQ but downregulated at 24 h of XL.

To further investigate the potential osmotic stress tolerance mechanisms, we collected manually curated genes involved in the drought stress response from a public database: DroughtDB [47]. We selected the best hit for each subject sequence with the threshold of e-value ≥0.00001 and identity ≥80% using BLAST. Most of the genes were aligned to the genes identified in this study. As shown in Fig. S4, none of these genes showed significant abundance changes between the DQ and XL cultivars. In addition, we selected 4 water deprivation–related GO terms, including cellular response to water deprivation, response to water deprivation, response to desiccation, and positive regulation of response to water deprivation, and we collected the relevant protein sequences from *Oryza sativa* (rice), as annotated by UniProt. We performed a similar analysis, and as shown in Fig. S5, compared with the control group, most of the genes in the treatment group showed relatively higher abundance levels in both the DQ and XL cultivars. This phenomenon indicates that water deprivation is a vital regulatory mechanism for both XL and DQ under osmotic stress.

### Gene regulated network

**Figure 6:**
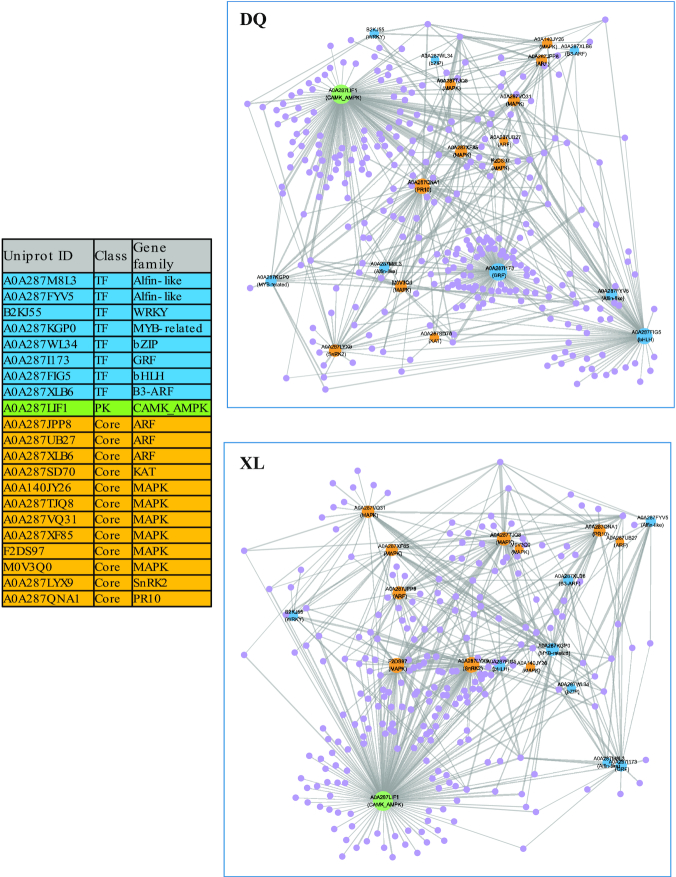
Regulated network analysis of osmotic stress–induced genes. The table on the left presents the target gene list used in this analysis. Blue and green nodes in the network correspond to the transcription factors and protein kinases, respectively. The orange nodes represent the manually reviewed core genes that are described in Fig. 5. The purple nodes represent the identified genes in the XL or DQ cultivars. The size of a node is proportional to its degree. Nodes with higher degrees, which means having more neighbours, will have a stronger capacity to modulate adjacent genes than genes with lower degrees.

Considering that genes could produce complex dynamic systems or gene regulatory networks to defend against osmotic stress during plant growth, we explored the co-expression patterns and potential regulatory associations that were represented in gene regulatory networks (GRNs). Specifically, 21 potential stress-responding genes were chosen as the candidate target gene set (Fig. 6). Among these genes, 8 transcription factors are in the families of Alfin-like, WRKY, MYB-related, bZIP, GRF, bHLH, and B3-ARF; 12 genes belong to important osmotic stress–responsive genes, including ARF, MAPK, SnRK2, and PR10; and 1 gene encodes AMP-activated protein kinase (AMPK). Then, Arboreto takes this target-gene abundance matrix as inputs and produces reliable interaction predictions. On the basis of the abundances of a set of candidate genes, we constructed a partial GRN with regulatory associations using the identified stress-responding genes for each ecotype (see Methods). As shown in Fig. 6, this result revealed that bHLH, GRF, and PR10 had more connections in DQ than in XL, indicating that these 2 genes probably play important roles in the osmotic stress response process of the DQ cultivar. In addition, MAPK and SnRK2 showed more connections in XL than in DL. Remarkably, AMPK was the hub gene with the highest connection number in both the XL and DQ cultivars.

## Discussion

Drought is one of the most acute environmental stresses that directly affects agricultural productivity. In this study, we first used DIA-MS–based proteomics technology to quantify proteins in different samples and explored essential DAPs in hull-less barley over multiple time points under 2 cultivars. Using 2 different comparison strategies, time-course and pairwise, we conducted a comprehensive analysis to explore protein-level changes in response to osmotic stresses.

We detected some essential biological function terms related to osmotic stress regulation in the DQ cultivar. Specifically, cytokinins are a class of growth-promoting hormones regulating various developmental processes, including cell division and senescence [48]. Previous studies revealed that reduced cytokinin levels could improve osmotic stress tolerance by suppressing growth and reducing stomatal density [49, 50]. Additionally, the “leaf senescence” entry at 24 h presents an accelerated leaf senescence of the DQ cultivar and implies that the DQ cultivar might be more sensitive to osmotic stress. Moreover, UDP-glucuronosyl/UDP-glucosyl transferases, with pronounced changes at 4 h of DQ, are a superfamily of enzymes that catalyze glucuronidation reactions [51], and they were found to enhance plant tolerance under a series of adverse environmental factors, including low temperatures, salinity, and drought [52]. Furthermore, we found that the function term “salicylic acid synthesis-degradation” was only upregulated in the sensitive cultivar (DQ). SA is a vital phytohormone required for systemic acquired resistance in plants and plays a vital role in the defence against pathogens [53]. It has also been reported that SA could ameliorate oxidative stress and enhance plant tolerance to abiotic stress [54]. Our results postulate that the degradation of SA tends to make the DQ cultivar more susceptible under osmotic stress.

The functional terms from ABA-dependent and ABA-independent signalling pathways exhibited dominant abundance changes in the XL cultivar. Several studies have proven that ABA plays a key role in regulating the adaptive response of plants under diverse stress conditions [55, 56]. Meanwhile, the plant hormone ethylene is well known to play an essential role in plant growth, development, and osmotic stress resistance. In particular, lower ethylene levels would lead to higher osmotic stress tolerance. Shi et al. also indicated that a reduced sensitivity to ethylene by CRISPR-Cas9 technology would enhance cell elongation and division, thereby increasing grain yield under osmotic stress conditions [57]. Moreover, glycine-rich RNA-binding proteins (GRPs) are known to transport and regulate RNA processing. A study by Kim et al. suggested that GRPs influence the opening and closing of the stomata [58]. Vítámvás et al. reported that drought treatments profoundly affected glycine-rich RNA-binding protein abundances [59]. In the present study, glycine-rich RNA-binding protein abundances were found to increase only in the XL cultivar, indicating that these proteins may improve osmotic stress tolerance in hull-less barley.

In the biological process shown in Fig. S3, some upregulated proteins of the XL cultivar were enriched in toxin catabolic processes from 4 to 8 h. This catabolic process may detoxify the ROS produced during osmotic stress treatments, and it is one of the potential mechanisms that makes XL more tolerant to environmental stress than DQ. Moreover, flavonoids are thought to be one of the key compounds that protect plants against various biotic and abiotic stresses by inhibiting ROS formation [60]. However, the enzymes involved in flavonoid metabolism were downregulated in the early stage of the XL cultivar. Interestingly, a similar phenomenon can be observed in the report by Vítámvás et al. [59].

Ascorbate peroxidase (APX) and glutathione peroxidase (GPX) are 2 plant antioxidant enzymes that can remove H_2_O_2_ and prevent potential cellular damage [61, 62]. Interestingly, ascorbate peroxidase activity was found to increase from 24 to 48 h of DQ and 48 h of XL (Fig. 3S). The increased protein abundance of APX was also observed in both tested barley lines by Chmielewska et al. [63]. The findings of these researchers are in accordance with our detections, and we postulated that DQ, with a quicker response, is more susceptible to osmotic stress than the XL cultivar. In addition, Vítámvás et al. revealed that GPX had a continuous and significant increase with decreasing soil water capacity [59]. A similar phenomenon was observed in our results with increasing glutathione peroxidase activity at 4 h in the XL cultivar, indicating that GPX has the capacity to enhance tolerance against abiotic stress. Moreover, peroxiredoxins (Prxs) are a highly conserved family of antioxidant enzymes that catalyze the peroxide reduction of H_2_O_2_. Ghabooli et al. showed that the protein levels of APXs and Prxs were upregulated in barley plants under drought treatment [64]. Similarly, we found that peroxiredoxin activity was significantly increased at 8 h in the XL cultivar (Fig. 3 or Fig. S3). Furthermore, glutathione transferase, which is believed to conjugate xenobiotics with glutathione [59], was also upregulated at 8 h of XL. These results may reveal that the XL cultivar has more ROS scavenging mechanisms to enhance osmotic stress tolerance than the DQ cultivar.

We also identified several genes relevant to cell wall construction. Because the cell wall is the first line to defend against abiotic stress, many proteins that are involved in cell wall strengthening or cellular membrane stabilization will be significantly regulated under osmotic stress [65]. Interestingly, a variety of transport-related proteins were downregulated under osmotic stress in the first time point of the DQ cultivar (Fig. S3), indicating that DQ is more sensitive than the XL cultivar and promotes osmotic tolerance through suppression of several transport activities in the early stage of development.

In the time-course comparison, we identified genes related to cytokinin degradation and UGT in the DQ cultivar treatment group. Moreover, we found some DAPs from the DQ cultivar that can produce a secondary metabolite (wax) in time-course comparison analysis, as shown in Fig. 4. The increased accumulation of cuticular wax under osmotic stress conditions can improve tolerance and reduce water loss [66]. In addition, 3 manually reviewed entries were specific in the treatment group of the XL cultivar. Of these entries, Hong et al. demonstrated that GDSL-type lipase can activate susceptibility to disease and tolerance to abiotic stress [67]. Miyakawa et al. demonstrated that a plant-specific cysteine-rich motif (DUF26) may be widely involved in plant-specific responses to biotic and abiotic stresses [68]. Lu et al. showed that changes in the gene expression of some plasma membrane intrinsic proteins (PIPs) can also promote osmotic stress tolerance [69]. Overall, the analyses carried out in this study have confirmed findings reported in previous studies and provided additional evidence of abiotic tolerance in resistant compared to susceptible cultivars.

To investigate the expression status of several well-known osmotic stress genes, we found that 4 proteins (MAPK, PR10, SnRK2, and WRKY) had significant changes in protein abundance between the control and treatment groups. Of these, the MAPK cascade is one of the major signalling pathways involved in the abiotic stress response in plants [70]. It is evolutionarily conserved among eukaryotic organisms and can transduce extracellular signals to the nucleus under abiotic stress [71, 72]. The PR10 gene has been confirmed to be overexpressed in rice and to enhance drought and salt stress tolerance [73]. Additionally, WRKY transcription factors were thought to participate in the regulation of water stress and drought responses [74]. Taken together, these results demonstrated that these genes were potential candidate genes for agricultural application to protect crops against biotic and abiotic stresses.

In the gene regulatory network, we found a few genes centrally positioned in the network, suggesting that these genes may have a dominant role/regulation in Tibetan hull-less barley. Specifically, growth regulating factors (GRFs) and the basic helix-loop-helix (bHLH) protein family are plant-specific transcription factors that are involved in diverse biological or physiological processes, such as growth, hormone responses, and stress [75, 76]. AMPK, known to be responsible for the maintenance of ATP balance during energy metabolism [77], occupies the central position in both networks, indicating that it is likely to be a core regulatory component in the osmotic stress resistance network. Moreover, SnRK2, a serine/threonine kinase specific in plants involved in plant responses to abiotic stresses and ABA-dependent plant development [78], showed higher abundance in XL than in DQ. Furthermore, we also found that MAPK-related genes showed more connections in the XL cultivar. Previous studies suggested that MAPK could be activated by the ABA core signalling module through transcriptional regulation [11]. Thus, we could infer that the ABA-induced pathway might have a stronger impact on XL than the DQ cultivar.

## Conclusions

This proteomic study provides a valuable resource to explore stress-responsive proteins that can help us understand the underlying regulatory mechanisms in Tibetan hull-less barley. Furthermore, these data will be valuable to plant biologists who are interested in exploring signalling mechanisms to osmotic/drought stress, thereby helping to promote drought stress tolerance in crops.

## Availability of Supporting Data and Materials

All of the MS raw data (DIA and DDA) have been deposited to the ProteomeXchange Consortium via the iProX partner repository [30] with the dataset identifier PXD015597. All supporting data and materials are available in the *GigaScience* GigaDB database [79].

## Additional Files

**Supplementary Figure S1**. Numbers of proteins detected in each sample.

**Supplementary Figure S2a**. Distribution of protein abundance variability. The CV value of each protein was calculated by R environment with formula as “sd(biological replicates)/mean(biological replicates).”

**Supplementary Figure S2b**. Relationship between CV and protein abundance (log_2_ transformed). The CV value decreases with increasing protein abundance.

**Supplementary Figure S2c**. Heat map of protein abundances between different samples. The hierarchical clustering is performed using a neighbor-joining algorithm with a Euclidean distance similarity measurement of the log_2_ of the protein abundance.

**Supplementary Figure S2d**. Principal component analysis (PCA) score plot for proteins in the DQ and XL cultivars between the treatment and control groups. Each point represents a sample.

**Supplementary Figure S3**. Gene ontology enriched heat map for DAPs in pairwise comparison. Similar to Fig. 3, but with gene ontology instead of MapMan database. The left panel shows the annotation of upregulated proteins, and the right panel shows the annotation of downregulated proteins. Row names are the samples from 5 time points in the DQ and XL cultivars. Column names are the enriched items from 3 aspects of gene ontology database (biological process: BP; cellular component: CC; and molecular function: MF). The legend shows the colour scaling with FDR values. See Supplementary Table S1 for the entire list of GO terms.

**Supplementary Figure S4**. Protein abundance changes of osmotic stress–induced genes from the DroughtDB database. Similar to Fig. 5, the labels in the left panel are the description of the related genes. In particular, the identifier to the left of the tilde (∼) symbol is the UniProt accession of *Hordeum vulgare*, the identifier to the right of the tilde is the gene symbol from DroughtDB, and the description under the tilde is the osmotic stress–related functional annotation.

**Supplementary Figure S5**. Protein abundance changes of osmotic stress–induced genes from Gene Ontology database. Similar to Fig. 5, the labels in the first left panel are the UniProt accessions of *Hordeum vulgare*, and the labels in the second left panel are the functional description from gene ontology with BLAST.

**Supplementary Table S1**. Gene ontology enrichment list of DAPs in pairwise comparison.

**Supplementary Table S2**. MapMan enrichment list of DAPs in pairwise comparison.

**Supplementary Table S3**. KEGG pathway enrichment list of DAPs in pairwise comparison.

**Supplementary Table S4**. Gene ontology enrichment list of DAPs in time-course comparison.

**Supplementary Table S5**. MapMan enrichment list of DAPs in time-course comparison.

**Supplementary Table S6**. KEGG pathway enrichment list of DAPs in time-course comparison.

**Supplementary Table S7**. MapMan annotation differences in response to osmotic stress between DQ and XL. Green represents enriched terms in downregulated proteins. Red represents enriched terms in upregulated proteins. NA represents a functional entry that is not statistically significant or not available.

**Supplementary Table S8**. Gene ontology annotation differences in response to osmotic stress between DQ and XL. Green represents enriched GO terms in downregulated proteins. Red represents enriched GO terms in upregulated proteins. NA represents a GO entry that is not statistically significant or not available.

giaa019_GIGA-D-19-00339_Original_Submission

giaa019_GIGA-D-19-00339_Revision_1

giaa019_Response_to_Reviewer_Comments_Original_Submission

giaa019_Reviewer_1_Report_Original_Submission KosovÃi KlÃira -- 10/22/2019 Reviewed

giaa019_Reviewer_2_Report_Original_Submission N.P. Singh -- 11/6/2019 Reviewed

giaa019_Supplemental_Figures_and_Tables

## Abbreviations

ABA: abscisic acid; AGC: automatic gain control; AMPK: AMP-activated protein kinase; APX: ascorbate peroxidase; ATP: adenosine triphosphate; BLAST: Basic Local Alignment Search Tool; CK: control group; CV: coefficient of variation; DAP: differentially abundant protein; DDA: data-dependent acquisition; DIA: data-independent acquisition; FDR: false discovery rate; GO: Gene Ontology; GPX: glutathione peroxidase; GRN: gene regulatory networks; GRP: glycine-rich RNA-binding protein; iRT: indexed retention time; KEGG: Kyoto Encyclopedia of Genes and Genomes; LC-MS: liquid chromatography–mass spectrometry; MAPK: mitogen-activated protein kinase; mRNA: messenger RNA; MS: mass spectrometry; NCBI: National Center for Biotechnology Information; PCA: principal component analysis; PEG: polyethylene glycol; Prxs: peroxiredoxins; QC: quality control; ROS: reactive oxygen species; SA: salicylic acid; ST: treatment group.

## Competing Interests

The authors declare that they have no competing interests.

## Funding

This work was supported by The Financial Special Fund (2017CZZX001, XZNKY-2018-C-021).

## Authors’ Contributions

H.Y. and Y.W. conceived the idea of the work and designed the research; Z.S., Q.X., and S.X. produced and analysed the data; Q.X., Y.W., D.J., and Z.S. managed the samples and the data; H.Y., Y.W., and Q.X. wrote the paper; and X.Z. and Y.W. revised the manuscript. All authors read and approved the final manuscript.
