# Supplementary material for: Comparative proteomics analysis of Tibetan hull-less barley under osmotic stress via data-independent acquisition mass spectrometry
Source: Gigascience. 2020 Mar 3;9(3):giaa019. doi: 10.1093/gigascience/giaa019 (PMC7053489; doi:10.1093/gigascience/giaa019)
Supplement: giaa019_GIGA-D-19-00339_Original_Submission [file giaa019_giga-d-19-00339_original_submission.pdf]

GigaScience

Comparative Proteomics Analysis of Tibetan Hulless Barley under Drought Stress via Data-Independent Acquisition Mass Spectrometry

--Manuscript Draft--

**Manuscript Number:**	GIGA-D-19-00339
**Full Title:**	Comparative Proteomics Analysis of Tibetan Hulless Barley under Drought Stress via Data-Independent Acquisition Mass Spectrometry
**Article Type:**	Research
**Funding Information:**	The Financial Special Fund (2017CZZX001, XZNKY-2018-C-021) — Not applicable
**Abstract:**	Background<br><br>Tibetan hulless barley (Hordeum vulgare L. var. nudum) is one of the primary crops cultivated in the mountains of Tibet suffering from low temperature, high salinity, and drought. Specifically, drought is one of the major abiotic stresses that affect and limit Tibetan barley growth. Thus, it's critical to explore the molecular mechanism of hulless barley during arid or drought environmental conditions for improving crop yield.<br><br>Findings<br><br>Here, we employed quantitative proteomics by data-independent acquisition mass spectrometry (DIA-MS) to investigate protein expression in tolerant (XL) and sensitive (DQ) cultivars. A total of 6921 proteins are identified and quantified in all samples. Two distinct strategies, based on pairwise and time-course comparisons, were utilized in the comprehensive analysis of differentially expressed proteins. Further functional analysis of differentially expressed proteins revealed that some hormone metabolism associated and ABA-induced genes that are primarily affected under drought stress. Moreover, we found some regulators, such as GRF, PR10, MAPK and AMPK, were centrally positioned in the gene regulatory network, suggesting they may have a dominant role in the drought response of Tibetan barley.<br><br>Conclusions<br><br>Our findings highlight a subset of proteins and processes that are involved in alleviation of drought stress. In addition, this study provides a large scale and multi-dimensional proteomic data resource for the further survey and improvement of drought tolerance in hulless barley or other plant species as well.
**Corresponding Author:**	Hongjun Yuan<br><br>CHINA
**Corresponding Author Secondary Information:**	
**Corresponding Author's Institution:**	
**Corresponding Author's Secondary Institution:**	
**First Author:**	Yulin Wang
**First Author Secondary Information:**	
**Order of Authors:**	Yulin Wang<br>Zha Sang<br>Shaohang Xu<br>Qijun Xu<br>Xingquan Zeng

Powered by Editorial Manager® and ProduXion Manager® from Aries Systems Corporation

	Dunzhu Jabu
	Hongjun Yuan
**Order of Authors Secondary Information:**	
**Additional Information:**	
**Question**	**Response**
Are you submitting this manuscript to a special series or article collection?	No
**Experimental design and statistics**<br><br>Full details of the experimental design and statistical methods used should be given in the Methods section, as detailed in our Minimum Standards Reporting Checklist. Information essential to interpreting the data presented should be made available in the figure legends.<br><br>Have you included all the information requested in your manuscript?	Yes
**Resources**<br><br>A description of all resources used, including antibodies, cell lines, animals and software tools, with enough information to allow them to be uniquely identified, should be included in the Methods section. Authors are strongly encouraged to cite Research Resource Identifiers (RRIDs) for antibodies, model organisms and tools, where possible.<br><br>Have you included the information requested as detailed in our Minimum Standards Reporting Checklist?	Yes
**Availability of data and materials**<br><br>All datasets and code on which the conclusions of the paper rely must be either included in your submission or deposited in publicly available repositories (where available and ethically appropriate), referencing such data using	Yes

a unique identifier in the references and in the “Availability of Data and Materials” section of your manuscript.<br><br>Have you have met the above requirement as detailed in our Minimum Standards Reporting Checklist?	

Manuscript Click here to download Manuscript manuscript.docx

Comparative Proteomics Analysis of Tibetan Hulless Barley under Drought Stress via Data-Independent Acquisition Mass Spectrometry

Yulin Wang[1,2‡], Zha Sang[1,2‡], Shaohang Xu[3], Qijun Xu[1,2], Xingquan Zeng[1,2], Dunzhu Jabu[1,2], Hongjun Yuan[1,2*]

[1]State Key Laboratory of Hulless Barley and Yak Germplasm Resources and Genetic Improvement, Lhasa 850002, China.

[2]Institute of Agricultural Research, Tibet Academy of Agricultural and Animal Husbandry Sciences, Lhasa 850002, China.

[3]Deepxomics Co., Ltd., Shenzhen 518000, China.

‡ These authors contributed equally to this work.

∗Correspondence address: Hongjun Yuan, Tibet Academy of Agricultural and Animal Husbandry Sciences, Lhasa 850002, China. E-mail: yhjxzls@sina.com.

Abstract

Background

Tibetan hulless barley (Hordeum vulgare L. var. nudum) is one of the primary crops cultivated in the mountains of Tibet suffering from low temperature, high salinity, and drought. Specifically, drought is one of the major abiotic stresses that affect and limit Tibetan barley growth. Thus, it's critical to explore the molecular mechanism of hulless barley during arid or drought environmental conditions for improving crop yield.

Findings

Here, we employed quantitative proteomics by data-independent acquisition mass spectrometry (DIA-MS) to investigate protein expression in tolerant (XL) and sensitive (DQ) cultivars. A total of 6921 proteins are identified and quantified in all samples. Two distinct strategies, based on pairwise and time-course comparisons, were utilized in the comprehensive analysis of differentially expressed proteins. Further functional analysis of differentially expressed proteins revealed that some hormone metabolism associated and ABA-induced genes that are primarily affected under drought stress. Moreover, we found some regulators, such as GRF, PR10, MAPK and AMPK, were centrally positioned in the gene regulatory network, suggesting they may have a dominant role in the drought response of Tibetan barley.

Conclusions

Our findings highlight a subset of proteins and processes that are involved in alleviation of drought stress. In addition, this study provides a large scale and multi-dimensional proteomic data resource for the further survey and improvement of drought tolerance in hulless barley or other plant species as well.

Keywords

Tibetan hulless barley; drought stress; proteomics; DIA; quantification; abiotic stress

Background

Plant growth is often affected by several environmental abiotic or biotic stresses, which induce various biochemical and physiological responses in plants[1]. Among the abiotic stresses, drought is one of the most prevalent and complex environmental threats presently to affect agriculture[2]. The droughty agricultural areas are estimated to be double by the end of the 21st century[3]. Severe drought can result in a significant reduction in crop yields due to adverse impact on plant growth and development[4]. Lesk et al. used a statistical method to examine disasters from 1964 to 2007 and reported that drought and extreme heat environmental conditions would significantly reduce national cereal production by 9–10%[5]. Therefore, it is extremely urgent to develop drought-tolerant and well-adapted cultivars under water deficit condition for the improvement of the crop yield[3].

To cope with drought stress, plants have developed a variety of mechanisms to confront threats from adverse environmental factors. Their adaptive responses are dynamic and contain both reversible and irreversible changes, including alterations of membranes, changes in cell wall architecture, or adjustments in mitosis[6-8]. In addition, drought can trigger a variety of physiological or biological responses for their adaptation to arid environments. These responses include stomatal closure, inhibition of cell growth, regulation of photosynthesis and adjustment of respiration[9]. Plants have also evolved various mechanisms to overcome water-limited condition at both the cellular and molecular levels, such as accumulation of osmolytes or antioxidants[10]. Besides, previous review reported that phytohormone abscisic acid (ABA) core signaling pathway could mediate several rapid responses to improve tolerance in drought condition, including gene regulation, stomatal closure, and plant growth modulation[11]. Until now, many genes have been recognized and shown to function in stress conditions. These genes consist of transcription factors (AREB, NAC, bZIP, MYC, and MYB) and signaling protein kinases (mitogen activated protein kinases (MAPK), receptor protein kinase, transcription regulation protein kinase., calcium-dependent protein kinase and ribosomal protein kinase)[1, 12-16].

The Tibetan hulless barley (Hordeum vulgare L. var. nudum), also named as “Qingke” in Chinese, is a major cereal grain grown in the Tibetan Plateau. Zeng et al. have completed the draft genome of Tibetan hulless barley and provided some viewpoints about its adaptation to harsh environments on the highland[17]. And two transcriptome datasets were generated to explore the expression changes in Nitrogen deprivation [18] and drought stress[19], respectively. Unlike the genomic research, however, no large-scale proteomic research was performed in Tibetan hulless barley under drought stress. The role of proteins in plant signaling response is critical since proteins directly participate in many aspects of the life cycle. Furthermore, protein expression relies not only on the level of the corresponding mRNA but also on a series of transcriptional and translational regulations[20, 21]. Indeed, mRNA expression is not always a good

predictor of protein expression because low correlations between mRNA and protein expression levels are often observed[22]. Thus, protein, as the direct performer or effector of life activity, would probably provide the most relevant evidence for characterizing a biological system.

In recent years, data independent acquisition mass spectrometry (DIA-MS) has emerged as an important technique in quantitative proteomics[23, 24]. Compared with shotgun proteomics in data dependent acquisition mode, DIA could offer a potentially deeper coverage of the data in shorter analysis time. And its data show fewer missing values, higher precision and better reproducibility across replicates[25]. In this study, we utilized this method to perform a comprehensive proteomic profiling of Tibetan hulless barley. Time-course and pairwise comparison in each time point were conducted with the expression information of all samples, respectively. And then, we examined the physiological or biological processes of differentially expressed proteins (DEPs) in each comparison. Our analysis revealed several important stress responsive genes and function modules, for instance, some hormone metabolism including ethylene, salicylic and cytokinin; cell wall or cell architecture associated with membrane stability; and ABA-induced signaling genes. We further selected some known drought stress responsive genes from public databases or articles and explored their distribution curves in each time point. Lastly, using a machine-learning approach, we constructed a gene regulatory network and revealed several key regulatory elements associated with drought stress tolerance.

Data Description

Plant materials and cultivation

Two Tibetan hulless barley inbred lines, drought-sensitive (DQ) and drought-resistant (XL), were used for our experiments. Seeds of the two cultivars were sown with nutritional soil and maintained in plant growth incubators at 25°C, 2000 μmol.m^{-2}.s^{-1}. In the 2-3 leaf stage, seedlings were removed from the tray and cultivated in half strength Hoagland's nutrient solution[26]. Specifically, polyethylene glycol (PEG) solutions with a concentration of 21% were used for drought stress induction. For each cultivar, half of the plants were replaced with PEG6000 embedding medium after seven days of growth. Next, fresh leaves from two cultivars in control group (CK) and stress treatment group (ST) were sampled at 1, 4, 8, 12, 24, and 48 h, respectively. For the individual plants with specific sampling time point and treatment, three replicates were collected and then kept at -80°C until their analyses.

Protein extraction and digestion

For each plant tissue sample, a 1g subsample was weighed and homogenized by grinding in liquid nitrogen. The powdered samples were moved to 50 ml tubes with 25 ml precooled acetone (-20 °C) containing 10% (v/v) trichloroacetic acid (TCA) and 10 mM dithiothreitol (DTT) was added. After thorough mixing, the homogenate was precipitated overnight at -20 °C and then centrifuged (20,000×g, 4 °C) for 30 min. The pellet was then washed twice with 20 ml chilled acetone (-20 °C) and left at -20 °C for 30 min followed by centrifugation (20,000×g, 4 °C) for 30 min. The precipitation was dissolved with lysis buffer (4% SDS, 100 mM Tris-HCl, 10 mM DTT, pH 8.0) and sonicated for 5 min at 60 W (5 s sonication followed by 10 s break) followed by 30 min centrifugation (20,000×g, 20 °C). The supernatant was collected and protein concentration in the lysate was estimated using bicinchoninic acid (BCA) protein assay kit (Beyotime Institute of Biotechnology, China).

Protein digestion was conducted using the FASP (filter-aided sample preparation) procedure[27]. In brief, proteins extract in an ultrafiltration filtrate tube (30 kDa cut-off, Sartorius, Germany) were mixed with 200 µl UA buffer (8 M urea, 150 mM Tris-HCl, pH 8.0) and centrifuged at 14,000 g at 20°C for 40 min. Samples were washed twice by adding 200 µl UA to the filter unit and centrifuged at 14,000 g at 20 °C for 40 min. After discarding the flow-through from the collection tube, 100 µl IAM solution (10 mM IAM in UA buffer) was added into the filter tube and incubate for 30 min. Samples were washed twice with 100 µl of UA to the filter unit. After centrifuging with 14,000 g at 20°C for 40 min, 100 µl of ABC (0.05M NH4HCO3 in water) was added into the filter unit and centrifuge at 14,000 g. The protein suspension in the filtrate tube was subjected to enzyme digestion with 40 µl ABC with trypsin (Promega, USA) and incubated for 18 h at 37°C. The filtrate was used for LC-MS analysis after centrifuge at 14,000 g for 10 min. QC mixture was formed by pooling equal amounts of peptides from all individuals, which was used to evaluate the reproducibility of the quantitative LC-MS analysis

Peptide fractionation by High-pH RP

Digested peptides were separated on a LC-20AB HPLC system (Shimadzu, Kyoto, Japan) with a High-pH Reversed-Phase (High-pH RP) column (Phenomenon, Torrance, CA). Peptides were eluted at a flow rate of 0.8 mL/min. Buffer A consisted of 10 mM Ammonium acetate (pH 10.0) and buffer B consisted of 10 mM Ammonium acetate, 90% v/v acetonitrile (pH 10.0). The following gradient was applied to perform separation: 100% buffer A for 40 min, 0–5% buffer B for 3 min, 5–35% buffer B for 30 min, 35–70% buffer B for 10 min, 70–75% buffer B for 10 min, 75–100% buffer B for 7 min, 100% buffer B for 15 min and finally 100% buffer A for 15 min. The elution process was monitored by measuring absorbance at 214 nm, and fractions were

collected every 75 s. Finally, collected fractions (approximately 40) were combined into 12 pools. Each fraction was concentrated via vacuum centrifugation and was reconstituted in 40 µl of 0.1% v/v formic acid. All samples were stored at -20 °C until further analysis.

LC mass spectrometry analysis

Peptides were separated with Dionex UltiMate 3000 RSLCnano system with Acclaim PepMap C18 (3 µm, 100 Å, 75 µm x 50 cm) and emitted into a Thermo Q-Exactive HF tandem mass spectrometer. Solvent A was 0.1% formic acid in water, while solvent B was 0.1% formic acid in 98% acetonitrile. For each injection, 3 µl (approximately 3 µg) was loaded and eluted using a 90-minute gradient from 5 to 35% B, followed by a 40 min washing gradient. Data were acquired using either data-dependent acquisition (DDA) or data-independent acquisition (DIA).

For library generation, the Thermo Q-Exactive HF was set to positive mode in a top-20 configuration to acquire data in DDA mode. Precursor spectra (375–1400 m/z) were collected at 120,000 resolution to hit an AGC target of 3e6. The maximum inject time was set to 20 ms. Fragment spectra were collected at 30,000 resolution to hit an AGC target of 1e5 with a maximum inject time of 60 ms. The isolation width was set to 1.6 m/z with a normalized collision energy of 25. Only precursors charged between +2 and +6 that achieved a minimum AGC of 2e3 were acquired. Dynamic exclusion was set to 30s and to exclude all isotopes in a cluster.

For quantitative samples, the Thermo Q-Exactive HF was configured to acquire 55 × 16 m/z DIA spectra (16 m/z precursor isolation windows at 30,000 resolution, AGC target 1e6, maximum inject time 55 ms). Precursor spectra (400–1250 m/z) were collected at 120,000 resolution to hit an AGC target of 3e6. The maximum inject time was set to 50 ms. To evaluate the reproducibility of the LC-MS system during the whole DIA acquisition, the QC sample was measured every 10 samples.

Library generation and quantitative data analyses

MaxQuant (version 1.6.2.6) software[28, 29] was used to analyze the DDA MS/MS data with the following settings: enzyme: Trypsin/P; maximum missed cleavages: 2; fixed modification: carbamidomethyl (C); variable modifications: oxidation (M) and acetyl (protein N-term); precursor mass tolerance: 20 ppm; fragment mass tolerance: 0.05Da; second peptide search was enabled. All other parameters are in default. The MS/MS data were searched against the hordeum vulgare (Barley) protein sequences which were downloaded from Uniprot database (version 2018.7, 210,953 entries), appended with the Biognosys iRT peptide sequences. The FDR threshold was set as 1% at both PSM (peptide spectrum match) and protein levels. Subsequently, the MaxQuant

search result was imported into Spectronaut Pulsar (12.0.20491.4, Biognosys, Schlieren, Switzerland) to generate a spectra library with the default settings.

Spectronaut Pulsar was used to analyze the DIA data with the spectra library based on DDA MS/MS data. Local regression normalization was used for protein quantification normalization. Dynamic MS1 and MS2 mass tolerance strategy was applied for data extraction with correction factor 1. A dynamic XIC RT extraction window with a local non-linear iRT strategy was chosen for calibration. Interference correction was enabled to automatically remove fragments that interfere with other ions across several runs. The decoy method in the feature identification was configured as 'mutated', with decoy limit strategy of 'dynamic' and library size fraction of 0.1. The results were filtered by 1% FDR, and only those protein groups which passed this filter criteria were used in downstream analysis. The DIA raw data and the corresponding results were deposited to iProX database[30].

Bioinformatic data analysis

Statistical analysis was performed in R (version 3.5.0). Hierarchical clustering was performed using pheatmap package. Principal component analysis (PCA) was performed using FactoMineR package. T test was used for statistical differential analysis and a cut of p-value <= 0.05 and fold change >= 2 was used to select statistically differential expressed proteins. Hypergeometric-based enrichment analysis with KEGG[31], Gene Ontology[32, 33] and MapMan[34-36] were performed to annotate protein sequences, individually. For network analysis, the target genes of plant transcription factors (TFs) and protein kinases (PKs) are classified by iTAK program[37]. The Arboreto computational framework integrated with GRNBoost2[38] algorithm was used to reconstruct relevant regulatory relationships in each ecotype. The igraph package was used to visualize networks.

Analyses

Quality control analysis of the Barley's proteome

In this study, we identified a total of 6921 proteins with 1% FDR in all samples with maximum quantified 6313 proteins in a single non-QC sample (i.e. treatment group of XL 48-hour stage, replicate #II, Figure S1). The MS platform was stable and repeatable as evaluated by quality control runs during the entire data-collecting period. The coefficient of variation (CV), reflecting the magnitude of protein expression variability, accounted on average for 20% in each sample (Figure S2a), The relationship between CV and the log area was illustrated in Figure S2b, and proteins were assorted into 12 intervals according to their log area values in descending order. The results showed that

proteins with high intensity always showed small CVs and this is in accordance with previous study [39]. The hierarchical clustering-based heatmap and principal component analysis (PCA) based on quantified protein abundances in each sample were used for further quality control, as illustrated in Figure S2c and Figure S2d, the nine QC samples were clustered together, which indicates that the MS platform was stable and the quality of DIA data was high.

Pairwise Differential Expression Analysis

To explore proteins associating with drought, two types of analysis were performed. According to the time point experiment design of XL and DQ, all cultivars were divided into 10 comparison groups (Figure 1). Each group consists of a treatment-control pair and the relative fold change of protein was calculated for each paired group. The statistical significance of the observed fold change was determined by paired t test for all the DEPs, and the threshold of p-value ≤ 0.05 and fold change≥2 was used. As shown in Figure 2, the DEP numbers varied significantly in different time points, together with a relatively low number of common changes (yellow area), indicates highly diverse dynamics of protein expression regulation in XL and DQ. Compared with down-regulated proteins, more up-regulated proteins were found at time point 4 and 8. This result showed that more proteins were significantly up-regulated in the early stage of drought stress, which may indicate that a sequential activation or repression of specific cellular processes that differ substantially between control and treatment group.

To explore the biological processes in each DEP group, we conducted the hypergeometric-based enrichment analysis based on Mapman and gene ontology (GO) databases. The threshold of an adjusted p-value <= 0.01 was used to define significantly enriched annotation categories. To highlight the key function terms, we manually reviewed the biological function terms in supplementary Table S1, S2 and S3. Functional descriptions associated with potential drought and specific in DL or XL sample were selected and labeled beside the related bars with short abbreviation. Particularly, “cytokinin synthesis degradation”, “UDP glucosyl and glucoronyl transferases” and “leaf senescence” were the dominant responses in DEPs of DQ cultivar, whereas “MAP kinases” and “ethylene synthesis degradation” were two key terms in DEPs of XL cultivar. More details could be obtained through the table below the diagram in Figure 2.

To further explore the DEP's biological functions, we divided the up-regulated and down-regulated genes as independent gene sets and re-annotated them separately. Based on the Mapman annotations, we used a hierarchical heatmap to represent the relationship between time stages and relevant annotated entries. As shown in Figure 3, the color scale was graded to reflect the enrichment scores (log2-transformed FDR). Among these function terms, two hormone metabolism terms, “abscisic acid induced-regulated-responsive-activate” and “ethylene.synthesis-

degradation” were significantly enriched in the XL’s up-regulated gene set at 8h. Another hormone metabolism term, “salicylic acid synthesis-degradation”, was up-regulated in the sensitive cultivar (DQ) at 4h. Moreover, some proteins involved in cell wall formation were up-regulated in the 24-hour stage of XL cultivar, and some proteins involved in wax biosynthesis were also up-regulated in the early 8-hour stage of DQ cultivar. Interestingly, cytochrome P450, an important protein coding gene family involved in growth and drought stress responses[40] was up-regulated in DQ whereas down-regulated in XL cultivar.

We then conducted gene ontology (GO) enrichment analysis. The assigned functions of these genes covered a broad range of GO categories (Figure S3). Specifically, in the biological process category, sulfate assimilation, cellular response to oxidative stress and chitin catabolic process were the major function terms for drought response in DQ cultiva. This suggested that cell wall macromolecule catabolic process, ethylene biosynthetic process, toxin catabolic process and photosynthesis of light harvesting in photosystem I may involve in drought stress tolerance in XL cultivar. For the genes enriched in categories related to cellular component, several photosynthesis terms included photosystem I, photosystem II oxygen evolving complex and photosystem II were upregulated in several stages of XL. For the molecular function category, many potential drought-related genes were classified into a series of redox-related functional items, including glutathione peroxidase activity, glutathione transferase activity, peroxiredoxin activity, pigment binding, chlorophyll binding. This indicated that redox regulation may have great impact on the promotion of drought resistance. Additionally, we identified some chitin-binding proteins related to pathogenesis-related gene family, which may contribute to the defense response of plants under drought [41].

Time-course Differential Expression Analysis

In order to investigate the impact of stress degree differences on protein expression in the consecutive developmental stages over time, stepwise comparisons (e.g. T4 vs. T1, T8 vs. T4, T24 vs. T8, T48 vs. T24, T48 vs. T1) were performed in treatment and control groups of DQ and XL samples separately (Figure 1 and Figure 4). Significantly differential expressed proteins were defined by the cutoff of p-value ≤ 0.05 and fold change ≥ 2. To explorer the relationship of significant DEPs in different treatment of XL and DQ, we generated five Venn diagrams. Next, we carried out the functional characterization of unique DEPs in DQ and XL treatments, individually. Several potential drought-induced entries, unique to DQ or XL treatments, are manually selected and labeled beside the diagram with abbreviation. The complete annotation list could be obtained in the supplementary Tables S4, S5 and S6. For instance, “cytokinin synthesis degradation”, “UDP glucosyl and glucoronyl transferases” and “wax-related” were likely to be an exclusive response in DQ cultivar, whereas “GDSL-motif lipase”, “DUF26 kinase” and “plasma membrane intrinsic protein (PIP)” were three main function terms in XL cultivar.

Core genes in plant defense response

To discover potential drought-related genes, we explored the expression of some well-known genes related to plant defense response, such as ARF, KAT, MAPK, PR10 SnRK2 and WRKY. With Blast alignment, we obtained the Uniprot accession that are relevant to the candidate genes. Next, we examined the individual expression levels of these genes and depicted the expression profiles in Figure 5. Through closer examination of these genes, we found that the MAPK (M0V3Q0) and PR10 (Q84QC7) exhibited higher abundance in treatment group over all the time point of XL and DQ, indicating that the two genes might play important roles in plant defense to drought. Additionally, SnRK2 (M0XX02) and WRKY (B2KJ55) also showed a similar trend, which were up-regulated at 4-hour and 8-hour time points of DQ whereas down-regulated at 24-hour time point of XL.

To further investigate the potential drought tolerance mechanisms, we collected manually curated genes involved in drought stress response from a public database: DroughtDB (http://pgsb.helmholtz-muenchen.de/droughtdb/). We selected best hit for each subject sequence with the threshold of evalue >= 0.00001 and identity>=80% using Blast. Most of the genes were aligned to the genes identified in this study. However, none of these genes showed significant expression change as shown in Figure S4. Furthermore, we selected four water deprivation-related gene ontology (GO) terms, including cellular response to water deprivation, response to water deprivation, response to desiccation and positive regulation of response to water deprivation, and collected the relevant protein sequences from Oryza sativa (rice) annotated by Uniprot. We did similar analysis and as shown in Figure S5, most of genes in the treatment group, compared with control group, showed relatively higher expression level in both DQ and XL cultivars. This result showed that many DEPs were significantly enriched in expected biological processes in response to water deprivation.

Gene regulated network

Considering genes that can form complex dynamical systems or gene regulatory networks could defense drought during plant growth, we explored the co-expression patterns and potential regulatory associations that were represented in Gene Regulatory Networks (GRNs). Specifically, 21 potential stress-responding genes were chosen as the candidate target gene set (Figure 6). Among them, 8 TFs are in the families of Alfin-like, WRKY, MYB-related, bZIP, GRF, bHLH and B3-ARF; 12 genes belong to important drought responsive genes, including ARF, MAPk, SnRK2 and PR10; and one AMP-activated protein kinase (AMPK). Then arboreto takes this target-gene expression matrix as inputs and produces reliable interaction predictions. Based on the expression values of a set of candidate genes, we constructed a partial GRN with regulatory associations using the identified stress-responding genes for each ecotype,

respectively (see Methods). As shown in Figure 6, this result revealed that the bHLH, GRF and PR10 had more connections in DQ than in XL, indicating that these two genes probably play important roles in the drought responsive process of DQ cultivar. In addition, MAPK and SnRK2 showed more connections in XL compared with DL. Remarkably, AMPK was the hub gene with highest connection number in both XL and DQ cultivars.

Discussion

Drought is one of the most acute environmental stresses that directly affects agricultural productivity. In this study, we first utilized DIA-MS based proteomics technology to quantify proteins in different samples and explored essential DEPs in hulless barley over multiple time points under two cultivars. By two different comparison strategies, time-course and pairwise, we conducted a comprehensive analysis to explore protein-level changes in response to drought stresses.

In pairwise comparison analysis, we detected three essential biological function terms related to drought regulation in DQ cultivar. Specifically, cytokinins are a class of growth-promoting hormones regulating various developmental processes, including cell division and senescence [42]. Previous studies revealed that the reduced cytokinin levels could improve drought tolerance by suppressing growth and reduce stomatal density [43, 44]. Additionally, UDP-glucuronosyl/UDP-glucosyltransferases, pronounced change at time 4h of DQ, are a superfamily of enzymes that catalyze glucuronidation reaction [45], and they were found to enhance plant tolerance under a series of adverse environmental factors, including low temperatures, salinity and drought [46].

Furthermore, the “leaf senescence” at time 24h presents accelerated leaf senescence of DQ cultivar and implies DQ cultivar might be more sensitive to drought. By the Mapman annotation as shown in Figure 3, we found that the function term “salicylic acid synthesis-degradation” was up-regulated in the sensitive cultivar (DQ). Salicylic acid has been reported that it could ameliorate the oxidative stress and enhance the plant tolerance to abiotic [47]. Our result further supports that the degradation of salicylic acid would make the DQ cultivar more susceptible to drought stress. The function terms from ABA-dependent and independent signaling pathway exhibited dominant expression changes in XL cultivar. Several studies have proven that ABA plays a key role in regulating the adaptive response of plants under diverse stress conditions [48, 49]. Meanwhile, the plant hormone ethylene is well known to play an essential role in plant growth, development and drought resistance. In particular, lower ethylene levels would lead to higher drought tolerance. Study from Shi et al. also indicated that a reduced sensitivity to ethylene by CRISPR-Cas9 technology would enhance cell elongation and division, thus increasing grain yield under drought condition [50]. We also identified genes relevant to cell wall construction. Since cell wall is the first line

to defense against abiotic stress, many proteins that are involved in cell wall strengthening or cellular membrane stabilization will be significantly regulated under drought stress [51].

In time-course comparison, we identified genes related to cytokinin degradation and UDP-glucuronosyl/UDP-glucosyltransferases in treatment group of DQ cultivar. Moreover, we found some DEPs from DQ cultivar that can produce a secondary metabolite (wax) in time-course comparison analysis, as shown in Figure 4. The increased accumulation of cuticular wax under the drought condition can improve tolerance and reduce water loss [52]. In addition, three manually-reviewed entries were specific in the treatment group of XL cultivar. Of these, Hong et al. demonstrated that GDSL-type lipase can activate susceptibility to disease and tolerance of abiotic stress [53]. Miyakawa et al. demonstrated that a plant-specific cysteine-rich motif (DUF26) may be widely involved in plant-specific responses to biotic and abiotic stresses [54]. And Lu et al. showed that changes in gene expression of some plasma membrane intrinsic proteins (PIP) also can promote drought stress tolerance [55]. Overall, the analyses carried out in this study have confirmed findings reported in previous studies and provided additional evidence of abiotic tolerance in the resistant compared to susceptible cultivars.

To investigate the expression status of several well-known drought stress genes, we found four proteins (MAPK, PR10, SnRK2 and WRKY) had significant expression change between control and treatment groups. Of these, mitogen activated protein kinase (MAPK) cascade is one of the major signaling pathways involved in abiotic stress response in plants [56]. And it is evolutionary conserved among eukaryotic organisms and can transduce extracellular signals to the nucleus under abiotic stress [57, 58]. PR10 gene had been confirmed that the overexpression in rice could enhance drought and salt stress tolerance [59]. Additionally, WRKY transcription factors were thought to participate in regulations of water stress and drought responses [60]. Taken together, these results demonstrated that these genes were potential candidate genes for agricultural application to protect the crops against biotic and abiotic stresses.

Finally, we also found a few genes centrally positioned in gene regulatory network, suggesting that these genes may have a dominant role/regulation in Tibetan hulless barley. Specifically, growth regulating factors (GRFs) and basic helix-loop-helix (bHLH) protein family are plant-specific transcription factors that are involved in diverse biological or physiological processes, such as growth, hormone responses and stress [61, 62]. And AMPK, known to be responsible for the maintenance of ATP balance during energy metabolism [63], occupies the central position in both networks, indicating that it is likely to be a core regulatory component in drought resistance network. Moreover, SnRK2, a serine/threonine kinase specific in plant involved in plant response to abiotic stresses and abscisic acid (ABA)-dependent plant development [64], showed higher abundance in XL than DQ. Furthermore, we also found that MAPK-related genes showed more connections in XL cultivar. Previous studies suggested that

the mitogen-activated protein kinase could be activated by the ABA core signaling module through transcriptional regulation [11]. Thus, we could infer that ABA-induced pathway might have stronger impact on XL than DQ cultivar.

In summary, this proteomic study provided a valuable resource to explore the stress responsive proteins that can help us understand the underlying regulatory mechanisms in Tibetan hulless barley. Furthermore, these data will be valuable to plant biologists who are interested in exploring signaling mechanism to drought, thus helping promote drought stress tolerance in crops.

Availability of supporting data and materials

All of the MS raw data (DIA and DDA) have been deposited to the ProteomeXchange Consortium (http://proteomecentral.proteomexchange.org) via the iProX partner repository[30] with the dataset identifier PXD015597.

Additional files

Supplementary Figure S1. The numbers of identified proteins across different samples.

Supplementary Figure S2a. The distribution of coefficient of variation of protein abundances. The coefficient of variation was calculated for each protein using R (sd(Biological Replicates) / mean(Biological Replicates)).

Supplementary Figure S2b. The relationship between coefficient of variation and protein abundance (log2 transformed). The coefficient of variation was calculated for each protein abundance measurement using R (sd(Biological Replicates) / mean(Biological Replicates)).

Supplementary Figure S2c. Heatmap of protein expression across different samples. The hierarchical clustering is performed using neighbor joining algorithm with a Euclidean distance similarity measurement of the log2 of the protein abundance. The classification for the samples is illustrated with cultivar, process and time at the top of the map.

Supplementary Figure S2d. Principal component analysis (PCA) score plot for proteins in wheat XL and DL under control and drought stress conditions. Each point represents a sample.

Supplementary Figure S3. Gene ontology enriched heatmap for DEPs in pairwise comparison. Similar to Figure 3, but with gene ontology instead of Mapman database.

The left panel shows the annotation of up-regulated proteins and the right panel shows the annotation of down-regulated proteins. Row names are the samples from five time points in DQ and XL cultivars. Column names are the enriched items from three aspects of gene ontology database (biological process: BP; cellular component: CC; and molecular. Function: MF). The legend shows the color scaling with FDR values. See Supplementary Table S1 for the entire list of the GO terms.

Supplementary Figure S4. Gene expression levels of drought-related genes from DroughtDB database. Similar to Figure 5, the labels in the left panel are the description of the related genes. Particularly, the identifier in the left of tilde (~) symbol is the uniprot ID of hordeum vulgare, the identifier in the right of tilde is the gene symbol from DroughtDB, and the description under the tilde is the drought stress related function annotation.

Supplementary Figure S5. Gene expression levels of drought-related genes from gene ontology database. Similar to Figure 5, the labels in the first left panel are the uniprot IDs of hordeum vulgare, and the labels in the second left panel are the functional description from gene ontology with Blast.

Supplementary Table S1. Gene ontology enrichment list for differentially expressed proteins in pairwise comparison.

Supplementary Table S2. Mapman enrichment list for differentially expressed proteins in pairwise comparison.

Supplementary Table S3. KEGG pathway enrichment list for differentially expressed proteins in pairwise comparison.

Supplementary Table S4. Gene ontology enrichment list for differentially expressed proteins in time-course comparison.

Supplementary Table S5. Mapman enrichment list for differentially expressed proteins in time-course comparison.

Supplementary Table S6. KEGG pathway enrichment list for differentially expressed proteins in time-course comparison.

Abbreviations

DIA: data-independent acquisition; DDA: data-dependent acquisition; MS: mass spectrometry; ABA: abscisic acid; DEP: differentially expressed protein; CK: control group; ST: treatment group; PSM: peptide spectrum match; PCA: Principal

component analysis; CV: coefficient of variation; GRN: Gene Regulatory Networks; BLAST: Basic Local Alignment Search Tool; GO: Gene Ontology; KEGG: Kyoto Encyclopedia of Genes and Genomes;

Competing interests

The authors declare that they have no competing interests.

Funding

This work was supported by the following funding sources: The Financial Special Fund (2017CZZX001, XZNKY-2018-C-021).

Authors' contributions

H.Y. and Y.W. conceived the idea of the work and designed the research; Z.S., Q.X. and S.X. produced and analyzed the data; Q.X., Y.W., D.J. and Z.S. managed the samples and the data; H.Y., Y.W. and Q.X. wrote the paper; X.Z. and Y.W. revised the manuscript. All authors read and approved the final manuscript.

References

1. Seki M, Kamei A, Yamaguchi-Shinozaki K and Shinozaki K. Molecular responses to drought, salinity and frost: common and different paths for plant protection. Curr Opin Biotechnol. 2003;14 2:194-9.
2. Pennisi E. Plant genetics. The blue revolution, drop by drop, gene by gene. Science. 2008;320 5873:171-3. doi:10.1126/science.320.5873.171.
3. Deeba F, Pandey AK, Ranjan S, Mishra A, Singh R, Sharma YK, et al. Physiological

and proteomic responses of cotton (Gossypium herbaceum L.) to drought stress. Plant Physiol Biochem. 2012;53:6-18. doi:10.1016/j.plaphy.2012.01.002.

4. Barnabas B, Jager K and Feher A. The effect of drought and heat stress on reproductive processes in cereals. Plant Cell Environ. 2008;31 1:11-38. doi:10.1111/j.1365-3040.2007.01727.x.

5. Lesk C, Rowhani P and Ramankutty N. Influence of extreme weather disasters on global crop production. Nature. 2016;529 7584:84-7. doi:10.1038/nature16467.

6. CRAMER GR. Abiotic stress and plant responses from the whole vine to the genes. Australian Journal of Grape and Wine Research. 2010;16 s1:86-93. doi:10.1111/j.1755-0238.2009.00058.x.

7. Skirycz A and Inzé D. More from less: plant growth under limited water. Current Opinion in Biotechnology. 2010;21 2:197-203. doi:https://doi.org/10.1016/j.copbio.2010.03.002.

8. Cramer GR, Urano K, Delrot S, Pezzotti M and Shinozaki K. Effects of abiotic stress on plants: a systems biology perspective. BMC Plant Biology. 2011;11 1:163. doi:10.1186/1471-2229-11-163.

9. Zlatev Z and Lidon F. An overview on drought induced changes in plant growth, water relations and photosynthesis. 2012.

10. Anjum S, Ashraf U, Tanveer M, Khan I, Hussain S, Shahzad B, et al. Drought Induced Changes in Growth, Osmolyte Accumulation and Antioxidant Metabolism of Three Maize Hybrids. 2017.

11. de Zelicourt A, Colcombet J and Hirt H. The Role of MAPK Modules and ABA during Abiotic Stress Signaling. Trends Plant Sci. 2016;21 8:677-85.

doi:10.1016/j.tplants.2016.04.004.

12. Thomashow MF. PLANT COLD ACCLIMATION: Freezing Tolerance Genes and Regulatory Mechanisms. Annual Review of Plant Physiology and Plant Molecular Biology. 1999;50 1:571-99. doi:10.1146/annurev.arplant.50.1.571.

13. Nambara E and Marion-Poll A. ABSCISIC ACID BIOSYNTHESIS AND CATABOLISM. Annual Review of Plant Biology. 2005;56 1:165-85. doi:10.1146/annurev.arplant.56.032604.144046.

14. Fujita M, Fujita Y, Maruyama K, Seki M, Hiratsu K, Ohme-Takagi M, et al. A dehydration-induced NAC protein, RD26, is involved in a novel ABA-dependent stress-signaling pathway. Plant J. 2004;39 6:863-76. doi:10.1111/j.1365-313X.2004.02171.x.

15. Mukhopadhyay A, Vij S and Tyagi AK. Overexpression of a zinc-finger protein gene from rice confers tolerance to cold, dehydration, and salt stress in transgenic tobacco. Proceedings of the National Academy of Sciences of the United States of America. 2004;101 16:6309. doi:10.1073/pnas.0401572101.

16. Agarwal PK, Agarwal P, Reddy MK and Sopory SK. Role of DREB transcription factors in abiotic and biotic stress tolerance in plants. Plant Cell Rep. 2006;25 12:1263-74. doi:10.1007/s00299-006-0204-8.

17. Zeng X, Long H, Wang Z, Zhao S, Tang Y, Huang Z, et al. The draft genome of Tibetan hulless barley reveals adaptive patterns to the high stressful Tibetan Plateau. Proceedings of the National Academy of Sciences. 2015:201423628. doi:10.1073/pnas.1423628112.

18. Wei Z, Zeng X, Qin C, Wang Y, Bai L, Xu Q, et al. Comparative Transcriptome Analysis

Revealed Genes Commonly Responsive to Varied Nitrate Stress in Leaves of Tibetan Hulless Barley. Frontiers in Plant Science. 2016;7 1067 doi:10.3389/fpls.2016.01067.

19. Zeng X, Bai L, Wei Z, Yuan H, Wang Y, Xu Q, et al. Transcriptome analysis revealed the drought-responsive genes in Tibetan hulless barley. BMC Genomics. 2016;17 1:386. doi:10.1186/s12864-016-2685-3.

20. Gygi SP, Rochon Y, Franza BR and Aebersold R. Correlation between Protein and mRNA Abundance in Yeast. Molecular and Cellular Biology. 1999;19 3:1720-30. doi:10.1128/mcb.19.3.1720.

21. Lu P, Vogel C, Wang R, Yao X and Marcotte EM. Absolute protein expression profiling estimates the relative contributions of transcriptional and translational regulation. Nature Biotechnology. 2006;25:117. doi:10.1038/nbt1270 https://www.nature.com/articles/nbt1270#supplementary-information.

22. Fukao Y. Discordance between protein and transcript levels detected by selected reaction monitoring. Plant Signaling & Behavior. 2015;10 5:e1017697. doi:10.1080/15592324.2015.1017697.

23. Doerr A. DIA mass spectrometry. Nature Methods. 2014;12:35. doi:10.1038/nmeth.3234.

24. Law KP and Lim YP. Recent advances in mass spectrometry: data independent analysis and hyper reaction monitoring. Expert Review of Proteomics. 2013;10 6:551-66. doi:10.1586/14789450.2013.858022.

25. Bruderer R, Bernhardt OM, Gandhi T, Miladinovic SM, Cheng L-Y, Messner S, et al. Extending the limits of quantitative proteome profiling with data-independent

acquisition and application to acetaminophen treated 3D liver microtissues. Molecular & Cellular Proteomics. 2015:mcp.M114.044305. doi:10.1074/mcp.M114.044305.

26. Hoagland DR and Arnon DI. The Water-Culture Method for Growing Plants Without Soil. 1937.

27. Wisniewski JR, Zougman A, Nagaraj N and Mann M. Universal sample preparation method for proteome analysis. Nat Methods. 2009;6 5:359-62. doi:10.1038/nmeth.1322.

28. Tyanova S, Temu T and Cox J. The MaxQuant computational platform for mass spectrometry-based shotgun proteomics. Nature Protocols. 2016;11:2301. doi:10.1038/nprot.2016.136.

29. Cox J and Mann M. MaxQuant enables high peptide identification rates, individualized p.p.b.-range mass accuracies and proteome-wide protein quantification. Nature Biotechnology. 2008;26 12:1367-72. doi:10.1038/nbt.1511.

30. Ma J, Chen T, Wu S, Yang C, Bai M, Shu K, et al. iProX: an integrated proteome resource. Nucleic Acids Res. 2019;47 D1:D1211-D7. doi:10.1093/nar/gky869.

31. Kanehisa M and Goto S. KEGG: kyoto encyclopedia of genes and genomes. Nucleic Acids Res. 2000;28 1:27-30. doi:10.1093/nar/28.1.27.

32. The Gene Ontology C. The Gene Ontology Resource: 20 years and still GOing strong. Nucleic Acids Res. 2019;47 D1:D330-D8. doi:10.1093/nar/gky1055.

33. Ashburner M, Ball CA, Blake JA, Botstein D, Butler H, Cherry JM, et al. Gene ontology: tool for the unification of biology. The Gene Ontology Consortium. Nat Genet. 2000;25

1:25-9. doi:10.1038/75556.

34. Usadel B, Poree F, Nagel A, Lohse M, Czedik-Eysenberg A and Stitt M. A guide to using MapMan to visualize and compare Omics data in plants: a case study in the crop species, Maize. Plant Cell Environ. 2009;32 9:1211-29. doi:10.1111/j.1365-3040.2009.01978.x.

35. Thimm O, Blasing O, Gibon Y, Nagel A, Meyer S, Kruger P, et al. MAPMAN: a user-driven tool to display genomics data sets onto diagrams of metabolic pathways and other biological processes. Plant J. 2004;37 6:914-39.

36. Lohse M, Nagel A, Herter T, May P, Schroda M, Zrenner R, et al. Mercator: a fast and simple web server for genome scale functional annotation of plant sequence data. Plant Cell Environ. 2014;37 5:1250-8. doi:10.1111/pce.12231.

37. Zheng Y, Jiao C, Sun H, Rosli H, Pombo M, Zhang P, et al. iTAK: A Program for Genome-wide Prediction and Classification of Plant Transcription Factors, Transcriptional Regulators, and Protein Kinases. 2016.

38. Moerman T, Aibar Santos S, Bravo Gonzalez-Blas C, Simm J, Moreau Y, Aerts J, et al. GRNBoost2 and Arboreto: efficient and scalable inference of gene regulatory networks. Bioinformatics. 2019;35 12:2159-61. doi:10.1093/bioinformatics/bty916.

39. Molloy M, Donohoe S, E Brzezinski E, Kilby G, Stevenson T, David Baker J, et al. Large-scale evaluation of quantitative reproducibility and proteome coverage using acid cleavable isotope coded affinity tag mass spectrometry for proteomic profiling. 2005.

40. Tamiru M, Undan JR, Takagi H, Abe A, Yoshida K, Undan JQ, et al. A cytochrome

P450, OsDSS1, is involved in growth and drought stress responses in rice (Oryza sativa L.). Plant Mol Biol. 2015;88 1-2:85-99. doi:10.1007/s11103-015-0310-5.

41. Ali M, Luo DX, Khan A, Haq SU, Gai WX, Zhang HX, et al. Classification and Genome-Wide Analysis of Chitin-Binding Proteins Gene Family in Pepper (Capsicum annuum L.) and Transcriptional Regulation to Phytophthora capsici, Abiotic Stresses and Hormonal Applications. Int J Mol Sci. 2018;19 8 doi:10.3390/ijms19082216.

42. Schaller G, Street I and Kieber J. Cytokinin and the cell cycle. 2014.

43. Nishiyama R, Watanabe Y, Fujita Y, Le DT, Kojima M, Werner T, et al. Analysis of Cytokinin Mutants and Regulation of Cytokinin Metabolic Genes Reveals Important Regulatory Roles of Cytokinins in Drought, Salt and Abscisic Acid Responses, and Abscisic Acid Biosynthesis. The Plant Cell. 2011;23 6:2169. doi:10.1105/tpc.111.087395.

44. Farber M, Attia Z and Weiss D. Cytokinin activity increases stomatal density and transpiration rate in tomato. 2016.

45. D King C, Rios G, D Green M and R Tephly T. UDP-Glucuronosyltransferases. 2000.

46. Li P, Li Y-J, Zhang F-J, Zhang G-Z, Jiang X-Y, Yu H-M, et al. The Arabidopsis UDP-glycosyltransferases UGT79B2 and UGT79B3, contribute to cold, salt and drought stress tolerance via modulating anthocyanin accumulation. The Plant Journal. 2017;89 1:85-103. doi:10.1111/tpj.13324.

47. Fayez KA and Bazaid SA. Improving drought and salinity tolerance in barley by application of salicylic acid and potassium nitrate. Journal of the Saudi Society of Agricultural Sciences. 2014;13 1:45-55. doi:https://doi.org/10.1016/j.jssas.2013.01.001.

48. Vishwakarma K, Upadhyay N, Kumar N, Yadav G, Singh J, Mishra RK, et al. Abscisic Acid Signaling and Abiotic Stress Tolerance in Plants: A Review on Current Knowledge and Future Prospects. Frontiers in Plant Science. 2017;8 161 doi:10.3389/fpls.2017.00161.

49. Sah SK, Reddy KR and Li J. Abscisic Acid and Abiotic Stress Tolerance in Crop Plants. Front Plant Sci. 2016;7:571. doi:10.3389/fpls.2016.00571.

50. Shi J, Gao H, Wang H, Lafitte HR, Archibald RL, Yang M, et al. ARGOS8 variants generated by CRISPR-Cas9 improve maize grain yield under field drought stress conditions. Plant Biotechnol J. 2017;15 2:207-16. doi:10.1111/pbi.12603.

51. Bosch M, Mayer CD, Cookson A and Donnison IS. Identification of genes involved in cell wall biogenesis in grasses by differential gene expression profiling of elongating and non-elongating maize internodes. J Exp Bot. 2011;62 10:3545-61. doi:10.1093/jxb/err045.

52. Seo PJ and Park CM. Cuticular wax biosynthesis as a way of inducing drought resistance. Plant Signal Behav. 2011;6 7:1043-5. doi:10.4161/psb.6.7.15606.

53. Hong JK, Choi HW, Hwang IS, Kim DS, Kim NH, Choi DS, et al. Function of a novel GDSL-type pepper lipase gene, CaGLIP1, in disease susceptibility and abiotic stress tolerance. Planta. 2008;227 3:539-58. doi:10.1007/s00425-007-0637-5.

54. Miyakawa T, Hatano K, Miyauchi Y, Suwa Y, Sawano Y and Tanokura M. A secreted protein with plant-specific cysteine-rich motif functions as a mannose-binding lectin that exhibits antifungal activity. Plant Physiol. 2014;166 2:766-78. doi:10.1104/pp.114.242636.

55. Lu L, Dong C, Liu R, Zhou B, Wang C and Shou H. Roles of Soybean Plasma Membrane Intrinsic Protein GmPIP2;9 in Drought Tolerance and Seed Development. Front Plant Sci. 2018;9:530. doi:10.3389/fpls.2018.00530.

56. Sinha AK, Jaggi M, Raghuram B and Tuteja N. Mitogen-activated protein kinase signaling in plants under abiotic stress. Plant Signal Behav. 2011;6 2:196-203. doi:10.4161/psb.6.2.14701.

57. Jonak C, Okresz L, Bogre L and Hirt H. Complexity, cross talk and integration of plant MAP kinase signalling. Curr Opin Plant Biol. 2002;5 5:415-24.

58. Tena G, Asai T, Chiu WL and Sheen J. Plant mitogen-activated protein kinase signaling cascades. Curr Opin Plant Biol. 2001;4 5:392-400.

59. Wu J, Kim SG, Kang KY, Kim JG, Park SR, Gupta R, et al. Overexpression of a Pathogenesis-Related Protein 10 Enhances Biotic and Abiotic Stress Tolerance in Rice. Plant Pathol J. 2016;32 6:552-62. doi:10.5423/PPJ.OA.06.2016.0141.

60. Tripathi P, Rabara R and Rushton P. A systems biology perspective on the role of WRKY transcription factors in drought responses in plants. 2013.

61. Castilhos G, Lazzarotto F, Spagnolo-Fonini L, Bodanese-Zanettini MH and Margis-Pinheiro M. Possible roles of basic helix-loop-helix transcription factors in adaptation to drought. Plant Sci. 2014;223:1-7. doi:10.1016/j.plantsci.2014.02.010.

62. Khatun K, Robin AHK, Park JI, Nath UK, Kim CK, Lim KB, et al. Molecular Characterization and Expression Profiling of Tomato GRF Transcription Factor Family Genes in Response to Abiotic Stresses and Phytohormones. Int J Mol Sci. 2017;18 5 doi:10.3390/ijms18051056.

63. Mihaylova MM and Shaw RJ. The AMPK signalling pathway coordinates cell growth, autophagy and metabolism. Nat Cell Biol. 2011;13 9:1016-23. doi:10.1038/ncb2329.

64. Kulik A, Wawer I, Krzywinska E, Bucholc M and Dobrowolska G. SnRK2 protein kinases--key regulators of plant response to abiotic stresses. OMICS. 2011;15 12:859-72. doi:10.1089/omi.2011.0091.

Figure Legends

Figure 1. Comparison for differentially expressed proteins. The pink arrows indicate the comparison between treatment and control groups. And the yellow arrows indicate the comparison in the consecutive developmental stages over time points.

Figure 2. Down- and up-regulated proteins in DQ and XL cultivars between treatment and control groups. The graph is based on the differential expression analysis by pairwise comparison, showing the number of proteins that are significantly differential expressed. The blue bars represent the DQ samples, and the green bars represent the XL samples. Among them, the dark and light colors denote the up-regulated and the down-regulated proteins, respectively. The yellow area in the center of the bar provides the intersection number of DEPs between XL and DQ. The abbreviations beside the bars are the unique annotated functional entries from XL or DQ, and they are manually selected according to the correlation with the drought resistance.

Figure 3. Mapman enriched heatmap for DEPs in pairwise comparison. The left map shows the annotation of up-regulated proteins and the right map shows the annotation of down-regulated proteins. Row names represent the samples from five time points in DQ and XL cultivars. Column names are the enriched functional categories in Mapman database. The legend shows the color scaling with FDR values. Specifically, the colored cells are the significantly enriched terms with FDR<=0.01, and the grey color cells are not.

Figure 4. Down- and up-regulated proteins in DQ and XL cultivars with comparison over five time points. The pink and orange bars correspond to the control and treatment group of the DQ samples, and the green and blue bars correspond to the control and treatment group of the DQ XL samples. Of these, the dark and light colors denote up-regulated and the down-regulated proteins, respectively. The Venn diagrams shows the overlap of four groups in each comparison. Particularly, each group contains both up-regulated and down-regulated DEPs. The abbreviations beside the circles provide the

unique annotated functional entries of XL treatment or DQ treatment, and they are manually selected according to the correlation with the drought resistance.

Figure 5. Gene expression levels of six core genes in pant defense response. The labels in the first row of top panel are the target gene names and the labels in the second row are the relevant Uniprot accession of hordeum vulgare based on Blast alignment. The biological replicates from the same sample are represented by three different symbols. And the treatment and control groups are illustrated with blue and orange color, respectively. The smooth curves through a set of data points are fitted with loess method.

Figure 6. Regulated network analysis of drought-related genes. The table in the left presents the target gene list used in this analysis. Blue and green nodes in the network correspond to the transcription factors and protein kinases, respectively. The orange nodes are the manually reviewed core genes that are described in Figure 5. And the purple nodes represent the identified genes in XL or DQ cultivars. The size of a node is proportional to its degree. Nodes with higher degree, which means having more neighbors, will have a stronger capacity to modulate adjacent genes than the genes with the lower degrees.

Figure 1

Click here to download Figure Figure 1.pdf

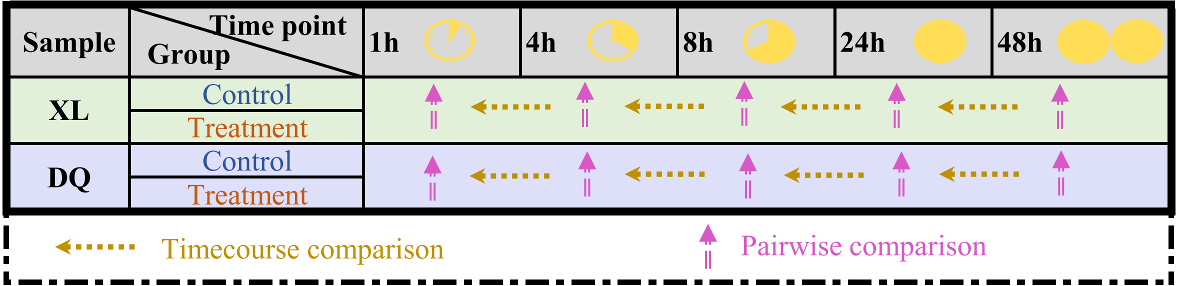

Figure 2

Click here to download Figure Figure 2.pdf

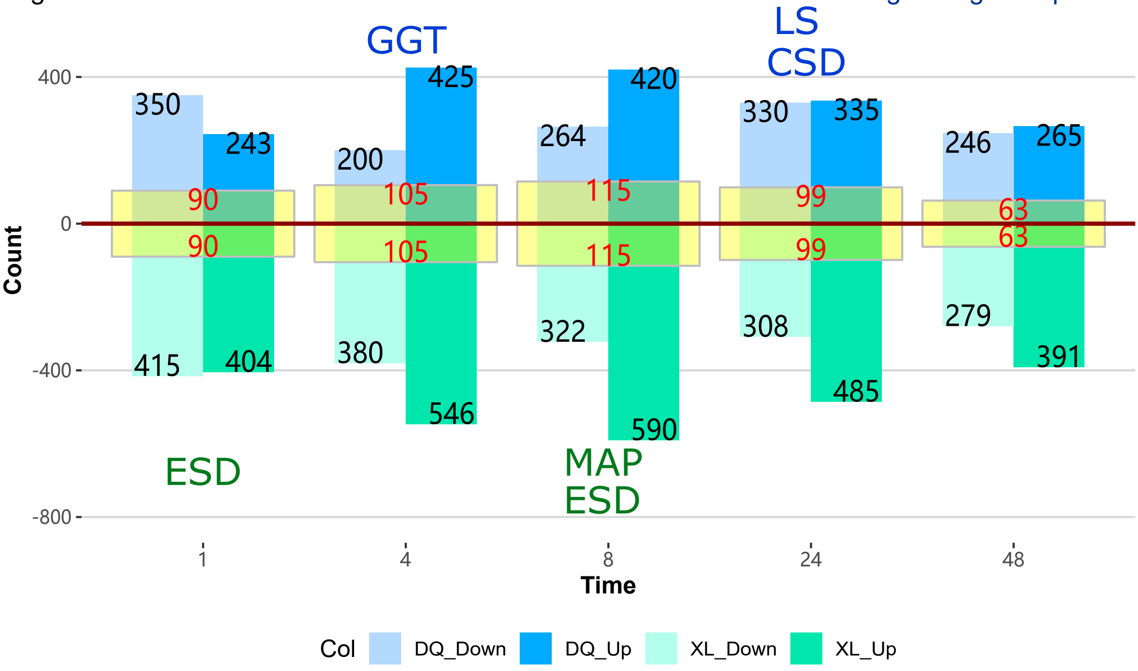

DQ unique DEP	CSD	hormone metabolism.cytokinin.synthesis-degradation
	GGT	misc.UDP glucosyl and glucoronyl transferases
	LS	GO:0010150~BP:leaf senescence
XL unique DEP	MAP	signalling.MAP kinases
	ESD	hormone metabolism.ethylene.synthesis-degradation

Figure 3

Click here to download Figure Figure 3.pdf

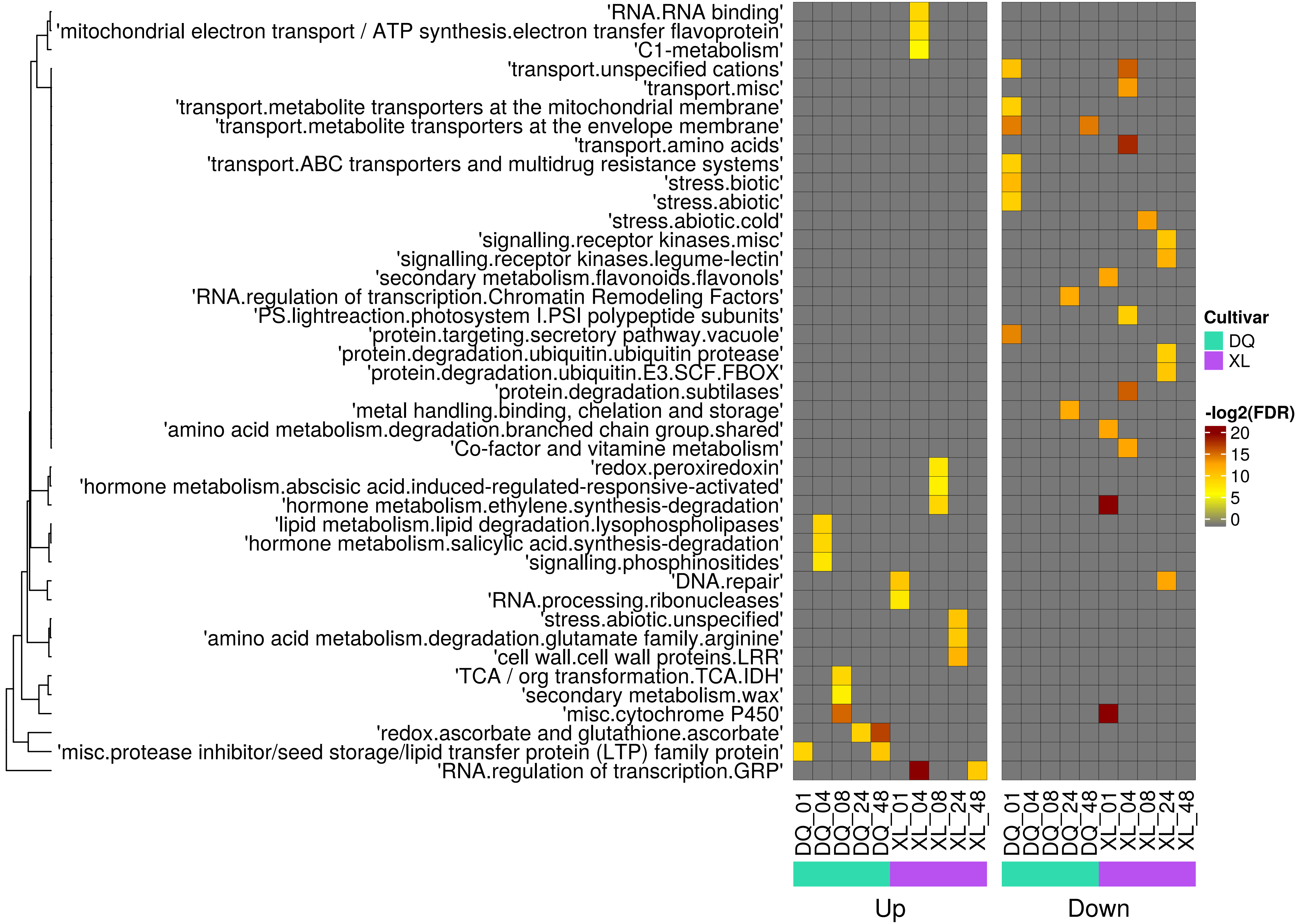

Figure 4

Click here to download Figure Figure 4.pdf

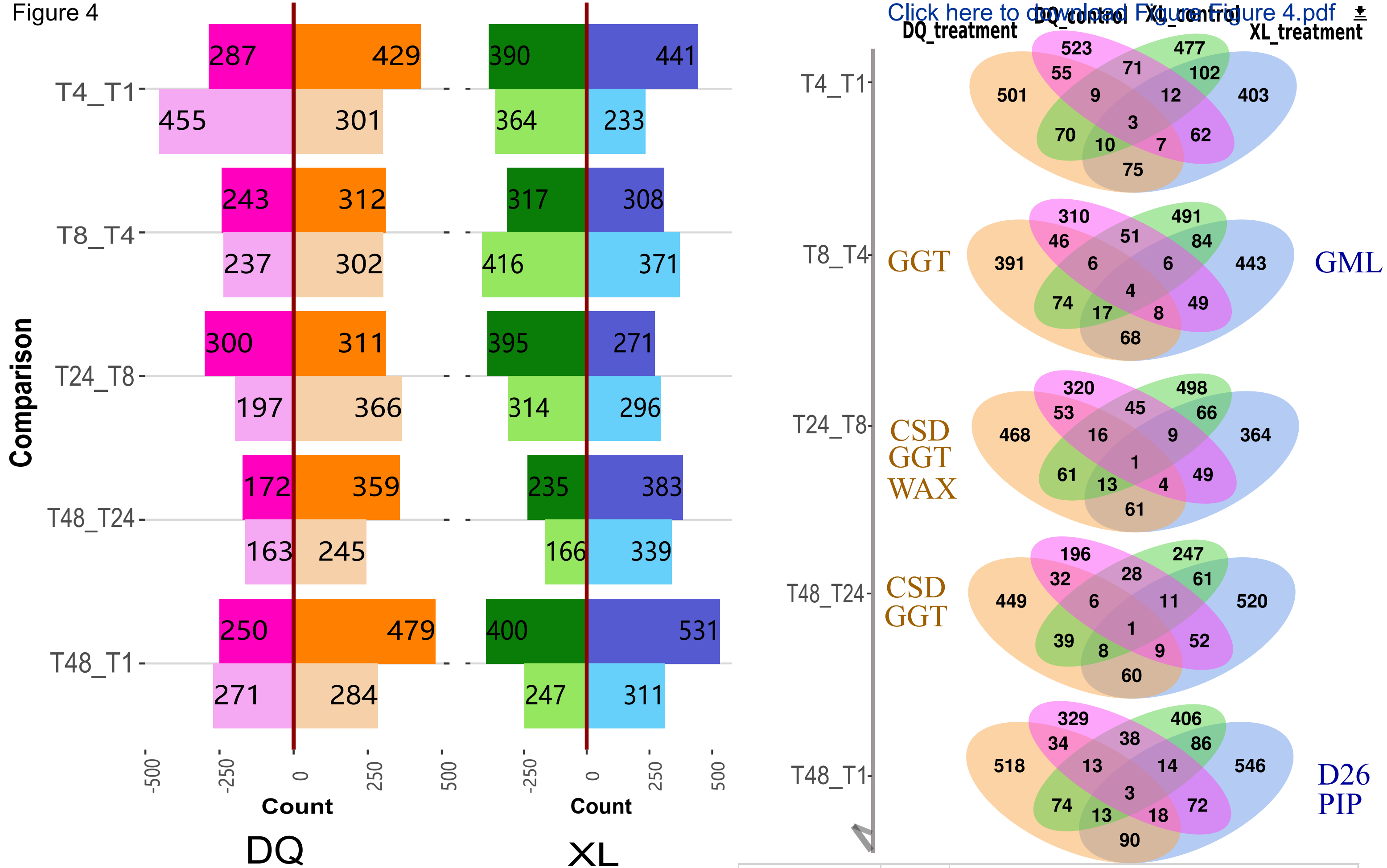

DQ unique DEP	CSD	hormone metabolism.cytokinin.synthesis-degradation
	GGT	misc.UDP glucosyl and glucoronyl transferases
	WAX	secondary metabolism.wax
XL unique DEP	GML	misc.GDSL-motif lipase
	D26	signalling.receptor kinases.DUF 26
	PIP	transport.Major Intrinsic Proteins.PIP

Figure 5

Click here to download Figure Figure 5.pdf

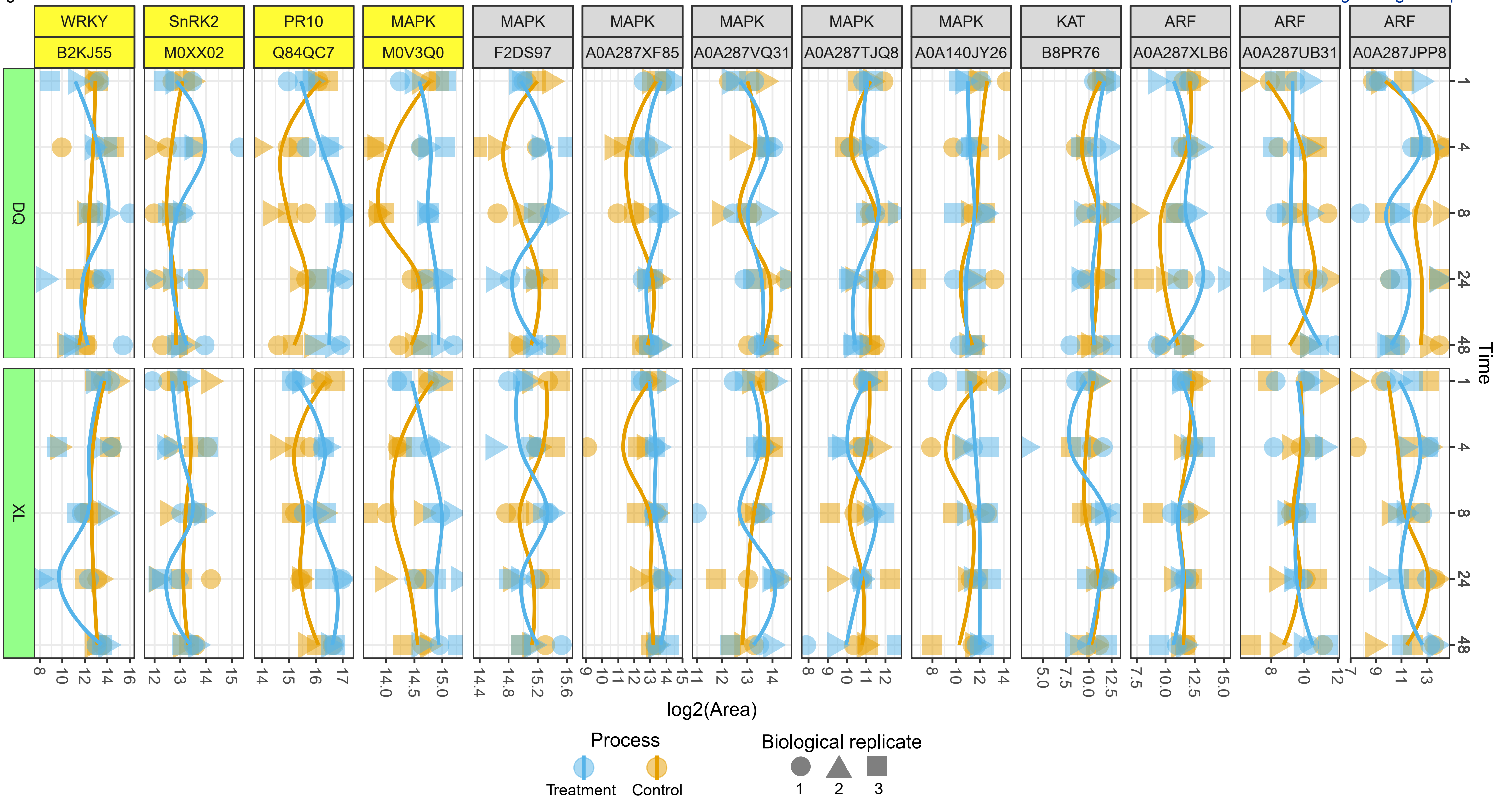

Figure 6

[Click here to download Figure Figure 6.pdf]

Uniprot ID	Class	Gene family
A0A287M8L3	TF	Alfin- like
A0A287FYV5	TF	Alfin- like
B2KJ55	TF	WRKY
A0A287KGP0	TF	MYB- related
A0A287WL34	TF	bZIP
A0A287I173	TF	GRF
A0A287FIG5	TF	bHLH
A0A287XLB6	TF	B3-ARF
A0A287LIF1	PK	CAMK_AMPK
A0A287JPP8	Core	ARF
A0A287UB27	Core	ARF
A0A287XLB6	Core	ARF
A0A287SD70	Core	KAT
A0A140JY26	Core	MAPK
A0A287TJQ8	Core	MAPK
A0A287VQ31	Core	MAPK
A0A287XF85	Core	MAPK
F2DS97	Core	MAPK
M0V3Q0	Core	MAPK
A0A287LYX9	Core	SnRK2
A0A287QNA1	Core	PR10

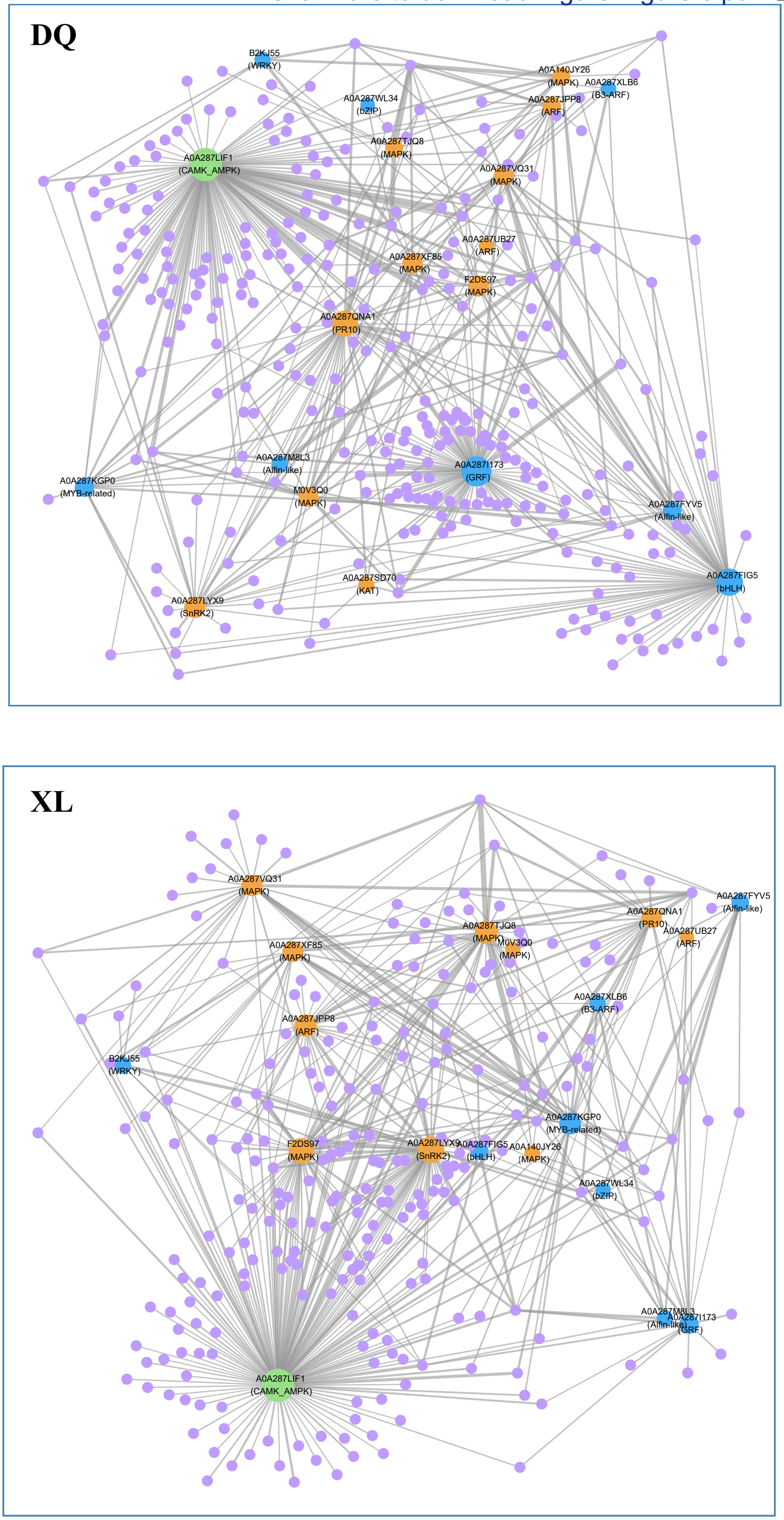

Supplementary Tables

Click here to access/download
Supplementary Material
Supplementary tables.xlsx

Supplementary Figures

Click here to access/download
Supplementary Material
Supplementary_figures.docx

Personal Cover Click here to download Personal Cover cover letter.docx

Dear Editor,

We would like to submit the enclosed manuscript entitled "*Comparative Proteomics Analysis of Tibetan Hulless Barley under Drought Stress via Data-Independent Acquisition Mass Spectrometry*", which we wish to be considered for publication in *GigaScience*. No conflict of interest exits in the submission of this manuscript, and manuscript is approved by all authors for publication. I would like to declare on behalf of my co-authors that the work described was original research that has not been published previously, and not under consideration for publication elsewhere, in whole or in part.

Tibetan hulless barley (Hordeum vulgare L. var. nudum) is one of the primary crops cultivated in the mountains of Tibet suffering from low temperature, high salinity, and drought. Specifically, drought is one of the major abiotic stresses that affect and limit Tibetan barley growth. Thus, it's critical to explore the molecular mechanism of hulless barley during arid or drought environmental conditions for improving crop yield. Here, we employed quantitative proteomics by data-independent acquisition mass spectrometry (DIA-MS) to investigate protein expression in tolerant (XL) and sensitive (DQ) cultivars. Two distinct strategies, based on pairwise and time-course comparisons, were utilized in the comprehensive analysis of differentially expressed proteins. Further functional analysis of differentially expressed proteins revealed that some hormone metabolism associated and ABA-induced genes that are primarily affected under drought stress. Our findings highlight a subset of proteins and processes that are involved in alleviation of drought stress.

In addition, this study provides a large scale and multi-dimensional proteomic data resource for the further survey and improvement of drought tolerance in hulless barley or other plant species as well. Therefore, we believe that GigaScience would be an outstanding forum for this manuscript.

We deeply appreciate your consideration of our manuscript, and we look forward to receiving comments from the reviewers.

Thank you and best regards.

Sincerely yours,

Dr. Hongjun Yuan
Name: Hongjun Yuan
E-mail: yhjxzls@sina.com
Institute: Tibet Academy of Agricultural and Animal Husbandry Sciences, Lhasa 850002, China.
