# Supplementary material for: Comparative proteomics analysis of Tibetan hull-less barley under osmotic stress via data-independent acquisition mass spectrometry
Source: Gigascience. 2020 Mar 3;9(3):giaa019. doi: 10.1093/gigascience/giaa019 (PMC7053489; doi:10.1093/gigascience/giaa019)
Supplement: giaa019_GIGA-D-19-00339_Revision_1 [file giaa019_giga-d-19-00339_revision_1.pdf]

GigaScience

Comparative Proteomics Analysis of Tibetan Hulless Barley under Osmotic Stress via Data-Independent Acquisition Mass Spectrometry

--Manuscript Draft--

**Manuscript Number:**	GIGA-D-19-00339R1
**Full Title:**	Comparative Proteomics Analysis of Tibetan Hulless Barley under Osmotic Stress via Data-Independent Acquisition Mass Spectrometry
**Article Type:**	Research
**Funding Information:**	The Financial Special Fund (2017CZZX001, XZNKY-2018-C-021) — Not applicable
**Abstract:**	Background<br>Tibetan hulless barley (Hordeum vulgare L. var. nudum) is one of the primary crops cultivated in the mountains of Tibet and encounters low temperature, high salinity, and drought. Specifically, drought is one of the major abiotic stresses that affect and limit Tibetan barley growth. Osmotic stress is often simultaneously accompanied by drought conditions. Thus, to improve crop yield, it is critical to explore the molecular mechanism governing the responses of hulless barley to osmotic/drought stress conditions.<br>Findings<br>In this study, we employed quantitative proteomics by data-independent acquisition mass spectrometry (DIA-MS) to investigate protein abundance changes in tolerant (XL) and sensitive (DQ) cultivars. A total of 6921 proteins were identified and quantified in all samples. Two distinct strategies based on pairwise and time-course comparisons were utilized in the comprehensive analysis of differentially abundant proteins. Further functional analysis of differentially abundant proteins revealed that some hormone metabolism-associated and ABA-induced genes are primarily affected by osmotic stress. Enhanced regulation of reactive oxygen species (ROS) may promote the tolerance of hulless barley under osmotic stress. Moreover, we found that some regulators, such as GRF, PR10, MAPK and AMPK, were centrally positioned in the gene regulatory network, suggesting that they may have a dominant role in the osmotic stress response of Tibetan barley.<br>Conclusions<br>Our findings highlight a subset of proteins and processes that are involved in the alleviation of osmotic stress. In addition, this study provides a large-scale and multidimensional proteomic data resource for the further investigations and improvement of osmotic/drought stress tolerance in hulless barley or other plant species.
**Corresponding Author:**	Hongjun Yuan<br><br>CHINA
**Corresponding Author Secondary Information:**	
**Corresponding Author's Institution:**	
**Corresponding Author's Secondary Institution:**	
**First Author:**	Yulin Wang
**First Author Secondary Information:**	
**Order of Authors:**	Yulin Wang<br>Zha Sang<br>Shaohang Xu<br>Qijun Xu<br>Xingquan Zeng<br>Dunzhu Jabu<br>Hongjun Yuan

Powered by Editorial Manager® and ProduXion Manager® from Aries Systems Corporation

**Order of Authors Secondary Information:**	
**Response to Reviewers:**	Dear Editor,<br><br>We are very thankful to the reviewers for their constructive comments to help improve our manuscript. We have considered all comments and suggestions and revised the manuscript accordingly.<br><br>Point-by-point replies are listed as below.<br>Reviewer #1:<br>Terminology:<br>Osmotic stress vs drought stress: The authors used 21% PEG-6000 treatment for simulation of drought stress. However, I recommend to refer to PEG-6000 treatment as an osmotic stress, not drought stress since drought means a lack of water in soil resulting in decreased soil water content while PEG-6000 treatment can reveal differential effects on plants due to its different nature. Thus, to be precise, I recommend to use the term „osmotic stress" instead of „drought" for PEG-6000 treatment.<br>Response: Thank you for your valuable suggestion. We have replaced the “drought” with “osmotic stress” in the revised manuscript according to your suggestion.<br><br>Differentially abundant proteins vs Differentially expresed proteins: I recommend the authors to use the term „differentially abundant proteins" instead of „differentially expressed proteins" since Proteomics methods determine protein relative abundance which always represents a result of two opposite processes, protein biosynthesis („protein expression") and protein degradation.<br>Response: Thank you for your suggestion. We have used “Differentially abundant proteins (DAPs)” instead of “Differentially expressed proteins (DEPs)” in the revised manuscript.<br><br>Materials and methods:<br>In Materials and methods, the source of seeds of the two cultivars of Tibetan hulless barley, drought-senstiive DQ and drought-tolerant XL, has to be given. The authors should write from which institution the seeds were obtained.<br>Response: I’m sorry this was not clearly described in previous version. We have added a sentence in the revised manuscript to clarify the source of seeds as “Specifically, we acquired the DQ cultivar from Institute of Agricultural Research, Tibet Academy of Agricultural and Animal Husbandry Sciences and XL cultivar from The Tibet Autonomous Region Xigaze Agricultural Science Research Institute.”.<br><br>RT-qPCR analysis: In Figure 5, the authors present their original data on gene expression levels of six core genes in plant defense response. However, no basic information on the methodology of RT-qPCR including the sequences of forward and reverse primers and the housekeeping gene is given in Materials and methods. The authors have to add basic information on RT-qPCR methodology corresponding to the results presented in Figure 5.<br>Response: I’m sorry for the inaccurate method descriptions in Figure 5 in previous version. There is no RT-qPCR expeirment in our study. We just used the protein abundance from DIA to generate the smooth curves. In the revised manuscript, we have added a sentence to explain this method as “The abundance curve of target gene was depicted with protein abundance. And a loess method implemented in R environment was used to fit the smooth curves by a set of data points.”.<br><br>In Figure 5 legend, loess method is mentioned for fitting a set of data points with smooth curves; however, no reference on loess method is given in Materials and methods.<br>Response: Thank you for pointing out this problem. I have added the reference of loess method in the revised manuscript.<br><br>Results:<br>I would recommend the authors to add a graphical abstract Figure 7 or a summarising table providing a summary of the differences in response to osmotic stress between the two Tibetan hulless barley cultivars, DQ and XL, at proteome level, based on the results of the present study.<br>Response: Thank you for your valuable suggestion. We have added two

supplementary tables S7 and S8 to summarize the functional differences of XL and DQ in response to osmotic stress.

Discussion:
I think that the authors should discuss their results in a broader context of other proteomic studies focused on drought or osmotic stress response in barley or wheat such as Ford et al. 2011, Wendelboe-Nelson and Morris 2012, Ashoub et al. 2013, Ghabooli et al. 2013, Vítámvás et al. 2015 Frontiers in Plant Science 479, and others.
Response: Thank you for your valuable suggestion. According to your suggestion, we have extended the discussion with several relevant proteomic studies as paragraph 3-5 of Discussion in the revised manuscript.

Formal comments on the text:
Use SI units for volume, i.e., use „dm3" instead of „l", „cm3" instead of „mL" and „mm3" instead of „µl."
Abstract, line 4: Do not use contracted forms in Scientific text, i.e., write „Thus, it is critical to explore…" (not „Thus, it´s critical to explore…").
Background, page 2, line 4: Remove the word „be" in the sentence „The droughty agricultural areas are estimated to double by the end of the 21st century…"
Background, page 3, line 15: Correct the term „salicylic acid" (not „salicylic").
Materials and methods, page 4, line 22: Modify the word form „centrifuge" to „centrifuged" in the sentence „After centrifuging…, 100 mm3 of ABC (0.05 M NH4HCO3 in water) was added into the filer unit and centrifuged at 14,000 g."
Materials and methods, page 4, line 24: Modify the word form in the words „after centrifuge" to „after centrifugation."
Materials and methods, page 4, line 36: Add a comma both preceding and following the word „finally" in the sentence „…and, finally, 100% buffer A for 15 min."
Materials and methods, page 6, line 18: Correct the typing error in the term „heatmap package" (not „pheatmap package").
Analyses, page 7, line Analyses, page 7, line 7: Modify the heading „Pairwise differential abundance analysis" according to my note on DAPs vs DEPs in terminology.
Figure 5 legend, line 1. Correct the typing error in the word „plant" (not „pant") in the term „plant defense response."
Response: Thank you very much for your valuable suggestions. We have revised the text accordingly based on your suggestions, except the content about pheatmap package. The pheatmap package is an implementation of pretty heatmap that offers more control over dimensions and appearance. We are sorry for the missing reference of this package in previous version. We have added the reference of the R package pheatmap in the revised manuscript.
Moreover, in order to improve the grammar and readability, we have asked the professional language service (American Journal Experts, AJE) to edit the text and to reduce the mistakes in English writing as much as possible. The certificate can be verified on the AJE website using the verification code D90D-1336-F7B6-6D7C-9159.

Reviewer #2: The author very well explained the hypothesis, methods and results in the manuscript. The manuscript may be accepted after some minor revisions. The author has to revise the manuscript thoroughly from English editing expert along with the following revisions:
Response: Thank you for the suggestion. We have asked the professional language service (American Journal Experts, AJE) to edit the text and to reduce the mistakes in English writing as much as possible. The certificate can be verified on the AJE website using the verification code D90D-1336-F7B6-6D7C-9159.

Page 5, Paragraph no. 3, line no. 8: Please clarify the statement 'The role of proteins ……………translational regulations'.
Response: We are sorry for the inaccurate description and thank you for pointing out this issue. We have modified this paragraph as “To our knowledge, no large-scale proteomic research of Tibetan hulless barley was performed under drought stress. Indeed, mRNA expression is not always a good predictor of protein abundance because low correlations between mRNA and protein abundance are often observed [20-22]. Thus, the precise measurement of the proteome is meaningful for understanding underlying biological mechanisms of Tibetan hulless barley under osmotic/ drought stress.” in the Introduction of the revised manuscript.

Powered by Editorial Manager® and ProduXion Manager® from Aries Systems Corporation

	Page 6, Paragraph no. 2, line no. 7: Please clarify the statement 'Time-course……….. respectively'.<br>Response: We have modified this statement as "Time-course and pairwise comparison analyses of all samples at each time point were conducted with the protein abundance.".<br><br>Page 8, Paragraph no. 4, line no. 5: Rewrite the text 'To evaluate ……… samples'.<br>Response: We have rewritten this sentence as "To evaluate the reproducibility of the LC-MS system during the whole DIA acquisition, the samples and QCs were analyzed following this scenario: one QC injection followed by 10 experimental samples until all were measured.".<br><br>Page 10, Paragraph no. 2, line no. 10: Please specify the statement 'This results …… treatment group'.<br>Response: Thanks for your suggestion. After careful consideration, we thought this sentence could not provide a meaningful conclusion in that context. So we have decided to delete it in the revised manuscript.<br><br>Page 11, Paragraph no. 3, line no. 4: Rewrite the text 'Significantly ………change≥2'.<br>Response: Thank you for your suggestion. We have rewritten this sentence as "DAPs were selected based on the threshold of protein abundance fold changes ≥ 2 and p-value ≤ 0.05." in the revised paragraph;<br><br>Page 12, Paragraph no. 2, second last line: Explain the statement 'This result showed …. water deprivation '.<br>Response: Thank you for your comment. We are sorry for the inaccurate statement that may lead to misunderstanding. We have rewritten it as "This phenomenon indicates that water deprivation is a vital pathway for both XL and DQ under osmotic stress." in the revised manuscript.<br><br>Page 15, Caption for the supplementary Figure S2d 'Principal component analysis (PCA) score plot for proteins in wheat XL and DL' the word 'wheat' is mentioned and the work has been carried out on the barley. please explain?<br>Response: We are sorry for this typing error. We have corrected it as "'Principal component analysis (PCA) score plot for proteins in XL and DL ……".<br><br>We greatly appreciate your interest and encouragement concerning our manuscript. We look forward to your decision.<br><br>Sincerely yours,<br><br>Dr. Hongjun Yuan<br>Tibet Academy of Agricultural and Animal Husbandry Sciences, Lhasa 850002, China.<br>E-mail: yhjxzls@sina.com.
**Additional Information:**	
**Question**	**Response**
Are you submitting this manuscript to a special series or article collection?	No
**Experimental design and statistics**<br><br>Full details of the experimental design and statistical methods used should be given in the Methods section, as detailed in our Minimum Standards Reporting Checklist. Information essential to interpreting the data presented should be made available in the figure legends.	Yes

Powered by Editorial Manager® and ProduXion Manager® from Aries Systems Corporation

Have you included all the information requested in your manuscript?	
**Resources**<br><br>A description of all resources used, including antibodies, cell lines, animals and software tools, with enough information to allow them to be uniquely identified, should be included in the Methods section. Authors are strongly encouraged to cite Research Resource Identifiers (RRIDs) for antibodies, model organisms and tools, where possible.<br><br>Have you included the information requested as detailed in our Minimum Standards Reporting Checklist?	Yes
**Availability of data and materials**<br><br>All datasets and code on which the conclusions of the paper rely must be either included in your submission or deposited in publicly available repositories (where available and ethically appropriate), referencing such data using a unique identifier in the references and in the "Availability of Data and Materials" section of your manuscript.<br><br>Have you have met the above requirement as detailed in our Minimum Standards Reporting Checklist?	Yes

Powered by Editorial Manager® and ProduXion Manager® from Aries Systems Corporation

Manuscript
Click here to access/download;Manuscript;339-manuscript-HL.docx

Comparative Proteomics Analysis of Tibetan Hulless Barley under Osmotic Stress via Data-Independent Acquisition Mass Spectrometry

Yulin Wang[1,2‡], Zha Sang[1,2‡], Shaohang Xu[3], Qijun Xu[1,2], Xingquan Zeng[1,2], Dunzhu Jabu[1,2], Hongjun Yuan[1,2*]

[1]State Key Laboratory of Hulless Barley and Yak Germplasm Resources and Genetic Improvement, Lhasa 850002, China.

[2]Institute of Agricultural Research, Tibet Academy of Agricultural and Animal Husbandry Sciences, Lhasa 850002, China.

[3]Deepxomics Co., Ltd., Shenzhen 518000, China.

‡ These authors contributed equally to this work.

*Correspondence address: Hongjun Yuan, Tibet Academy of Agricultural and Animal Husbandry Sciences, Lhasa 850002, China. E-mail: yhjxzls@sina.com; phone number:0086-891-6861131.

ORCIDs:

Yulin Wang: 0000-0001-5419-2720
Zha Sang: 0000-0003-2909-420X
Shaohang Xu: 0000-0002-6106-031X
Qijun Xu: 0000-0002-7204-1800
Xingquan Zeng: 0000-0002-1575-3326
Dunzhu Jabu: 0000-0002-8731-5237
Hongjun Yuan: 0000-0002-4552-7952

Abstract

Background

Tibetan hulless barley (*Hordeum vulgare* L. var. *nudum*) is one of the primary crops cultivated in the mountains of Tibet and encounters low temperature, high salinity, and drought. Specifically, drought is one of the major abiotic stresses that affect and limit Tibetan barley growth. Osmotic stress is often simultaneously accompanied by drought conditions. Thus, to improve crop yield, it is critical to explore the molecular mechanism governing the responses of hulless barley to osmotic/drought stress conditions.

Findings

In this study, we employed quantitative proteomics by data-independent acquisition mass spectrometry (DIA-MS) to investigate protein abundance changes in tolerant (XL) and sensitive (DQ) cultivars. A total of 6921 proteins were identified and quantified in all samples. Two distinct strategies based on pairwise and time-course comparisons were utilized in the comprehensive analysis of differentially abundant proteins. Further functional analysis of differentially abundant proteins revealed that some hormone metabolism-associated and ABA-induced genes are primarily affected by osmotic stress. Enhanced regulation of reactive oxygen species (ROS) may promote the tolerance of hulless barley under osmotic stress. Moreover, we found that some regulators, such as GRF, PR10, MAPK and AMPK, were centrally positioned in the gene regulatory network, suggesting that they may have a dominant role in the osmotic stress response of Tibetan barley.

Conclusions

Our findings highlight a subset of proteins and processes that are involved in the alleviation of osmotic stress. In addition, this study provides a large-scale and multidimensional proteomic data resource for the further investigations and improvement of osmotic/drought stress tolerance in hulless barley or other plant species.

Keywords

Tibetan hulless barley; osmotic stress; proteomics; DIA; quantification; abiotic stress

Background

Plant growth is often affected by several environmental abiotic or biotic stresses, which induce various biochemical and physiological responses in plants [1]. Among the abiotic stresses, drought is one of the most prevalent and complex environmental threats presently affecting agriculture [2]. The drought-afflicted agricultural areas are estimated to double by the end of the 21st century [3]. Severe drought can result in a significant reduction in crop yields due to adverse impacts on plant growth and development [4]. Lesk et al. used a statistical method to examine disasters from 1964 to 2007 and reported that drought and extreme heat environmental conditions would significantly reduce national cereal production by 9–10% [5]. Therefore, it is highly important to develop drought-tolerant and well-adapted cultivars under water deficit conditions to improve crop yield [3].

To cope with drought stress, plants have developed a variety of mechanisms to confront threats from adverse environmental factors. The adaptive responses of these plants are dynamic and contain both reversible and irreversible changes, including alterations of membranes, changes in cell wall architecture, and adjustments in mitosis [6-8]. In addition, drought can trigger a variety of physiological or biological responses for their adaptation to arid environments. These responses include stomatal closure, inhibition of cell growth, regulation of photosynthesis and adjustment of respiration [9]. Plants have also evolved various mechanisms to overcome water-limited conditions at both the cellular and molecular levels, such as the accumulation of osmolytes or antioxidants [10]. In addition, a previous review reported that the phytohormone abscisic acid (ABA) core signalling pathway could mediate several rapid responses to improve tolerance in drought conditions, including gene regulation, stomatal closure, and plant growth modulation [11]. To date, many genes have been recognized and shown to function in stress conditions. These genes consist of transcription factors (AREB, NAC, bZIP, MYC, and MYB) and signalling protein kinases (mitogen activated protein kinases, receptor protein kinase, transcription regulation protein kinase, calcium-dependent protein kinase and ribosomal protein kinase) [1, 12-16].

The Tibetan hulless barley (*Hordeum vulgare* L. var. *nudum*, NCBI:txid4513), also named “Qingke” in Chinese, is a major cereal grain grown on the Tibetan Plateau. Zeng et al. completed the draft genome of Tibetan hulless barley and made a number of findings regarding its adaptation to harsh environments on the Tibetan highlands [17]. Next, two transcriptome datasets were generated to explore the expression changes in

nitrogen deprivation [18] and drought stress [19]. To the best of our knowledge, no large-scale proteomic research of Tibetan hulless barley has been performed under drought stress. Indeed, mRNA expression is not always a good predictor of protein abundance because low correlations between mRNA and protein abundance are often observed [20-22]. Thus, the precise measurement of the proteome is meaningful for understanding the underlying biological mechanisms of Tibetan hulless barley under osmotic/drought stress.

In recent years, data-independent acquisition mass spectrometry (DIA-MS) has emerged as an important technique in quantitative proteomics [23, 24]. Compared with shotgun proteomics in data-dependent acquisition mode, DIA could offer a potentially deeper coverage of the data in shorter analysis times. The data obtained through this method show fewer missing values, higher precision and better reproducibility across replicates [25]. In this study, we used polyethylene glycol (PEG)-induced osmotic stress to simulate drought conditions. Next, we utilized the DIA-MS method to perform a comprehensive proteomic profiling of Tibetan hulless barley under osmotic stress. Time-course and pairwise comparison analyses of all samples at each time point were conducted with the protein abundance. Then, we examined the physiological or biological processes of differentially abundant proteins (DAPs) in each comparison. Our analysis revealed several important stress-responsive genes and functional modules, such as hormone metabolism, including ethylene, salicylic acid and cytokinin; cell wall or cell architecture associated with membrane stability; reactive oxygen species (ROS)-scavenging enzymes; and ABA-induced signalling genes. We further selected some known drought stress-responsive genes from public databases or articles and explored their distribution curves at each time point. Finally, using a machine-learning approach, we constructed a gene regulatory network and revealed several key regulatory elements associated with osmotic stress tolerance.

Data Description

Plant materials and cultivation

Two Tibetan hulless barley inbred lines, drought-sensitive (DQ) and drought-resistant (XL), were used for our experiments. Specifically, we acquired the DQ cultivar from the Institute of Agricultural Research, Tibet Academy of Agricultural and Animal Husbandry Sciences and the XL cultivar from The Tibet Autonomous Region Xigaze Agricultural Science Research Institute. Seeds of the two cultivars were sown with nutritional soil and maintained in plant growth incubators at 25°C, 2000 µmol.m^{-2}.s^{-1}. In the 2-3 leaf stage, seedlings were removed from the tray and cultivated in half-strength Hoagland's nutrient solution [26]. Specifically, a polyethylene glycol (PEG) solution with a concentration of 21% was used to simulate osmotic stress caused by drought. For each cultivar, half of the plants were replaced with PEG6000 embedding

medium after seven days of growth. Next, fresh leaves from two cultivars in the control group (CK) and stress treatment group (ST) were sampled at 1, 4, 8, 12, 24, and 48 h, respectively. For the individual plants with specific sampling time points and treatments, three replicates were collected and then kept at -80°C until they were analysed.

Protein extraction and digestion

For each plant tissue sample, a 1-g subsample was weighed and homogenized by grinding in liquid nitrogen. The powdered samples were moved to 50 cm^3 tubes with 25 cm^3 precooled acetone (-20 °C) containing 10% (v/v) trichloroacetic acid (TCA) and 10 mM dithiothreitol (DTT). After thorough mixing, the homogenate was precipitated overnight at -20 °C and then centrifuged (20,000×g, 4 °C) for 30 min. The pellet was then washed twice with 20 cm^3 chilled acetone (-20 °C) and left at -20 °C for 30 min followed by centrifugation (20,000×g, 4 °C) for 30 min. The precipitate was dissolved with lysis buffer (4% SDS, 100 mM Tris-HCl, 10 mM DTT, pH 8.0) and sonicated for 5 min at 60 W (5 s sonication followed by 10 s break) followed by 30 min centrifugation (20,000×g, 20 °C). The supernatant was collected, and the protein concentration in the lysate was estimated using a bicinchoninic acid (BCA) protein assay kit (Beyotime Institute of Biotechnology, China).

Protein digestion was conducted using the FASP (filter-aided sample preparation) procedure [27]. In brief, protein extracts in an ultrafiltration filtrate tube (30 kDa cut-off, Sartorius, Germany) were mixed with 200 mm^3 UA buffer (8 M urea, 150 mM Tris-HCl, pH 8.0) and centrifuged at 14,000 g at 20°C for 40 min. Samples were washed twice by adding 200 mm^3 UA to the filter unit and centrifuged at 14,000 g at 20 °C for 40 min. After discarding the flow-through from the collection tube, 100 mm^3 IAM solution (10 mM IAM in UA buffer) was added into the filter tube and incubated for 30 min. Samples were washed twice with 100 mm^3 of UA to the filter unit. After centrifuging with 14,000 g at 20°C for 40 min, 100 mm^3 of ABC (0.05 M NH_4HCO_3 in water) was added into the filter unit and centrifuged at 14,000 g. The protein suspension in the filtrate tube was subjected to enzyme digestion with 40 mm^3 ABC with trypsin (Promega, USA) and incubated for 18 h at 37°C. The filtrate was used for LC-MS analysis after centrifugation at 14,000 g for 10 min. The QC mixture was formed by pooling equal amounts of peptides from all individuals, which was used to evaluate the reproducibility of the quantitative LC-MS analysis.

Peptide fractionation by high-pH RP

Digested peptides were separated on an LC-20AB HPLC system (Shimadzu, Kyoto, Japan) with a high-pH reversed-phase (high-pH RP) column (Phenomenon, Torrance, CA). Peptides were eluted at a flow rate of 0.8 cm^3/min. Buffer A consisted of 10 mM ammonium acetate (pH 10.0), and buffer B consisted of 10 mM ammonium acetate and

90% v/v acetonitrile (pH 10.0). The following gradient was applied to perform separation: 100% buffer A for 40 min, 0-5% buffer B for 3 min, 5-35% buffer B for 30 min, 35-70% buffer B for 10 min, 70-75% buffer B for 10 min, 75-100% buffer B for 7 min, 100% buffer B for 15 min and, finally, 100% buffer A for 15 min. The elution process was monitored by measuring absorbance at 214 nm, and fractions were collected every 75 s. Finally, collected fractions (approximately 40) were combined into 12 pools. Each fraction was concentrated via vacuum centrifugation and reconstituted in 40 mm^3 of 0.1% v/v formic acid. All samples were stored at -20 °C until further analysis.

LC mass spectrometry analysis

Peptides were separated with a Dionex UltiMate 3000 RSLCnano system with an Acclaim PepMap C18 (3 µm, 100 Å, 75 µm x 50 cm) and emitted into a Thermo Q-Exactive HF tandem mass spectrometer. Solvent A was 0.1% formic acid in water, while solvent B was 0.1% formic acid in 98% acetonitrile. For each injection, 3 mm^3 (approximately 3 µg) was loaded and eluted using a 90-minute gradient from 5 to 35% B followed by a 40 min washing gradient. Data were acquired using either data-dependent acquisition (DDA) or data-independent acquisition (DIA).

For library generation, the Thermo Q-Exactive HF was set to positive mode in a top-20 configuration to acquire data in DDA mode. Precursor spectra (375-1400 m/z) were collected at 120,000 resolution to reach an AGC target of 3e6. The maximum injection time was set to 20 ms. Fragment spectra were collected at 30,000 resolution to reach an AGC target of 1e5 with a maximum injection time of 60 ms. The isolation width was set to 1.6 m/z with a normalized collision energy of 25. Only precursors charged between +2 and +6 that achieved a minimum AGC of 2e3 were acquired. Dynamic exclusion was set to 30 s and to exclude all isotopes in a cluster.

For quantitative samples, the Thermo Q-Exactive HF was configured to acquire 55 × 16 m/z DIA spectra (16 m/z precursor isolation windows at 30,000 resolution, AGC target 1e6, maximum injection time 55 ms). Precursor spectra (400-1250 m/z) were collected at 120,000 resolution to reach an AGC target of 3e6. The maximum injection time was set to 50 ms. To evaluate the reproducibility of the LC-MS system during the whole DIA acquisition, the samples and QCs were analysed following this scenario: one QC injection followed by 10 experimental samples until all were measured.

Library generation and quantitative data analyses

MaxQuant (version 1.6.2.6; RRID:SCR_014485) software [28, 29] was used to analyse the DDA MS/MS data with the following settings: enzyme: trypsin/P; maximum missed

cleavages: 2; fixed modification: carbamidomethyl (C); variable modifications: oxidation (M) and acetyl (protein N-term); precursor mass tolerance: 20 ppm; fragment mass tolerance: 0.05 Da; second peptide search was enabled. All other parameters are in default. The MS/MS data were searched against the *Hordeum vulgare* (barley) protein sequences, which were downloaded from the UniProt database (version 2018.7, 210,953 entries), appended with the Biognosys iRT peptide sequences. The FDR threshold was set as 1% at both PSM (peptide spectrum match) and protein levels. Subsequently, the MaxQuant search result was imported into Spectronaut Pulsar (12.0.20491.4, Biognosys, Schlieren, Switzerland) to generate a spectra library with the default settings.

Spectronaut Pulsar was used to analyse the DIA data with the spectra library based on DDA MS/MS data. Local regression normalization was used for protein quantification normalization. Dynamic MS1 and MS2 mass tolerance strategies were applied for data extraction with a correction factor of 1. A dynamic XIC RT extraction window with a local nonlinear iRT strategy was chosen for calibration. Interference correction was enabled to automatically remove fragments that interfere with other ions across several runs. The decoy method in the feature identification was configured as “mutated” with a decoy limit strategy of “dynamic” and library size fraction of 0.1. The results were filtered by 1% FDR, and only those protein groups that passed these filter criteria were used in downstream analysis. The DIA raw data and the corresponding results were deposited into the iProX database [30].

Bioinformatic data analysis

Statistical analysis and graphical display were performed with R language environment (version 3.5.0). Hierarchical clustering was performed using the R package pheatmap [31]. Principal component analysis (PCA) was performed using the FactoMineR package [32]. A t-test was used for statistical differential analysis, and a cut-off of p-value <= 0.05 and fold change >= 2 was used to select statistically differentially abundant proteins. Hypergeometric-based enrichment analysis with KEGG (KEGG, RRID:SCR_012773) [33], Gene Ontology [34, 35] and MapMan (MapMan, RRID:SCR_003543) [36-38] was performed to annotate protein sequences individually. The abundance curve of the target gene was depicted with protein abundance. A LOESS method implemented in the R environment was used to fit the smooth curves by a set of data points [39]. For network analysis, the target genes of plant transcription factors (TFs) and protein kinases (PKs) are classified by the iTAK program [40]. The Arboreto computational framework integrated with the GRNBoost2 [41] algorithm was used to reconstruct relevant regulatory relationships in each ecotype. The igraph package was used to visualize networks [42].

Analyses

Quality control analysis of the barley proteome

In this study, we identified a total of 6921 proteins with a 1% FDR in all samples, with a maximum of 6313 proteins being quantified in a single non-QC sample (i.e., the FDR). treatment group of XL at 48 h, replicate #II, Figure S1). The MS platform was stable and repeatable as evaluated by quality control runs during the entire data-collecting period. The coefficient of variation (CV), reflecting the magnitude of variability in protein abundance, accounted for an average of 20% in each sample (Figure S2a). The relationship between CV and the log area is illustrated in Figure S2b, and proteins were assorted into 12 intervals according to their log area values in descending order. The results revealed that proteins with higher intensity always showed smaller CVs, which is in accordance with a previous study [43]. The hierarchical clustering-based heatmap and principal component analysis (PCA) based on quantified protein abundances in each sample were used for further quality control, as illustrated in Figure S2c and Figure S2d. The nine QC samples were clustered together, which indicates that the MS platform was stable and the quality of DIA data was high.

Pairwise differential abundance analysis

To explore proteins associated with osmotic stress, two types of analysis were performed. According to the time point experimental design of XL and DQ, all cultivars were divided into 10 comparison groups (Figure 1). Each group consisted of a treatment-control pair, and the relative fold change of protein was calculated for each paired group. The statistical significance of the observed fold change was determined by paired t test for all the DAPs, and the threshold of p-value ≤ 0.05 and fold change$\geq$2 was used. As shown in Figure 2, the DAP numbers varied significantly at different time points together with a relatively low number of common changes (yellow area), indicating highly diverse dynamics of protein expression regulation in XL and DQ. Compared with downregulated proteins, more upregulated proteins were found at 4 h and 8 h.

To explore the biological processes in each DAP group, we conducted hypergeometric-based enrichment analysis based on MapMan and Gene Ontology (GO) databases. The threshold of an adjusted p-value <= 0.05 was used to define significantly enriched annotation categories. To highlight the key function terms, we manually reviewed the biological function terms in Supplementary Tables S1, S2 and S3. Unique osmotic-related entries of DL or XL were selected and labelled beside the related bars with short abbreviations. In particular, “cytokinin synthesis degradation”, “UDP glucosyl and glucoronyl transferases (UGTs)” and “leaf senescence” were the dominant responses in

DAPs of the DQ cultivar, whereas “MAP kinases” and “ethylene synthesis degradation” were two key terms in DAPs of the XL cultivar. More details can be obtained through the table below the diagram in Figure 2.

To further explore the biological functions of DAPs, we divided the upregulated and downregulated genes into independent gene sets and reannotated them separately. Based on the Mapman annotations, we used a hierarchical heatmap to represent the relationship between time stages and relevant annotated entries. As shown in Figure 3, the colour scale was graded to reflect the enrichment scores (log2-transformed FDR). Among these functional terms, two hormone metabolism terms, “abscisic acid induced-regulated-responsive-activate” and “ethylene.synthesis-degradation”, were significantly enriched in the XL upregulated gene set at 8 h. Another hormone metabolism term, “salicylic acid synthesis-degradation”, was upregulated in the sensitive cultivar (DQ) at 4 h. Moreover, some proteins involved in cell wall formation were upregulated at 24 h in the XL cultivar, and some proteins involved in wax biosynthesis were also upregulated at 8 h in the DQ cultivar. Interestingly, cytochrome P450, an important protein-coding gene family involved in growth and drought stress responses [44], was upregulated in DQ but downregulated in the XL cultivar. A similar result from Wendelboe-Nelson et al. showed that cytochrome P450 was downregulated in tolerant cultivars [45].

We then conducted gene ontology (GO) enrichment analysis. The assigned functions of these genes covered a broad range of GO categories (Figure S3). Specifically, in the biological process category, sulfate assimilation, cellular response to oxidative stress and chitin catabolic process were the major functional terms for the osmotic stress response in the DQ cultivar. In contrast, the ethylene biosynthetic process, toxin catabolic process and photosynthesis of light harvesting in photosystem I may be involved in osmotic stress tolerance in the XL cultivar. For the genes enriched in categories related to cellular components, several photosynthesis terms of photosystem I and photosystem II were upregulated in several stages of XL. For the molecular function category, many potential osmotic stress-induced genes were classified into a series of redox-related functional items, including glutathione peroxidase activity, glutathione transferase activity, peroxiredoxin activity, pigment binding, and chlorophyll binding. Additionally, we identified some chitin-binding proteins related to the pathogenesis-related gene family, which may contribute to the defence response of plants under osmotic stress [46]. Finally, based on the MapMan and GO annotations of pairwise analysis, we generated Tables S7 and S8 to summarize the differences in response to osmotic stress between DQ and XL.

Time-course differential abundance analysis

To investigate the impact of stress degree differences on protein abundance in the consecutive developmental stages over time, stepwise comparisons (e.g., T4 vs. T1, T8

vs. T4, T24 vs. T8, T48 vs. T24, and T48 vs. T1) were performed in the treatment and control groups of DQ and XL samples separately (Figure 1 and Figure 4). DAPs were selected based on the threshold of protein abundance fold changes ≥ 2 and p-value ≤ 0.05. To explore the relationship of significant DAPs in different treatments of XL and DQ, we generated five Venn diagrams. Next, we carried out the functional characterization of unique DAPs in the DQ and XL treatments individually. Several potential osmotic stress-induced entries unique to DQ or XL treatments are manually selected and labelled beside the diagram with abbreviations. The complete annotation list can be obtained in upplementary Tables S4, S5 and S6. For instance, “cytokinin synthesis degradation”, “UDP glucosyl and glucoronyl transferases” and “wax-related” were likely to be an exclusive response in the DQ cultivar, whereas “GDSL-motif lipase”, “DUF26 kinase” and “plasma membrane intrinsic protein (PIP)” were three main functional terms in the XL cultivar.

Core genes in the plant defence response

To discover potential osmotic stress-induced genes, we explored the abundances of some well-known genes related to the plant defence response, such as ARF, KAT, MAPK, PR10 SnRK2 and WRKY. With BLAST alignment, we obtained the UniProt accession that is relevant to the candidate genes. Next, we examined the individual abundance levels of these genes and depicted the abundance profiles in Figure 5. Through closer examination of these genes, we found that MAPK (M0V3Q0) and PR10 (Q84QC7) exhibited higher abundance in the treatment group over all time points of XL and DQ, indicating that the two genes might play important roles in plant defence against osmotic stress. Additionally, SnRK2 (M0XX02) and WRKY (B2KJ55) also showed a similar trend, which were upregulated at 4 h and 8 h of DQ but downregulated at 24 h of XL.

To further investigate the potential osmotic stress tolerance mechanisms, we collected manually curated genes involved in the drought stress response from a public database: DroughtDB [47]. We selected the best hit for each subject sequence with the threshold of evalue >= 0.00001 and identity >= 80% using Blast. Most of the genes were aligned to the genes identified in this study. As shown in Figure S4, none of these genes showed significant abundance changes between the DQ and XL cultivars. In addition, we selected four water deprivation-related gene ontology (GO) terms, including cellular response to water deprivation, response to water deprivation, response to desiccation and positive regulation of response to water deprivation, and we collected the relevant protein sequences from *Oryza sativa* (rice), as annotated by UniProt. We performed a similar analysis, and as shown in Figure S5, compared with the control group, most of the genes in the treatment group showed relatively higher abundance levels in both the DQ and XL cultivars. This phenomenon indicates that water deprivation is a vital regulatory mechanism for both XL and DQ under osmotic stress.

Gene regulated network

Considering that genes could produce complex dynamic systems or gene regulatory networks to defend against osmotic stress during plant growth, we explored the coexpression patterns and potential regulatory associations that were represented in gene regulatory networks (GRNs). Specifically, 21 potential stress-responding genes were chosen as the candidate target gene set (Figure 6). Among these genes, 8 TFs are in the families of Alfin-like, WRKY, MYB-related, bZIP, GRF, bHLH and B3-ARF; 12 genes belong to important osmotic stress responsive genes, including ARF, MAPK, SnRK2 and PR10; and one gene encodes AMP-activated protein kinase (AMPK). Then, arboreto takes this target-gene abundance matrix as inputs and produces reliable interaction predictions. Based on the abundances of a set of candidate genes, we constructed a partial GRN with regulatory associations using the identified stress-responding genes for each ecotype (see Methods). As shown in Figure 6, this result revealed that bHLH, GRF and PR10 had more connections in DQ than in XL, indicating that these two genes probably play important roles in the osmotic stress response process of the DQ cultivar. In addition, MAPK and SnRK2 showed more connections in XL than in DL. Remarkably, AMPK was the hub gene with the highest connection number in both the XL and DQ cultivars.

Discussion

Drought is one of the most acute environmental stresses that directly affects agricultural productivity. In this study, we first utilized DIA-MS-based proteomics technology to quantify proteins in different samples and explored essential DAPs in hulless barley over multiple time points under two cultivars. Using two different comparison strategies, time-course and pairwise, we conducted a comprehensive analysis to explore protein-level changes in response to osmotic stresses.

We detected some essential biological function terms related to osmotic stress regulation in the DQ cultivar. Specifically, cytokinins are a class of growth-promoting hormones regulating various developmental processes, including cell division and senescence [48]. Previous studies revealed that reduced cytokinin levels could improve osmotic stress tolerance by suppressing growth and reducing stomatal density [49, 50]. Additionally, the "leaf senescence" entry at 24 h presents an accelerated leaf senescence of the DQ cultivar and implies that the DQ cultivar might be more sensitive to osmotic stress. Moreover, UDP-glucuronosyl/UDP-glucosyl transferases, pronounced changes at 4 h of DQ, are a superfamily of enzymes that catalyse glucuronidation reactions [51], and they were found to enhance plant tolerance under a series of adverse environmental factors, including low temperatures, salinity and drought [52]. Furthermore, we found that the function term "salicylic acid synthesis-degradation" was only upregulated in the sensitive cultivar (DQ). Salicylic acid (SA) is a vital phytohormone required for

systemic acquired resistance (SAR) in plants and plays a vital role in the defence against pathogens [53]. It has also been reported that SA could ameliorate oxidative stress and enhance plant tolerance to abiotic [54]stress. Our results postulate that the degradation of SA tends to make the DQ cultivar more susceptible under osmotic stress.

The functional terms from ABA-dependent and ABA-independent signalling pathways exhibited dominant abundance changes in the XL cultivar. Several studies have proven that ABA plays a key role in regulating the adaptive response of plants under diverse stress conditions [55, 56]. Meanwhile, the plant hormone ethylene is well known to play an essential role in plant growth, development and osmotic stress resistance. In particular, lower ethylene levels would lead to higher osmotic stress tolerance. Shi et al. also indicated that a reduced sensitivity to ethylene by CRISPR-Cas9 technology would enhance cell elongation and division, thereby increasing grain yield under osmotic stress conditions [57]. Moreover, glycine-rich RNA-binding proteins (GRPs) are known to transport and regulate RNA processing. A study from Kim et al. suggested that GRPs influence the opening and closing of the stomata [58]. Vítámvás et al. reported that drought treatments profoundly affected glycine-rich RNA-binding protein abundances [59]. In this study, glycine-rich RNA-binding protein abundances were found to increase only in the XL cultivar, indicating that these proteins may improve osmotic stress tolerance in hulless barley.

In the biological process shown in Figure S3, some upregulated proteins of the XL cultivar were enriched in toxin catabolic processes from 4 h to 8 h. This catabolic process may detoxify the ROS produced during osmotic stress treatments, and it is one of the potential mechanisms that makes XL more tolerant to environmental stress than DQ. Moreover, flavonoids are thought to be one of the key compounds that protect plants against various biotic and abiotic stresses by inhibiting ROS formation [60]. However, the enzymes involved in flavonoid metabolism were downregulated in the early stage of the XL cultivar. Interestingly, a similar phenomenon can be observed in Vítámvás et al.'s report [59].

Ascorbate peroxidase (APX) and glutathione peroxidase (GPX) are two plant antioxidant enzymes that can remove H_2O_2 and prevent potential cellular damage [61, 62]. Interestingly, ascorbate peroxidase activity was found to increase from 24 h to 48 h of DQ and 48 h of XL (Figure 3S). The increased protein abundance of APX was also observed in both tested barley lines by Chmielewska et al. [63]. The findings of these researchers are in accordance with our detections, and we postulated that DQ with a quicker response is more susceptible to osmotic stress than the XL cultivar. In addition, Vítámvás et al. revealed that GPX had a continuous and significant increase with decreasing soil water capacity [59]. A similar phenomenon was observed in our results with increasing glutathione peroxidase activity at 4 h in the XL cultivar, indicating that GPX has the capacity to enhance tolerance against abiotic stress. Moreover, peroxiredoxins (Prxs) are a highly conserved family of antioxidant enzymes that catalyse the peroxide reduction of H_2O_2. Ghabooli et al. showed that the protein levels

of APXs and Prxs were upregulated in barley plants under drought treatment [64]. Similarly, we found that peroxiredoxin activity was significantly increased at 8 h in the XL cultivar (Figure 3 or Figure S3). Furthermore, glutathione transferase (GST), which is believed to conjugate xenobiotics with glutathione [59], was also upregulated at 8 h of XL. These results may reveal that the XL cultivar has more ROS scavenging mechanisms to enhance osmotic stress tolerance than the DQ cultivar.

We also identified several genes relevant to cell wall construction. Since the cell wall is the first line to defend against abiotic stress, many proteins that are involved in cell wall strengthening or cellular membrane stabilization will be significantly regulated under osmotic stress [65]. Interestingly, a variety of transport-related proteins were downregulated under osmotic stress in the first time point of the DQ cultivar (Figure S3), indicating that DQ is more sensitive than the XL cultivar and promotes osmotic tolerance through suppression of several transport activities in the early stage of development.

In the time-course comparison, we identified genes related to cytokinin degradation and UGT in the DQ cultivar treatment group. Moreover, we found some DAPs from the DQ cultivar that can produce a secondary metabolite (wax) in time-course comparison analysis, as shown in Figure 4. The increased accumulation of cuticular wax under osmotic stress conditions can improve tolerance and reduce water loss [66]. In addition, three manually reviewed entries were specific in the treatment group of the XL cultivar. Of these entries, Hong et al. demonstrated that GDSL-type lipase can activate susceptibility to disease and tolerance to abiotic stress [67]. Miyakawa et al. demonstrated that a plant-specific cysteine-rich motif (DUF26) may be widely involved in plant-specific responses to biotic and abiotic stresses [68]. Lu et al. showed that changes in the gene expression of some plasma membrane intrinsic proteins (PIPs) can also promote osmotic stress tolerance [69]. Overall, the analyses carried out in this study have confirmed findings reported in previous studies and provided additional evidence of abiotic tolerance in resistant compared to susceptible cultivars.

To investigate the expression status of several well-known osmotic stress genes, we found that four proteins (MAPK, PR10, SnRK2 and WRKY) had significant changes in protein abundance between the control and treatment groups. Of these, the mitogen-activated protein kinase (MAPK) cascade is one of the major signalling pathways involved in the abiotic stress response in plants [70]. It is evolutionarily conserved among eukaryotic organisms and can transduce extracellular signals to the nucleus under abiotic stress [71, 72]. The PR10 gene has been confirmed to be overexpressed in rice and to enhance drought and salt stress tolerance [73]. Additionally, WRKY transcription factors were thought to participate in the regulation of water stress and drought responses [74]. Taken together, these results demonstrated that these genes were potential candidate genes for agricultural application to protect crops against biotic and abiotic stresses.

In the gene regulatory network, we found a few genes centrally positioned in the network, suggesting that these genes may have a dominant role/regulation in Tibetan hulless barley. Specifically, growth regulating factors (GRFs) and the basic helix-loop-helix (bHLH) protein family are plant-specific transcription factors that are involved in diverse biological or physiological processes, such as growth, hormone responses and stress [75, 76]. AMPK, known to be responsible for the maintenance of ATP balance during energy metabolism [77], occupies the central position in both networks, indicating that it is likely to be a core regulatory component in the osmotic stress resistance network. Moreover, SnRK2, a serine/threonine kinase specific in plants involved in plant responses to abiotic stresses and abscisic acid (ABA)-dependent plant development [78], showed higher abundance in XL than in DQ. Furthermore, we also found that MAPK-related genes showed more connections in the XL cultivar. Previous studies suggested that the mitogen-activated protein kinase could be activated by the ABA core signalling module through transcriptional regulation [11]. Thus, we could infer that the ABA-induced pathway might have a stronger impact on XL than the DQ cultivar.

In summary, this proteomic study provides a valuable resource to explore stress-responsive proteins that can help us understand the underlying regulatory mechanisms in Tibetan hulless barley. Furthermore, these data will be valuable to plant biologists who are interested in exploring signalling mechanisms to osmotic/drought stress, thereby helping to promote drought stress tolerance in crops.

Availability of supporting data and materials

All of the MS raw data (DIA and DDA) have been deposited to the ProteomeXchange Consortium via the iProX partner repository[30] with the dataset identifier PXD015597. All supporting data and materials are available in the *GigaScience* GigaDB database [79].

Additional files

Supplementary Figure S1. Numbers of proteins detected in each sample.

Supplementary Figure S2a. Distribution of protein abundance variability. The CV value of each protein was calculated by R environment with formula as “sd(biological replicates)/mean(biological replicates))”.

Supplementary Figure S2b. Relationship between CV and protein abundance (log2 transformed). The CV value drops with increasing protein abundance.

Supplementary Figure S2c. Heatmap of protein abundances between different samples. The hierarchical clustering is performed using neighbor joining algorithm with a Euclidean distance similarity measurement of the log2 of the protein abundance.

Supplementary Figure S2d. Principal component analysis (PCA) score plot for proteins in the DQ and XL cultivars between the treatment and control groups. Each point represents a sample.

Supplementary Figure S3. Gene ontology enriched heatmap for DAPs in pairwise comparison. Similar to Figure 3, but with gene ontology instead of MapMan database. The left panel shows the annotation of upregulated proteins and the right panel shows the annotation of downregulated proteins. Row names are the samples from five time points in the DQ and XL cultivars. Column names are the enriched items from three aspects of gene ontology database (biological process: BP; cellular component: CC; and molecular function: MF). The legend shows the colour scaling with FDR values. See Supplementary Table S1 for the entire list of the GO terms.

Supplementary Figure S4. Protein abundance changes of osmotic stress-induced genes from the DroughtDB database. Similar to Figure 5, the labels in the left panel are the description of the related genes. In particular, the identifier in the left of tilde (~) symbol is the UniProt accession of *Hordeum vulgare*, the identifier in the right of tilde is the gene symbol from DroughtDB, and the description under the tilde is the osmotic stress-related functional annotation.

Supplementary Figure S5. Protein abundance changes of osmotic stress-induced genes from gene ontology database. Similar to Figure 5, the labels in the first left panel are the UniProt accessions of *Hordeum vulgare*, and the labels in the second left panel are the functional description from gene ontology with BLAST.

Supplementary Table S1. Gene ontology enrichment list of DAPs in pairwise comparison.

Supplementary Table S2. MapMan enrichment list of DAPs in pairwise comparison.

Supplementary Table S3. KEGG pathway enrichment list of DAPs in pairwise comparison.

Supplementary Table S4. Gene ontology enrichment list of DAPs in time-course comparison.

Supplementary Table S5. MapMan enrichment list of DAPs in time-course comparison.

Supplementary Table S6. KEGG pathway enrichment list of DAPs in time-course

comparison.

Supplementary Table S7. MapMan annotation differences in response to osmotic stress between DQ and XL. Green represents enriched terms in downregulated proteins. Red represents enriched terms in upregulated proteins. And NA represents the functional entry that is not statistically significant or not available.

Supplementary Table S8. Gene ontology annotation differences in response to osmotic stress between DQ and XL. Green represents enriched GO terms in downregulated proteins. Red represents enriched GO terms in upregulated proteins. And NA represents the GO entry that is not statistically significant or not available.

Abbreviations

DIA: data-independent acquisition; DDA: data-dependent acquisition; MS: mass spectrometry; ABA: abscisic acid; DAP: differentially abundant protein; CK: control group; ST: treatment group; PSM: peptide spectrum match; PCA: Principal component analysis; CV: coefficient of variation; GRN: Gene Regulatory Networks; BLAST: Basic Local Alignment Search Tool; GO: Gene Ontology; KEGG: Kyoto Encyclopedia of Genes and Genomes; SA: Salicylic acid; PEG: polyethylene glycol; APX: ascorbate peroxidase; GPX: glutathione peroxidase: Prxs: peroxiredoxins

Competing interests

The authors declare that they have no competing interests.

Funding

This work was supported by the following funding sources: The Financial Special Fund (2017CZZX001, XZNKY-2018-C-021).

Authors' contributions

H.Y. and Y.W. conceived the idea of the work and designed the research; Z.S., Q.X. and

S.X. produced and analyzed the data; Q.X., Y.W., D.J. and Z.S. managed the samples and the data; H.Y., Y.W. and Q.X. wrote the paper; X.Z. and Y.W. revised the manuscript. All authors read and approved the final manuscript.

References

1. Seki M, Kamei A, Yamaguchi-Shinozaki K and Shinozaki K. Molecular responses to drought, salinity and frost: common and different paths for plant protection. Curr Opin Biotechnol. 2003;14 2:194-9.
2. Pennisi E. Plant genetics. The blue revolution, drop by drop, gene by gene. Science. 2008;320 5873:171-3. doi:10.1126/science.320.5873.171.
3. Deeba F, Pandey AK, Ranjan S, Mishra A, Singh R, Sharma YK, et al. Physiological and proteomic responses of cotton (Gossypium herbaceum L.) to drought stress. Plant Physiol Biochem. 2012;53:6-18. doi:10.1016/j.plaphy.2012.01.002.
4. Barnabas B, Jager K and Feher A. The effect of drought and heat stress on reproductive processes in cereals. Plant Cell Environ. 2008;31 1:11-38. doi:10.1111/j.1365-3040.2007.01727.x.
5. Lesk C, Rowhani P and Ramankutty N. Influence of extreme weather disasters on global crop production. Nature. 2016;529 7584:84-7. doi:10.1038/nature16467.
6. CRAMER GR. Abiotic stress and plant responses from the whole vine to the genes. Australian Journal of Grape and Wine Research. 2010;16 s1:86-93. doi:10.1111/j.1755-0238.2009.00058.x.
7. Skirycz A and Inzé D. More from less: plant growth under limited water. Current Opinion in Biotechnology. 2010;21 2:197-203. doi:https://doi.org/10.1016/j.copbio.2010.03.002.
8. Cramer GR, Urano K, Delrot S, Pezzotti M and Shinozaki K. Effects of abiotic stress on plants: a systems biology perspective. BMC Plant Biology. 2011;11 1:163. doi:10.1186/1471-2229-11-163.
9. Zlatev Z and Lidon F. An overview on drought induced changes in plant growth, water relations and photosynthesis. 2012.
10. Anjum S, Ashraf U, Tanveer M, Khan I, Hussain S, Shahzad B, et al. Drought Induced Changes in Growth, Osmolyte Accumulation and Antioxidant Metabolism of Three Maize Hybrids. 2017.
11. de Zelicourt A, Colcombet J and Hirt H. The Role of MAPK Modules and ABA during Abiotic Stress Signaling. Trends Plant Sci. 2016;21 8:677-85. doi:10.1016/j.tplants.2016.04.004.
12. Thomashow MF. PLANT COLD ACCLIMATION: Freezing Tolerance Genes and Regulatory Mechanisms. Annual Review of Plant Physiology and Plant Molecular Biology. 1999;50 1:571-99. doi:10.1146/annurev.arplant.50.1.571.
13. Nambara E and Marion-Poll A. ABSCISIC ACID BIOSYNTHESIS AND CATABOLISM. Annual Review of Plant Biology. 2005;56 1:165-85. doi:10.1146/annurev.arplant.56.032604.144046.
14. Fujita M, Fujita Y, Maruyama K, Seki M, Hiratsu K, Ohme-Takagi M, et al. A dehydration-induced NAC protein, RD26, is involved in a novel ABA-dependent stress-signaling pathway.

Plant J. 2004;39 6:863-76. doi:10.1111/j.1365-313X.2004.02171.x.
15. Mukhopadhyay A, Vij S and Tyagi AK. Overexpression of a zinc-finger protein gene from rice confers tolerance to cold, dehydration, and salt stress in transgenic tobacco. Proceedings of the National Academy of Sciences of the United States of America. 2004;101 16:6309. doi:10.1073/pnas.0401572101.
16. Agarwal PK, Agarwal P, Reddy MK and Sopory SK. Role of DREB transcription factors in abiotic and biotic stress tolerance in plants. Plant Cell Rep. 2006;25 12:1263-74. doi:10.1007/s00299-006-0204-8.
17. Zeng X, Long H, Wang Z, Zhao S, Tang Y, Huang Z, et al. The draft genome of Tibetan hulless barley reveals adaptive patterns to the high stressful Tibetan Plateau. Proceedings of the National Academy of Sciences. 2015:201423628. doi:10.1073/pnas.1423628112.
18. Wei Z, Zeng X, Qin C, Wang Y, Bai L, Xu Q, et al. Comparative Transcriptome Analysis Revealed Genes Commonly Responsive to Varied Nitrate Stress in Leaves of Tibetan Hulless Barley. Frontiers in Plant Science. 2016;7 1067 doi:10.3389/fpls.2016.01067.
19. Zeng X, Bai L, Wei Z, Yuan H, Wang Y, Xu Q, et al. Transcriptome analysis revealed the drought-responsive genes in Tibetan hulless barley. BMC Genomics. 2016;17 1:386. doi:10.1186/s12864-016-2685-3.
20. Fukao Y. Discordance between protein and transcript levels detected by selected reaction monitoring. Plant Signaling & Behavior. 2015;10 5:e1017697. doi:10.1080/15592324.2015.1017697.
21. Gygi SP, Rochon Y, Franza BR and Aebersold R. Correlation between Protein and mRNA Abundance in Yeast. Molecular and Cellular Biology. 1999;19 3:1720-30. doi:10.1128/mcb.19.3.1720.
22. Lu P, Vogel C, Wang R, Yao X and Marcotte EM. Absolute protein expression profiling estimates the relative contributions of transcriptional and translational regulation. Nature Biotechnology. 2006;25:117. doi:10.1038/nbt1270
https://www.nature.com/articles/nbt1270#supplementary-information.
23. Doerr A. DIA mass spectrometry. Nature Methods. 2014;12:35. doi:10.1038/nmeth.3234.
24. Law KP and Lim YP. Recent advances in mass spectrometry: data independent analysis and hyper reaction monitoring. Expert Review of Proteomics. 2013;10 6:551-66. doi:10.1586/14789450.2013.858022.
25. Bruderer R, Bernhardt OM, Gandhi T, Miladinovic SM, Cheng L-Y, Messner S, et al. Extending the limits of quantitative proteome profiling with data-independent acquisition and application to acetaminophen treated 3D liver microtissues. Molecular & Cellular Proteomics. 2015:mcp.M114.044305. doi:10.1074/mcp.M114.044305.
26. Hoagland DR and Arnon DI. The Water-Culture Method for Growing Plants Without Soil. 1937.
27. Wisniewski JR, Zougman A, Nagaraj N and Mann M. Universal sample preparation method for proteome analysis. Nat Methods. 2009;6 5:359-62. doi:10.1038/nmeth.1322.
28. Tyanova S, Temu T and Cox J. The MaxQuant computational platform for mass spectrometry-based shotgun proteomics. Nature Protocols. 2016;11:2301. doi:10.1038/nprot.2016.136.
29. Cox J and Mann M. MaxQuant enables high peptide identification rates, individualized p.p.b.-range mass accuracies and proteome-wide protein quantification. Nature Biotechnology. 2008;26 12:1367-72. doi:10.1038/nbt.1511.

30. Ma J, Chen T, Wu S, Yang C, Bai M, Shu K, et al. iProX: an integrated proteome resource. Nucleic Acids Res. 2019;47 D1:D1211-D7. doi:10.1093/nar/gky869.
31. Kolde R. pheatmap: Pretty Heatmaps. R package version 1.0.12. https://CRAN.R-project.org/package=pheatmap.
32. Lê S, Josse J and Husson F. FactoMineR: An R package for multivariate analysis. Journal of Statistical Software. 2008;25 1:1-18.
33. Kanehisa M and Goto S. KEGG: kyoto encyclopedia of genes and genomes. Nucleic Acids Res. 2000;28 1:27-30. doi:10.1093/nar/28.1.27.
34. The Gene Ontology C. The Gene Ontology Resource: 20 years and still GOing strong. Nucleic Acids Res. 2019;47 D1:D330-D8. doi:10.1093/nar/gky1055.
35. Ashburner M, Ball CA, Blake JA, Botstein D, Butler H, Cherry JM, et al. Gene ontology: tool for the unification of biology. The Gene Ontology Consortium. Nat Genet. 2000;25 1:25-9. doi:10.1038/75556.
36. Usadel B, Poree F, Nagel A, Lohse M, Czedik-Eysenberg A and Stitt M. A guide to using MapMan to visualize and compare Omics data in plants: a case study in the crop species, Maize. Plant Cell Environ. 2009;32 9:1211-29. doi:10.1111/j.1365-3040.2009.01978.x.
37. Thimm O, Blasing O, Gibon Y, Nagel A, Meyer S, Kruger P, et al. MAPMAN: a user-driven tool to display genomics data sets onto diagrams of metabolic pathways and other biological processes. Plant J. 2004;37 6:914-39.
38. Lohse M, Nagel A, Herter T, May P, Schroda M, Zrenner R, et al. Mercator: a fast and simple web server for genome scale functional annotation of plant sequence data. Plant Cell Environ. 2014;37 5:1250-8. doi:10.1111/pce.12231.
39. Cleveland WS, Devlin SJ and Grosse E. Regression by local fitting: Methods, properties, and computational algorithms. Journal of Econometrics. 1988;37 1:87-114. doi:https://doi.org/10.1016/0304-4076(88)90077-2.
40. Zheng Y, Jiao C, Sun H, Rosli H, Pombo M, Zhang P, et al. iTAK: A Program for Genome-wide Prediction and Classification of Plant Transcription Factors, Transcriptional Regulators, and Protein Kinases. 2016.
41. Moerman T, Aibar Santos S, Bravo Gonzalez-Blas C, Simm J, Moreau Y, Aerts J, et al. GRNBoost2 and Arboreto: efficient and scalable inference of gene regulatory networks. Bioinformatics. 2019;35 12:2159-61. doi:10.1093/bioinformatics/bty916.
42. Csardi G and Nepusz T. The igraph software package for complex network research. InterJournal, Complex Systems. 2006;1695 5:1--9.
43. Molloy M, Donohoe S, E Brzezinski E, Kilby G, Stevenson T, David Baker J, et al. Large-scale evaluation of quantitative reproducibility and proteome coverage using acid cleavable isotope coded affinity tag mass spectrometry for proteomic profiling. 2005.
44. Tamiru M, Undan JR, Takagi H, Abe A, Yoshida K, Undan JQ, et al. A cytochrome P450, OsDSS1, is involved in growth and drought stress responses in rice (Oryza sativa L.). Plant Mol Biol. 2015;88 1-2:85-99. doi:10.1007/s11103-015-0310-5.
45. Wendelboe-Nelson C and Morris PC. Proteins linked to drought tolerance revealed by DIGE analysis of drought resistant and susceptible barley varieties. Proteomics. 2012;12 22:3374-85. doi:10.1002/pmic.201200154.
46. Ali M, Luo DX, Khan A, Haq SU, Gai WX, Zhang HX, et al. Classification and Genome-Wide Analysis of Chitin-Binding Proteins Gene Family in Pepper (Capsicum annuum L.) and

Transcriptional Regulation to Phytophthora capsici, Abiotic Stresses and Hormonal Applications. Int J Mol Sci. 2018;19 8 doi:10.3390/ijms19082216.
47. DroughtDB. http://pgsb.helmholtz-muenchen.de/droughtdb/
48. Schaller G, Street I and Kieber J. Cytokinin and the cell cycle. 2014.
49. Nishiyama R, Watanabe Y, Fujita Y, Le DT, Kojima M, Werner T, et al. Analysis of Cytokinin Mutants and Regulation of Cytokinin Metabolic Genes Reveals Important Regulatory Roles of Cytokinins in Drought, Salt and Abscisic Acid Responses, and Abscisic Acid Biosynthesis. The Plant Cell. 2011;23 6:2169. doi:10.1105/tpc.111.087395.
50. Farber M, Attia Z and Weiss D. Cytokinin activity increases stomatal density and transpiration rate in tomato. 2016.
51. D King C, Rios G, D Green M and R Tephly T. UDP-Glucuronosyltransferases. 2000.
52. Li P, Li Y-J, Zhang F-J, Zhang G-Z, Jiang X-Y, Yu H-M, et al. The Arabidopsis UDP-glycosyltransferases UGT79B2 and UGT79B3, contribute to cold, salt and drought stress tolerance via modulating anthocyanin accumulation. The Plant Journal. 2017;89 1:85-103. doi:10.1111/tpj.13324.
53. Van Huijsduijnen RAMH, Alblas SW, De Rijk RH and Bol JF. Induction by Salicylic Acid of Pathogenesis-related Proteins and Resistance to Alfalfa Mosaic Virus Infection in Various Plant Species. Journal of General Virology. 1986;67 10:2135-43. doi:https://doi.org/10.1099/0022-1317-67-10-2135.
54. Fayez KA and Bazaid SA. Improving drought and salinity tolerance in barley by application of salicylic acid and potassium nitrate. Journal of the Saudi Society of Agricultural Sciences. 2014;13 1:45-55. doi:https://doi.org/10.1016/j.jssas.2013.01.001.
55. Vishwakarma K, Upadhyay N, Kumar N, Yadav G, Singh J, Mishra RK, et al. Abscisic Acid Signaling and Abiotic Stress Tolerance in Plants: A Review on Current Knowledge and Future Prospects. Frontiers in Plant Science. 2017;8 161 doi:10.3389/fpls.2017.00161.
56. Sah SK, Reddy KR and Li J. Abscisic Acid and Abiotic Stress Tolerance in Crop Plants. Front Plant Sci. 2016;7:571. doi:10.3389/fpls.2016.00571.
57. Shi J, Gao H, Wang H, Lafitte HR, Archibald RL, Yang M, et al. ARGOS8 variants generated by CRISPR-Cas9 improve maize grain yield under field drought stress conditions. Plant Biotechnol J. 2017;15 2:207-16. doi:10.1111/pbi.12603.
58. Kim JS, Jung HJ, Lee HJ, Kim KA, Goh C-H, Woo Y, et al. Glycine-rich RNA-binding protein7 affects abiotic stress responses by regulating stomata opening and closing in Arabidopsis thaliana. The Plant Journal. 2008;55 3:455-66. doi:10.1111/j.1365-313X.2008.03518.x.
59. Vítámvás P, Urban M, Škodáček Z, Kosová K, Pitelková I, Vítámvás J, et al. Quantitative analysis of proteome extracted from barley crowns grown under different drought conditions. Frontiers in Plant Science. 2015;6 479 doi:10.3389/fpls.2015.00479.
60. Mierziak J, Wojtasik W, Kostyn K, Czuj T, Szopa J and Kulma A. Crossbreeding of transgenic flax plants overproducing flavonoids and glucosyltransferase results in progeny with improved antifungal and antioxidative properties. Molecular Breeding. 2014;34 4:1917-32. doi:10.1007/s11032-014-0149-5.
61. Apel K and Hirt H. REACTIVE OXYGEN SPECIES: Metabolism, Oxidative Stress, and Signal Transduction. Annual Review of Plant Biology. 2004;55 1:373-99. doi:10.1146/annurev.arplant.55.031903.141701.
62. Kosová K, Vítámvás P, Prášil IT and Renaut J. Plant proteome changes under abiotic stress —

Contribution of proteomics studies to understanding plant stress response. Journal of Proteomics. 2011;74 8:1301-22. doi:https://doi.org/10.1016/j.jprot.2011.02.006.

63. Chmielewska K, Rodziewicz P, Swarcewicz B, Sawikowska A, Krajewski P, Marczak Ł, et al. Analysis of Drought-Induced Proteomic and Metabolomic Changes in Barley (Hordeum vulgare L.) Leaves and Roots Unravels Some Aspects of Biochemical Mechanisms Involved in Drought Tolerance. Frontiers in Plant Science. 2016;7:1108.

64. Ghabooli M, Khatabi B, Ahmadi FS, Sepehri M, Mirzaei M, Amirkhani A, et al. Proteomics study reveals the molecular mechanisms underlying water stress tolerance induced by Piriformospora indica in barley. Journal of Proteomics. 2013;94:289-301. doi:https://doi.org/10.1016/j.jprot.2013.09.017.

65. Bosch M, Mayer CD, Cookson A and Donnison IS. Identification of genes involved in cell wall biogenesis in grasses by differential gene expression profiling of elongating and non-elongating maize internodes. J Exp Bot. 2011;62 10:3545-61. doi:10.1093/jxb/err045.

66. Seo PJ and Park CM. Cuticular wax biosynthesis as a way of inducing drought resistance. Plant Signal Behav. 2011;6 7:1043-5. doi:10.4161/psb.6.7.15606.

67. Hong JK, Choi HW, Hwang IS, Kim DS, Kim NH, Choi DS, et al. Function of a novel GDSL-type pepper lipase gene, CaGLIP1, in disease susceptibility and abiotic stress tolerance. Planta. 2008;227 3:539-58. doi:10.1007/s00425-007-0637-5.

68. Miyakawa T, Hatano K, Miyauchi Y, Suwa Y, Sawano Y and Tanokura M. A secreted protein with plant-specific cysteine-rich motif functions as a mannose-binding lectin that exhibits antifungal activity. Plant Physiol. 2014;166 2:766-78. doi:10.1104/pp.114.242636.

69. Lu L, Dong C, Liu R, Zhou B, Wang C and Shou H. Roles of Soybean Plasma Membrane Intrinsic Protein GmPIP2;9 in Drought Tolerance and Seed Development. Front Plant Sci. 2018;9:530. doi:10.3389/fpls.2018.00530.

70. Sinha AK, Jaggi M, Raghuram B and Tuteja N. Mitogen-activated protein kinase signaling in plants under abiotic stress. Plant Signal Behav. 2011;6 2:196-203. doi:10.4161/psb.6.2.14701.

71. Jonak C, Okresz L, Bogre L and Hirt H. Complexity, cross talk and integration of plant MAP kinase signalling. Curr Opin Plant Biol. 2002;5 5:415-24.

72. Tena G, Asai T, Chiu WL and Sheen J. Plant mitogen-activated protein kinase signaling cascades. Curr Opin Plant Biol. 2001;4 5:392-400.

73. Wu J, Kim SG, Kang KY, Kim JG, Park SR, Gupta R, et al. Overexpression of a Pathogenesis-Related Protein 10 Enhances Biotic and Abiotic Stress Tolerance in Rice. Plant Pathol J. 2016;32 6:552-62. doi:10.5423/PPJ.OA.06.2016.0141.

74. Tripathi P, Rabara R and Rushton P. A systems biology perspective on the role of WRKY transcription factors in drought responses in plants. 2013.

75. Castilhos G, Lazzarotto F, Spagnolo-Fonini L, Bodanese-Zanettini MH and Margis-Pinheiro M. Possible roles of basic helix-loop-helix transcription factors in adaptation to drought. Plant Sci. 2014;223:1-7. doi:10.1016/j.plantsci.2014.02.010.

76. Khatun K, Robin AHK, Park JI, Nath UK, Kim CK, Lim KB, et al. Molecular Characterization and Expression Profiling of Tomato GRF Transcription Factor Family Genes in Response to Abiotic Stresses and Phytohormones. Int J Mol Sci. 2017;18 5 doi:10.3390/ijms18051056.

77. Mihaylova MM and Shaw RJ. The AMPK signalling pathway coordinates cell growth, autophagy and metabolism. Nat Cell Biol. 2011;13 9:1016-23. doi:10.1038/ncb2329.

78. Kulik A, Wawer I, Krzywinska E, Bucholc M and Dobrowolska G. SnRK2 protein kinases--key

regulators of plant response to abiotic stresses. OMICS. 2011;15 12:859-72. doi:10.1089/omi.2011.0091.
79. Wang Y; Sang Z; Xu S; Xu Q; Zeng X; Jabu D; Yuan H (2020): Supporting data for "Comparative Proteomics Analysis of Tibetan Hulless Barley under Osmotic Stress via Data-Independent Acquisition Mass Spectrometry" GigaScience Database. http://dx.doi.org/10.5524/100705.

Figure Legends

Figure 1. Comparison of differentially abundant proteins. The pink arrows indicate the comparison between the treatment and control groups. The yellow arrows indicate the comparison in the consecutive developmental stages over time points.

Figure 2. Downregulated and upregulated proteins in the DQ and XL cultivars between the treatment and control groups. The graph is based on the differential abundance analysis by pairwise comparison, showing the number of proteins that are significantly differentially expressed. The blue bars represent the DQ samples, and the green bars represent the XL samples. Among them, the dark and light colours denote the upregulated and downregulated proteins, respectively. The yellow area in the centre of the bar provides the intersection number of DAPs between XL and DQ. The abbreviations beside the bars are the unique annotated functional entries from XL or DQ, and they are manually selected according to the correlation with the osmotic stress resistance.

Figure 3. MapMan enriched heatmap for DAPs in pairwise comparison. The left map shows the annotation of upregulated proteins, and the right map shows the annotation of downregulated proteins. Row names represent the samples from five time points in the DQ and XL cultivars. Column names are the enriched functional categories in the Mapman database. The legend shows the colour scaling with FDR values. Specifically, the coloured cells are the significantly enriched terms with FDR<=0.01, and the grey colour cells are not.

Figure 4. Downregulated and upregulated proteins in the DQ and XL cultivars compared over five time points. The pink and orange bars correspond to the control and treatment groups of the DQ samples, and the green and blue bars correspond to the control and treatment groups of the DQ XL samples. Of these, the dark and light colours denote upregulated and downregulated proteins, respectively. The Venn diagrams show the overlap of four groups in each comparison. In particular, each group contained both upregulated and downregulated DAPs. The abbreviations beside the circles provide the unique annotated functional entries of XL treatment or DQ treatment, and they are manually selected according to the correlation with the osmotic stress resistance.

Figure 5. Protein abundance changes of six core genes in the plant defence response. The labels in the first row of the top panel are the target gene names, and the labels in the second row are the relevant UniProt accession of *Hordeum vulgare* based on BLAST alignment. The biological replicates from the same sample are represented by three different symbols. The treatment and control groups are illustrated with blue and orange colours, respectively.

Figure 6. Regulated network analysis of osmotic stress-induced genes. The table on the left presents the target gene list used in this analysis. Blue and green nodes in the network correspond to the transcription factors and protein kinases, respectively. The orange nodes are the manually reviewed core genes that are described in Figure 5. The purple nodes represent the identified genes in the XL or DQ cultivars. The size of a node is proportional to its degree. Nodes with higher degrees, which means having more neighbours, will have a stronger capacity to modulate adjacent genes than genes with lower degrees.

Figure 1

Click here to access/download;Figure;Figure 1.pdf

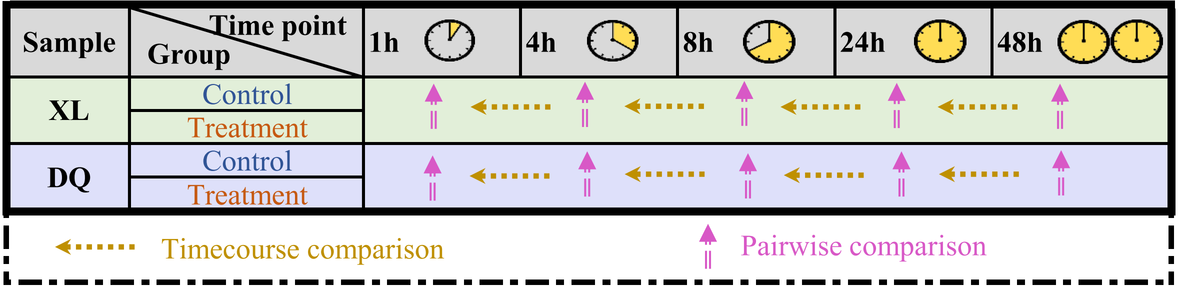

Figure 2

Click here to access/download;Figure;Figure 2.pdf

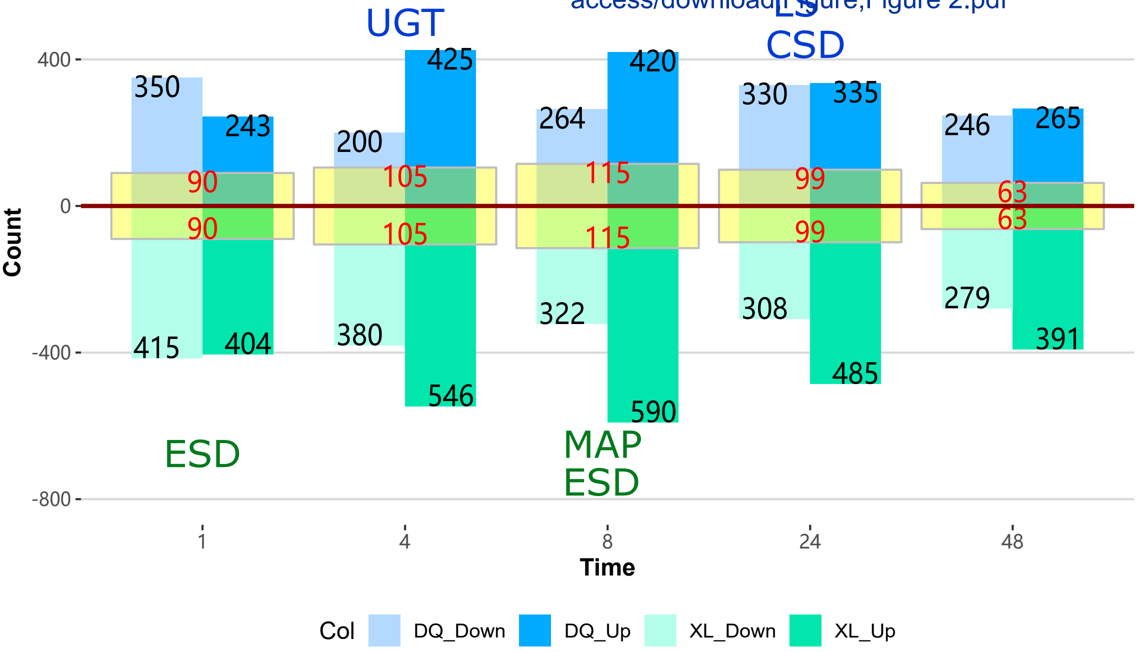

DQ unique DEP	CSD	hormone metabolism.cytokinin.synthesis-degradation
	UGT	misc.UDP glucosyl and glucoronyl transferases
	LS	GO:0010150~BP:leaf senescence
XL unique DEP	MAP	signalling.MAP kinases
	ESD	hormone metabolism.ethylene.synthesis-degradation

Figure 3

Click here to access/download;Figure;Figure 3.pdf

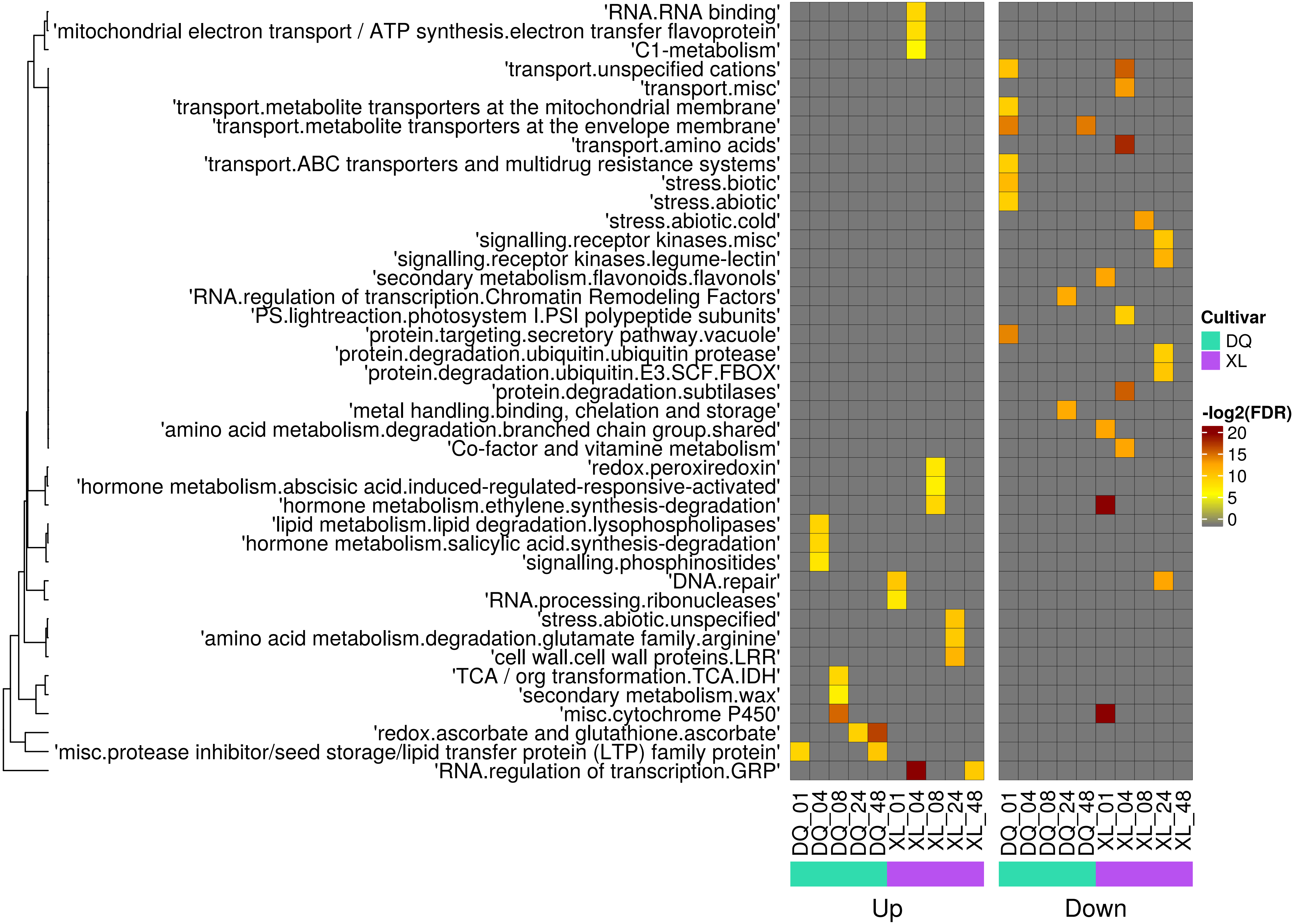

Figure 4

Click here to access/download;Figure;Figure 4.pdf

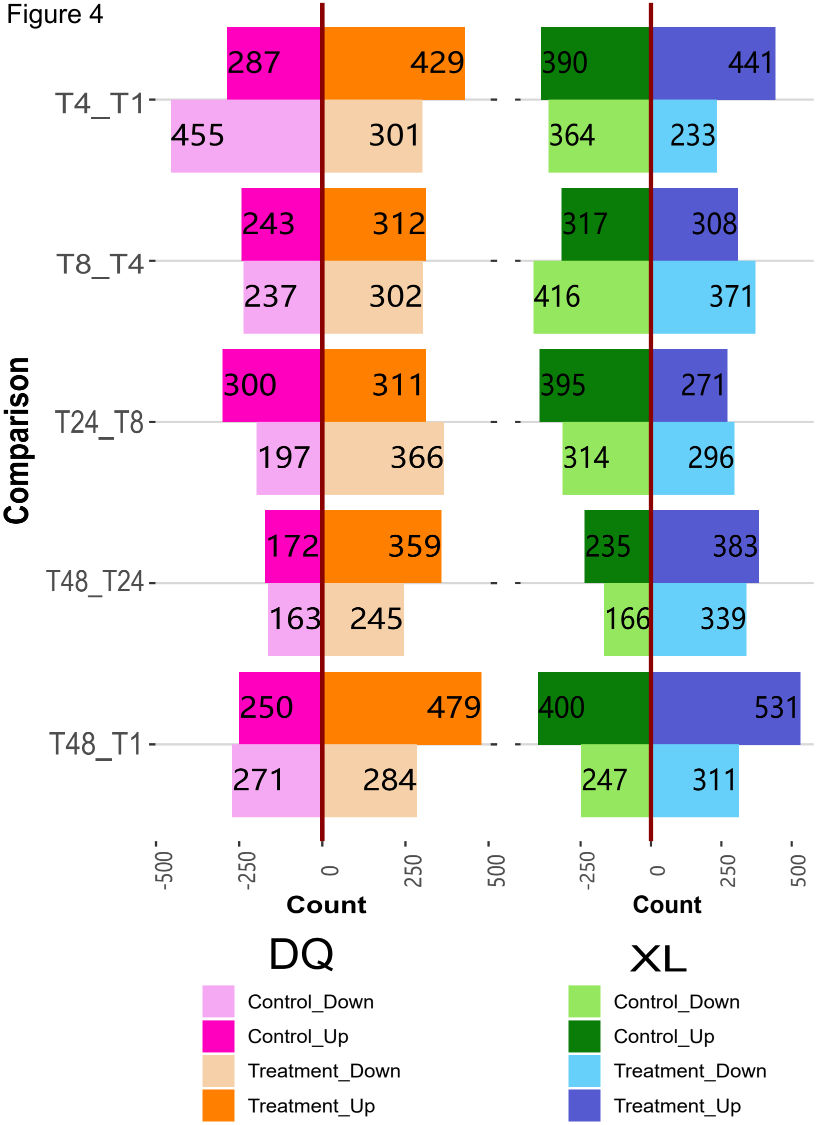

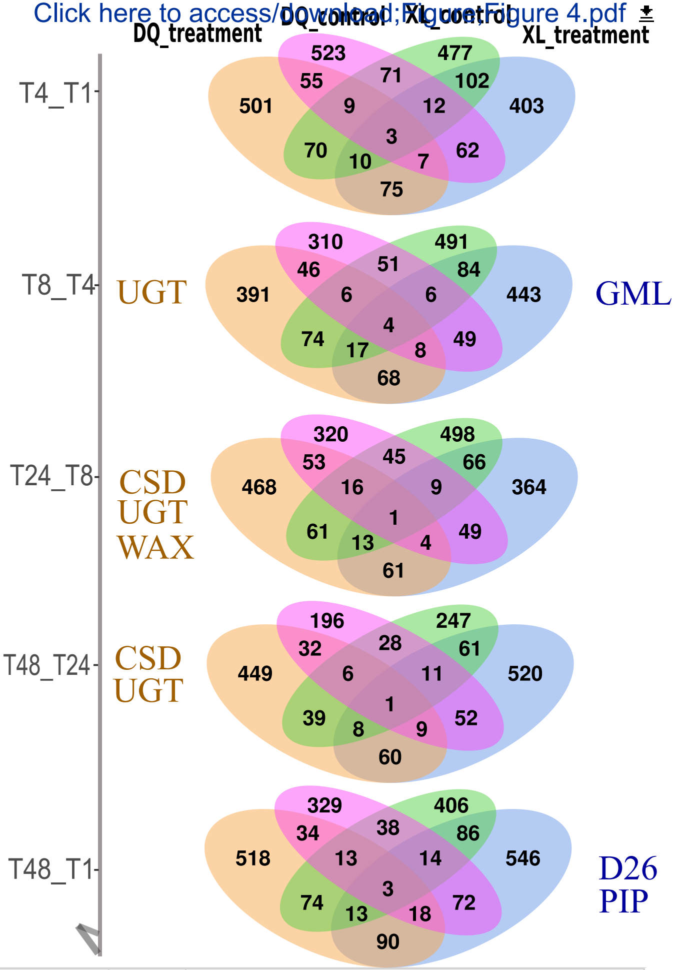

DQ unique DEP	CSD	hormone metabolism.cytokinin.synthesis-degradation
	UGT	misc.UDP glucosyl and glucoronyl transferases
	WAX	secondary metabolism.wax
XL unique DEP	GML	misc.GDSL-motif lipase
	D26	signalling.receptor kinases.DUF 26
	PIP	transport.Major Intrinsic Proteins.PIP

Figure 5

Click here to access/download;Figure;Figure 5.pdf

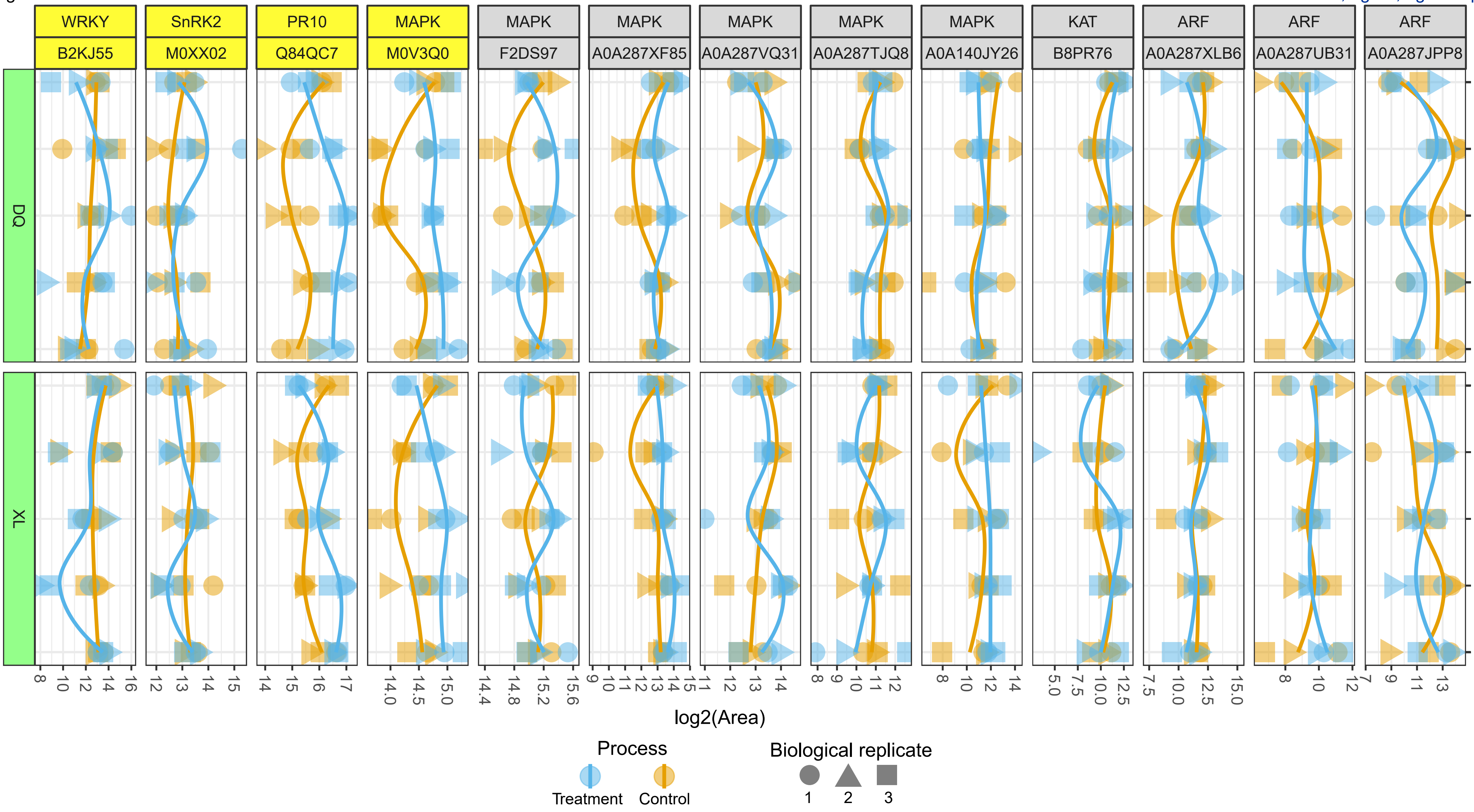

Figure 6

Click here to access/download;Figure;Figure 6.pdf

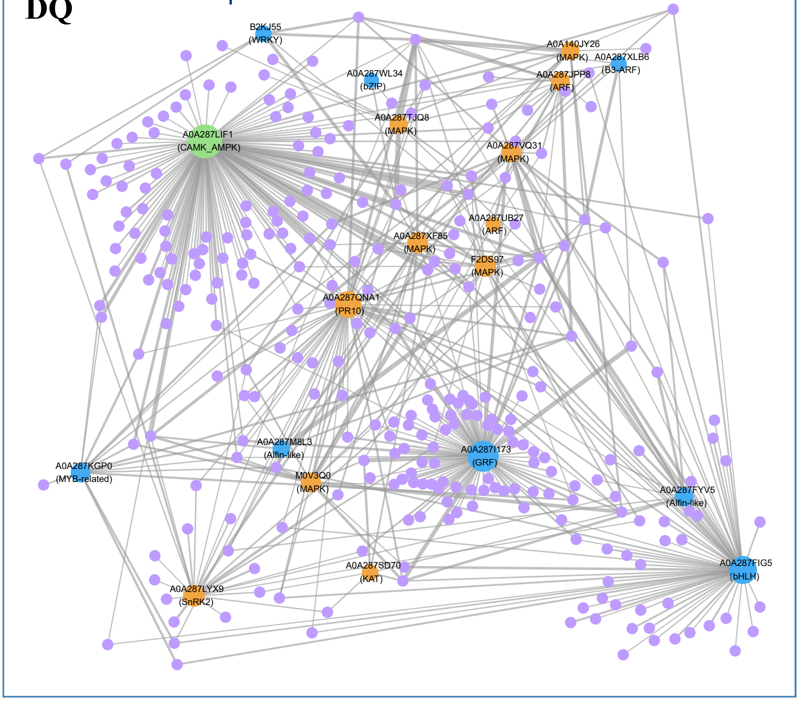

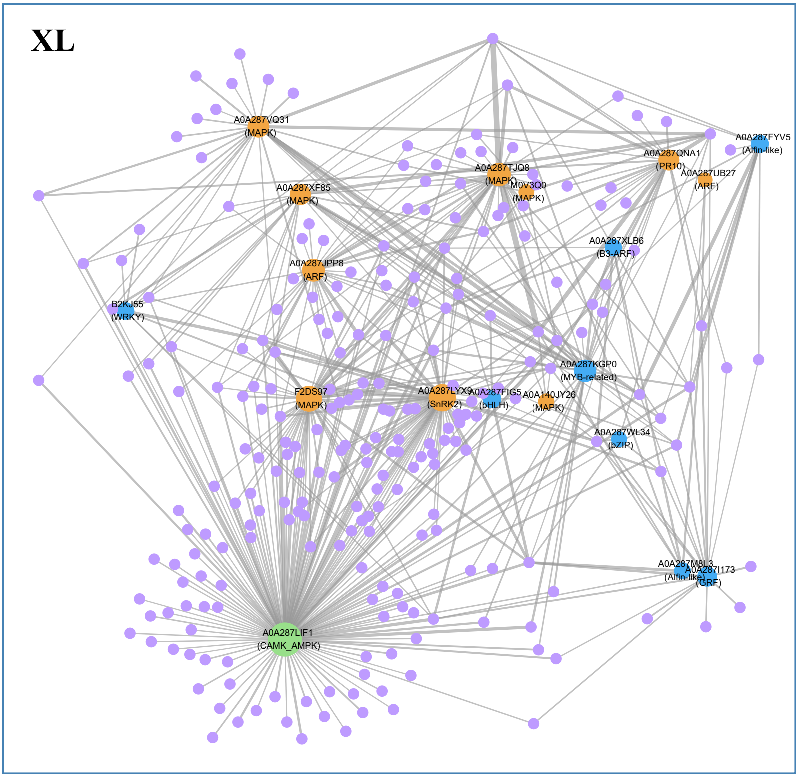

Uniprot ID	Class	Gene family
A0A287M8L3	TF	Alfin-like
A0A287FYV5	TF	Alfin-like
B2KJ55	TF	WRKY
A0A287KGP0	TF	MYB-related
A0A287WL34	TF	bZIP
A0A287I173	TF	GRF
A0A287FIG5	TF	bHLH
A0A287XLB6	TF	B3-ARF
A0A287LIF1	PK	CAMK_AMPK
A0A287JPP8	Core	ARF
A0A287UB27	Core	ARF
A0A287XLB6	Core	ARF
A0A287SD70	Core	KAT
A0A140JY26	Core	MAPK
A0A287TJQ8	Core	MAPK
A0A287VQ31	Core	MAPK
A0A287XF85	Core	MAPK
F2DS97	Core	MAPK
M0V3Q0	Core	MAPK
A0A287LYX9	Core	SnRK2
A0A287QNA1	Core	PR10

Click here to access/download
Supplementary Material
Supplementary figures S1-S5.docx

Click here to access/download
Supplementary Material
Supplementary tables S1-S6.xlsx

Click here to access/download
Supplementary Material
Supplementary tables S7-S8.xlsx
