# Supplementary material for: Comparative proteomics analysis of Tibetan hull-less barley under osmotic stress via data-independent acquisition mass spectrometry
Source: Gigascience. 2020 Mar 3;9(3):giaa019. doi: 10.1093/gigascience/giaa019 (PMC7053489; doi:10.1093/gigascience/giaa019)
Supplement: giaa019_Supplemental_Figures_and_Tables [file giaa019_supplemental_figures_and_tables.zip › Supplementary figures S1-S5.docx]

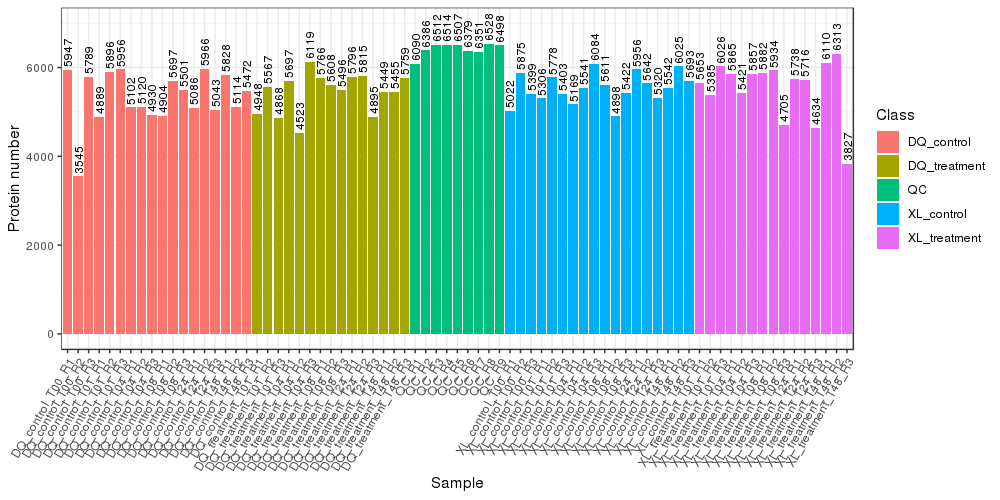


Figure S1. Numbers of proteins detected in each sample.


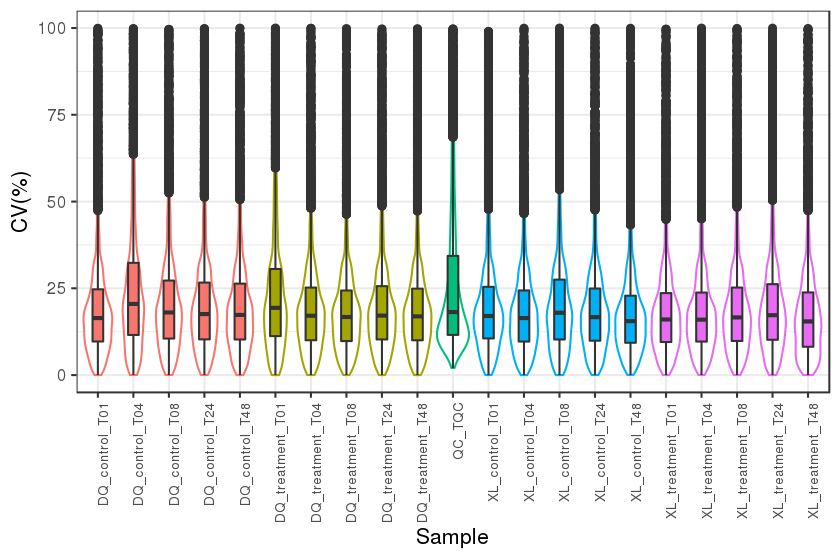


Figure S2a. Distribution of protein abundance variability. The CV value of each protein was calculated by R environment with formula as “sd(biological replicates)/mean(biological replicates) )”.


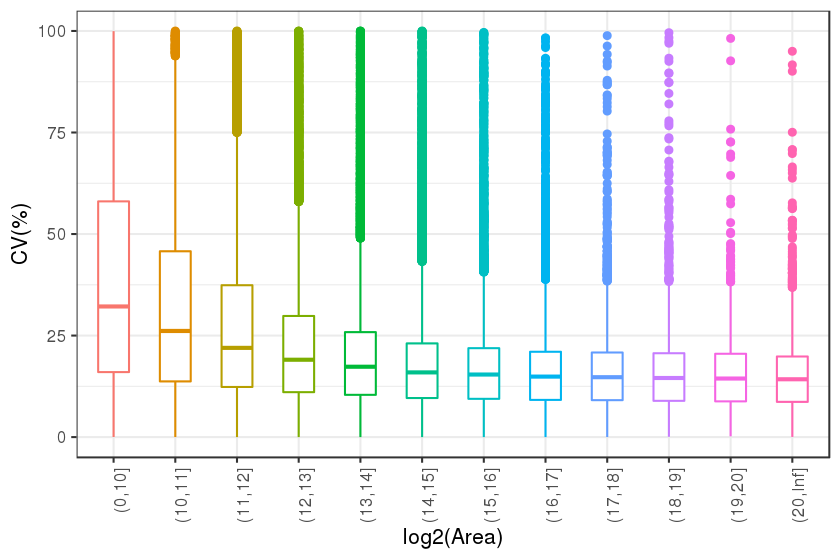


Figure S2b. Relationship between CV and protein abundance (log2 transformed). The CV value drops with increasing protein abundance.


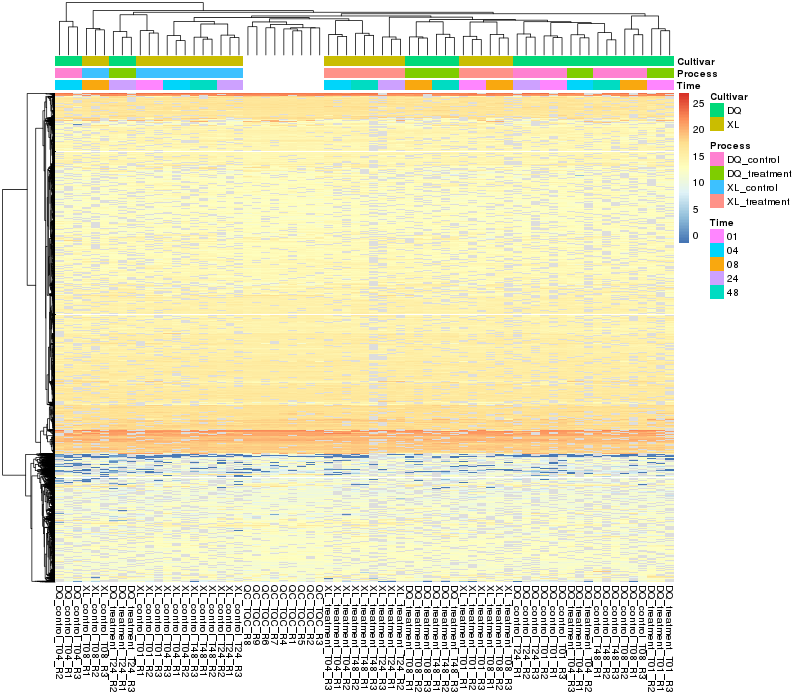


Figure S2c. Heatmap of protein abundances between different samples. The hierarchical clustering is performed using neighbor joining algorithm with a Euclidean distance similarity measurement of the log2 of the protein abundance.


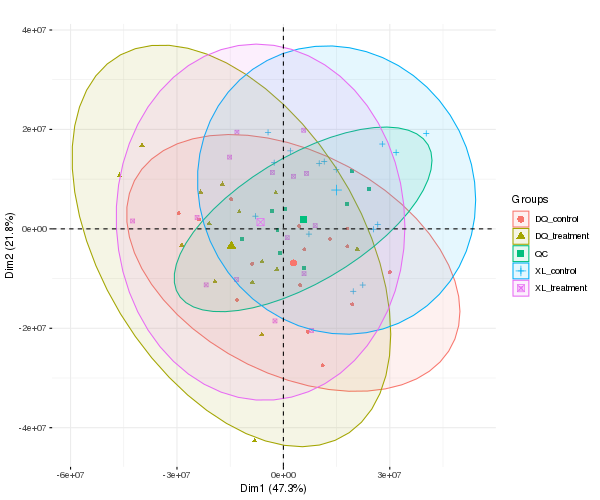


Figure S2d. Principal component analysis (PCA) score plot for proteins in the DQ and XL cultivars between the treatment and control groups. Each point represents a sample.


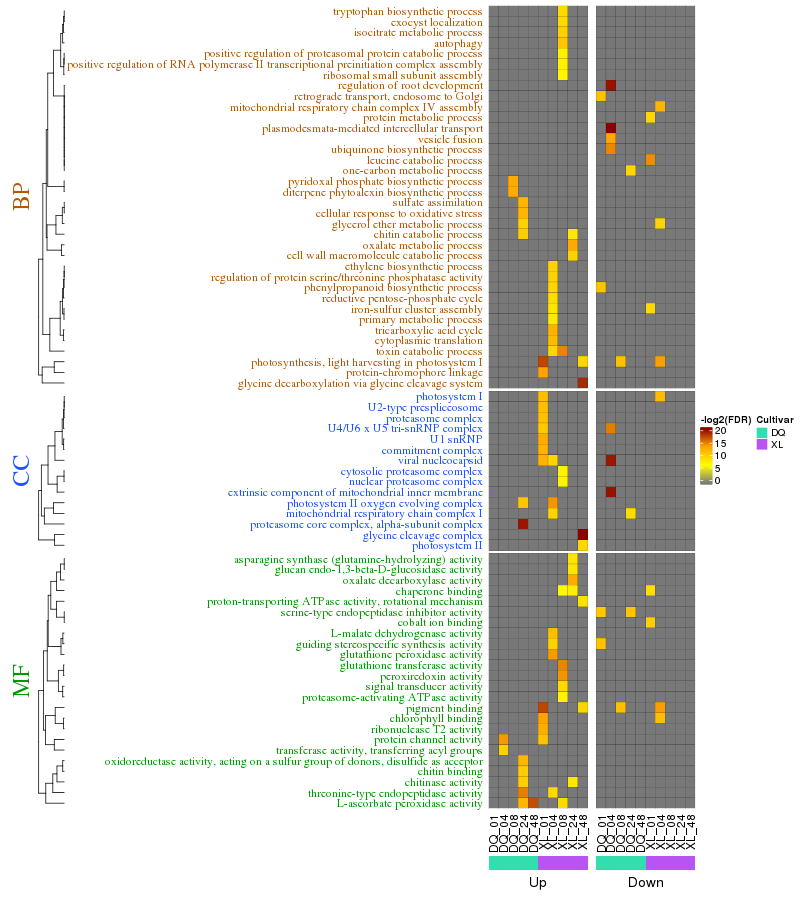


Figure S3. Gene ontology enriched heatmap for DAPs in pairwise comparison. Similar to Figure 3, but with gene ontology instead of MapMan database. The left panel shows the annotation of upregulated proteins and the right panel shows the annotation of downregulated proteins. Row names are the samples from five time points in the DQ and XL cultivars. Column names are the enriched items from three aspects of gene ontology database (biological process: BP; cellular component: CC; and molecular function: MF). The legend shows the colour scaling with FDR values. See Supplementary Table S1 for the entire list of the GO terms


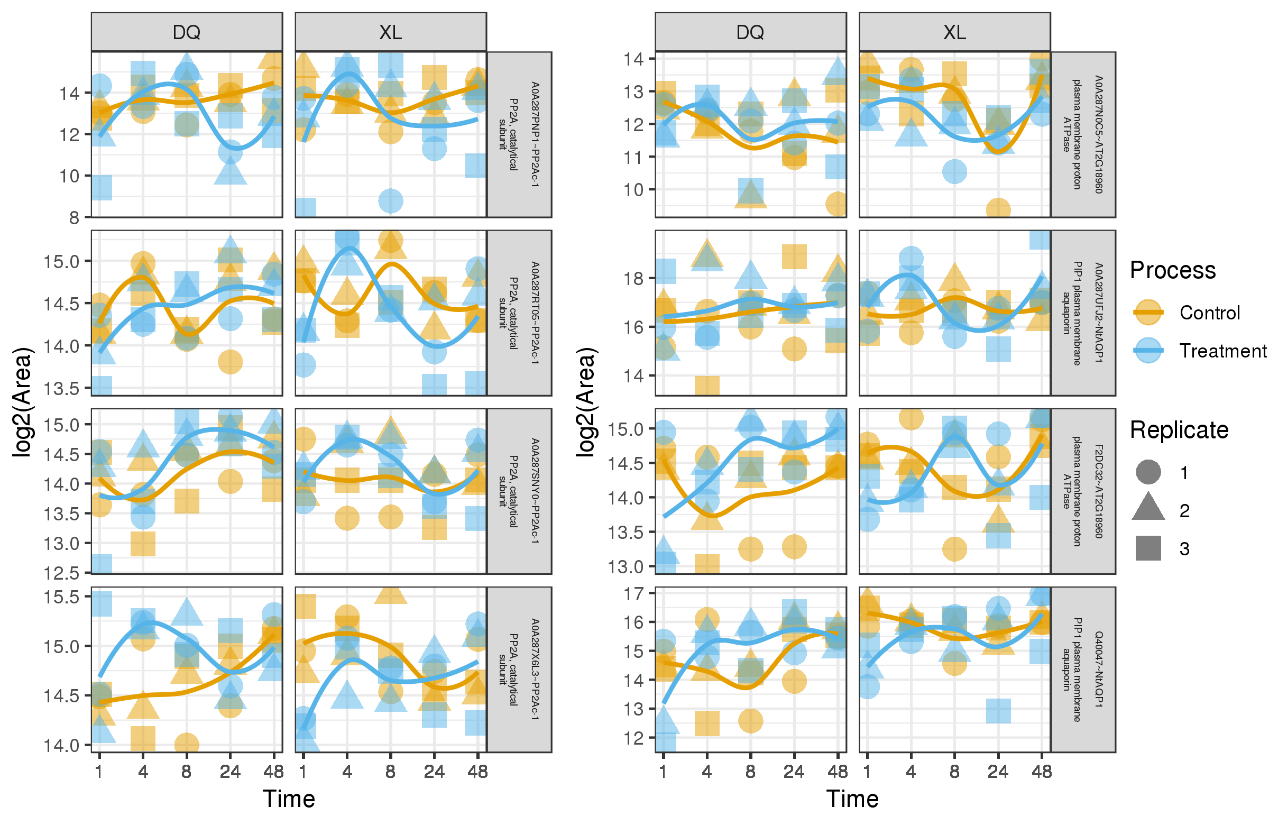


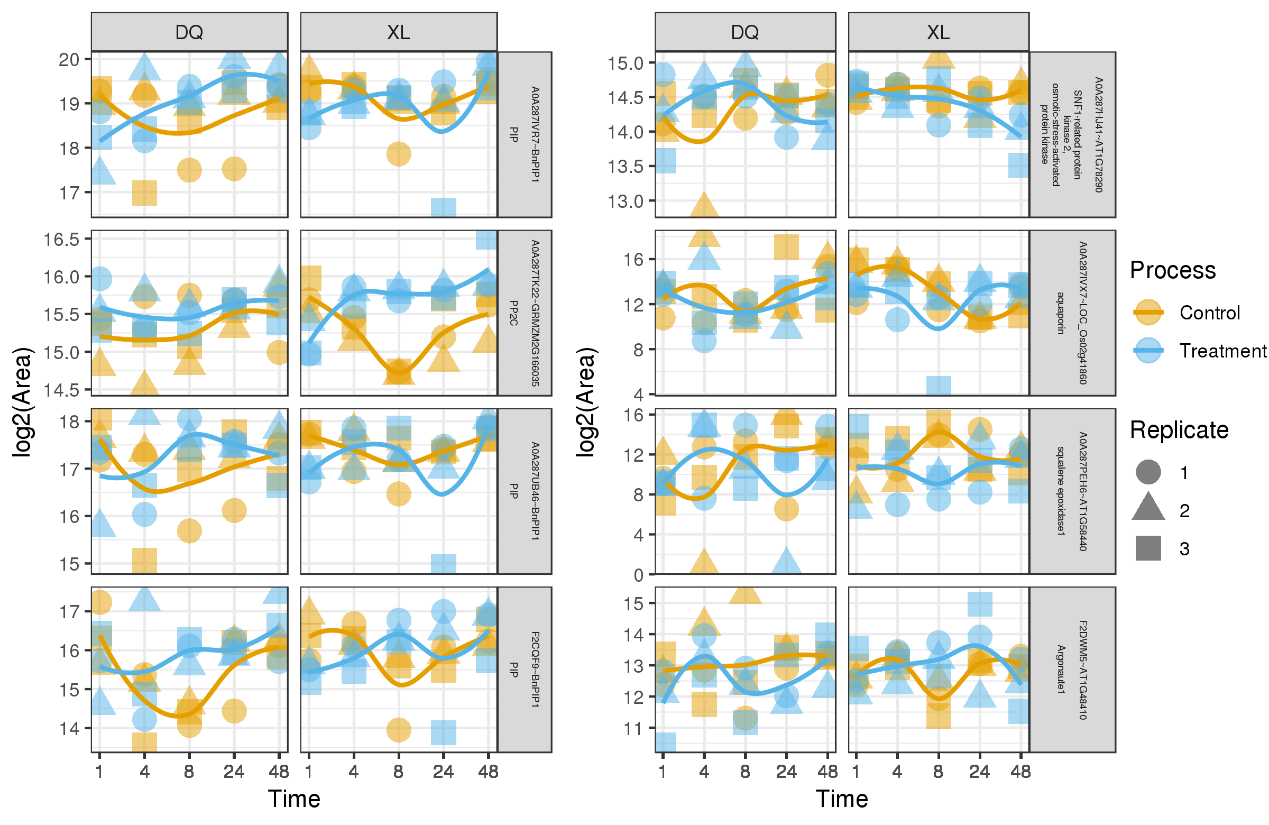


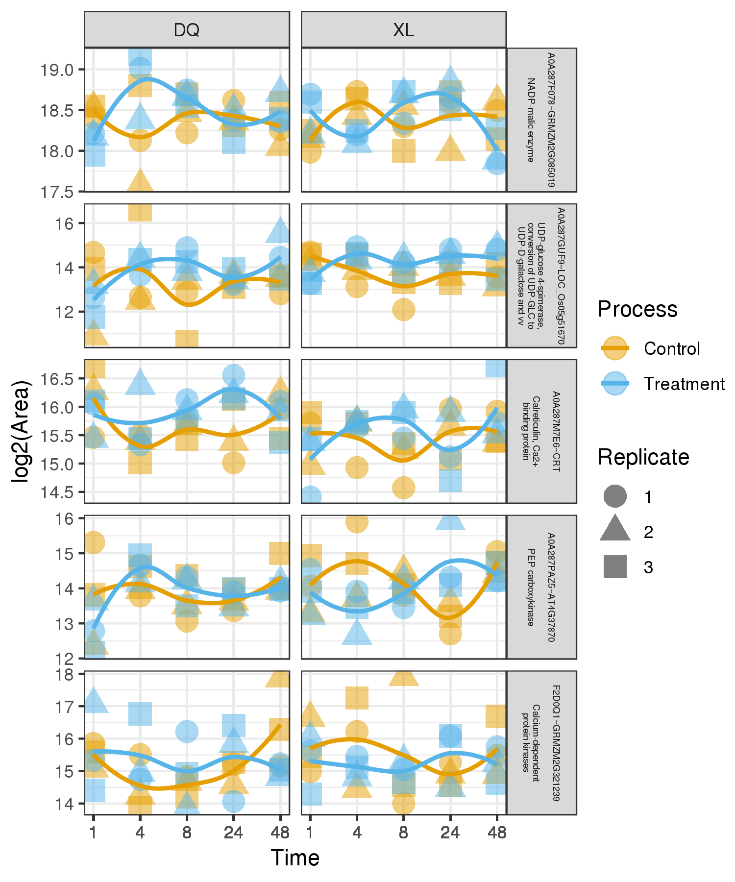


Figure S4. Protein abundance changes of osmotic stress-induced genes from the DroughtDB database. Similar to Figure 5, the labels in the left panel are the description of the related genes. In particular, the identifier in the left of tilde (~) symbol is the UniProt accession of Hordeum vulgare, the identifier in the right of tilde is the gene symbol from DroughtDB, and the description under the tilde is the osmotic stress-related functional annotation.


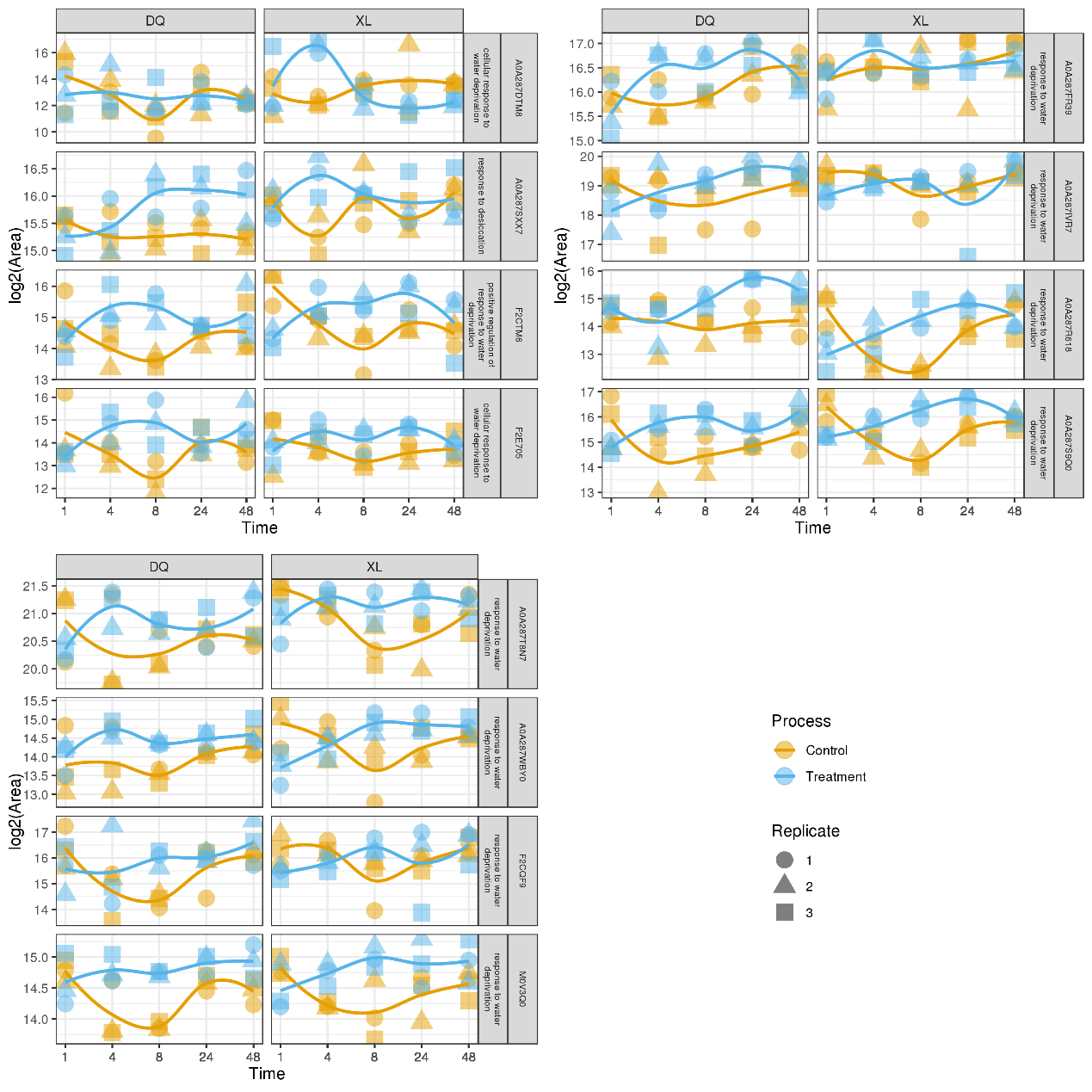


Figure S5. Protein abundance changes of osmotic stress-induced genes from gene ontology database. Similar to Figure 5, the labels in the first left panel are the UniProt accessions of Hordeum vulgare, and the labels in the second left panel are the functional description from gene ontology with BLAST.
