# Supplementary material for: Comparative proteomics analysis of Tibetan hull-less barley under osmotic stress via data-independent acquisition mass spectrometry
Source: Gigascience. 2020 Mar 3;9(3):giaa019. doi: 10.1093/gigascience/giaa019 (PMC7053489; doi:10.1093/gigascience/giaa019)
Supplement: giaa019_Response_to_Reviewer_Comments_Original_Submission [file giaa019_response_to_reviewer_comments_original_submission.pdf]

Author's Response To Reviewer Comments

Close

Dear Editor,

We are very thankful to the reviewers for their constructive comments to help improve our manuscript. We have considered all comments and suggestions and revised the manuscript accordingly.

Point-by-point replies are listed as below.
Reviewer #1:
Terminology:
Osmotic stress vs drought stress: The authors used 21% PEG-6000 treatment for simulation of drought stress. However, I recommend to refer to PEG-6000 treatment as an osmotic stress, not drought stress since drought means a lack of water in soil resulting in decreased soil water content while PEG-6000 treatment can reveal differential effects on plants due to its different nature. Thus, to be precise, I recommend to use the term „osmotic stress" instead of „drought" for PEG-6000 treatment.
Response: Thank you for your valuable suggestion. We have replaced the "drought" with "osmotic stress" in the revised manuscript according to your suggestion.

Differentially abundant proteins vs Differentially expresed proteins: I recommend the authors to use the term „differentially abundant proteins" instead of „differentially expressed proteins" since Proteomics methods determine protein relative abundance which always represents a result of two opposite processes, protein biosynthesis („protein expression") and protein degradation.
Response: Thank you for your suggestion. We have used "Differentially abundant proteins (DAPs)" instead of "Differentially expressed proteins (DEPs)" in the revised manuscript.

Materials and methods:
In Materials and methods, the source of seeds of the two cultivars of Tibetan hulless barley, drought-senstiive DQ and drought-tolerant XL, has to be given. The authors should write from which institution the seeds were obtained.
Response: I'm sorry this was not clearly described in previous version. We have added a sentence in the revised manuscript to clarify the source of seeds as "Specifically, we acquired the DQ cultivar from Institute of Agricultural Research, Tibet Academy of Agricultural and Animal Husbandry Sciences and XL cultivar from The Tibet Autonomous Region Xigaze Agricultural Science Research Institute.".

RT-qPCR analysis: In Figure 5, the authors present their original data on gene expression levels of six core genes in plant defense response. However, no basic information on the methodology of RT-qPCR including the sequences of forward and reverse primers and the housekeeping gene is given in Materials and methods. The authors have to add basic information on RT-qPCR methodology corresponding to the results presented in Figure 5.
Response: I'm sorry for the inaccurate method descriptions in Figure 5 in previous version. There is no RT-qPCR expeirment in our study. We just used the protein abundance from DIA to generate the smooth curves. In the revised manuscript, we have added a sentence to explain this method as "The abundance curve of target gene was depicted with protein abundance. And a loess method implemented in R environment was used to fit the smooth curves by a set of data points.".

In Figure 5 legend, loess method is mentioned for fitting a set of data points with smooth curves; however, no reference on loess method is given in Materials and methods.
Response: Thank you for pointing out this problem. I have added the reference of loess method in the revised manuscript.

Results:
I would recommend the authors to add a graphical abstract Figure 7 or a summarising table providing a summary of the differences in response to osmotic stress between the two Tibetan hulless barley cultivars, DQ and XL, at proteome level, based on the results of the present study.
Response: Thank you for your valuable suggestion. We have added two supplementary tables S7 and S8

to summarize the functional differences of XL and DQ in response to osmotic stress.

Discussion:
I think that the authors should discuss their results in a broader context of other proteomic studies focused on drought or osmotic stress response in barley or wheat such as Ford et al. 2011, Wendelboe-Nelson and Morris 2012, Ashoub et al. 2013, Ghabooli et al. 2013, Vítámvás et al. 2015 Frontiers in Plant Science 479, and others.
Response: Thank you for your valuable suggestion. According to your suggestion, we have extended the discussion with several relevant proteomic studies as paragraph 3-5 of Discussion in the revised manuscript.

Formal comments on the text:
Use SI units for volume, i.e., use „dm3" instead of „l", „cm3" instead of „mL" and „mm3" instead of „µl."
Abstract, line 4: Do not use contracted forms in Scientific text, i.e., write „Thus, it is critical to explore…" (not „Thus, it´s critical to explore…").
Background, page 2, line 4: Remove the word „be" in the sentence „The droughty agricultural areas are estimated to double by the end of the 21st century…"
Background, page 3, line 15: Correct the term „salicylic acid" (not „salicylic").
Materials and methods, page 4, line 22: Modify the word form „centrifuge" to „centrifuged" in the sentence „After centrifuging…, 100 mm3 of ABC (0.05 M NH4HCO3 in water) was added into the filer unit and centrifuged at 14,000 g."
Materials and methods, page 4, line 24: Modify the word form in the words „after centrifuge" to „after centrifugation."
Materials and methods, page 4, line 36: Add a comma both preceding and following the word „finally" in the sentence „…and, finally, 100% buffer A for 15 min."
Materials and methods, page 6, line 18: Correct the typing error in the term „heatmap package" (not „pheatmap package").
Analyses, page 7, line Analyses, page 7, line 7: Modify the heading „Pairwise differential abundance analysis" according to my note on DAPs vs DEPs in terminology.
Figure 5 legend, line 1. Correct the typing error in the word „plant" (not „pant") in the term „plant defense response."
Response: Thank you very much for your valuable suggestions. We have revised the text accordingly based on your suggestions, except the content about pheatmap package. The pheatmap package is an implementation of pretty heatmap that offers more control over dimensions and appearance. We are sorry for the missing reference of this package in previous version. We have added the reference of the R package pheatmap in the revised manuscript.
Moreover, in order to improve the grammar and readability, we have asked the professional language service (American Journal Experts, AJE) to edit the text and to reduce the mistakes in English writing as much as possible. The certificate can be verified on the AJE website using the verification code D90D-1336-F7B6-6D7C-9159.

Reviewer #2: The author very well explained the hypothesis, methods and results in the manuscript. The manuscript may be accepted after some minor revisions. The author has to revise the manuscript thoroughly from English editing expert along with the following revisions:
Response: Thank you for the suggestion. We have asked the professional language service (American Journal Experts, AJE) to edit the text and to reduce the mistakes in English writing as much as possible. The certificate can be verified on the AJE website using the verification code D90D-1336-F7B6-6D7C-9159.

Page 5, Paragraph no. 3, line no. 8: Please clarify the statement 'The role of proteins ……………translational regulations'.
Response: We are sorry for the inaccurate description and thank you for pointing out this issue. We have modified this paragraph as "To our knowledge, no large-scale proteomic research of Tibetan hulless barley was performed under drought stress. Indeed, mRNA expression is not always a good predictor of protein abundance because low correlations between mRNA and protein abundance are often observed [20-22]. Thus, the precise measurement of the proteome is meaningful for understanding underlying biological mechanisms of Tibetan hulless barley under osmotic/ drought stress." in the Introduction of the revised manuscript.

Page 6, Paragraph no. 2, line no. 7: Please clarify the statement 'Time-course……….. respectively'.
Response: We have modified this statement as "Time-course and pairwise comparison analyses of all samples at each time point were conducted with the protein abundance.".

Page 8, Paragraph no. 4, line no. 5: Rewrite the text 'To evaluate ……… samples'.
Response: We have rewritten this sentence as “To evaluate the reproducibility of the LC-MS system during the whole DIA acquisition, the samples and QCs were analyzed following this scenario: one QC injection followed by 10 experimental samples until all were measured.”.

Page 10, Paragraph no. 2, line no. 10: Please specify the statement 'This results …… treatment group'.
Response: Thanks for your suggestion. After careful consideration, we thought this sentence could not provide a meaningful conclusion in that context. So we have decided to delete it in the revised manuscript.

Page 11, Paragraph no. 3, line no. 4: Rewrite the text 'Significantly ………change≥2'.
Response: Thank you for your suggestion. We have rewritten this sentence as “DAPs were selected based on the threshold of protein abundance fold changes ≥ 2 and p-value ≤ 0.05.” in the revised paragraph;

Page 12, Paragraph no. 2, second last line: Explain the statement 'This result showed …. water deprivation '.
Response: Thank you for your comment. We are sorry for the inaccurate statement that may lead to misunderstanding. We have rewritten it as “This phenomenon indicates that water deprivation is a vital pathway for both XL and DQ under osmotic stress.” in the revised manuscript.

Page 15, Caption for the supplementary Figure S2d 'Principal component analysis (PCA) score plot for proteins in wheat XL and DL' the word 'wheat' is mentioned and the work has been carried out on the barley. please explain?
Response: We are sorry for this typing error. We have corrected it as “'Principal component analysis (PCA) score plot for proteins in XL and DL ……”.

We greatly appreciate your interest and encouragement concerning our manuscript. We look forward to your decision.

Sincerely yours,

Dr. Hongjun Yuan
Tibet Academy of Agricultural and Animal Husbandry Sciences, Lhasa 850002, China.
E-mail: yhjxzls@sina.com.

Close
