# Supplementary material for: Comparative proteomics analysis of Tibetan hull-less barley under osmotic stress via data-independent acquisition mass spectrometry
Source: Gigascience. 2020 Mar 3;9(3):giaa019. doi: 10.1093/gigascience/giaa019 (PMC7053489; doi:10.1093/gigascience/giaa019)
Supplement: giaa019_Reviewer_1_Report_Original_Submission — KosovÃi KlÃira -- 10/22/2019 Reviewed [file giaa019_reviewer_1_report_original_submission.pdf]

Reviewer Report

Title: Comparative Proteomics Analysis of Tibetan Hulless Barley under Osmotic Stress via Data-Independent Acquisition Mass Spectrometry

Version: Original Submission **Date:** 10/22/2019

Reviewer name: KosovÃ¡ KlÃ¡ra

Reviewer Comments to Author:

Dear Authors,
Reviewer comments GIGA-D-19-00339
The manuscript entitled â€žComparative proteomics analysis of Tibetan hulless barley under drought stress via data-independent acquisition mass spectrometry" represents an excellent proteomics and bioinformatics study aimed at an identification and functional characterization of proteins difefrentially responding to osmotic stress represented by 21% PEG-6000 treatment on young plants of two Tibetan hulless barley cultivars with different drought tolerance, a tolerant XL and a sensitive DQ cultivar. The manuscript surely deserves publication due to presentation of novel proteomic data followed by their bioinformatic analyses aimed at an investigation of the identified proteins possible biological functions (Figure 3) and biological networks (Figure 6).
However, I have a few important comments how to improve the quality of the present manuscript:
Terminology:
Osmotic stress vs drought stress: The authors used 21% PEG-6000 treatment for simulation of drought stress. However, I recommend to refer to PEG-6000 treatment as an osmotic stress, not drought stress since drought means a lack of water in soil resulting in decreased soil water content while PEG-6000 treatment can reveal differential effects on plants due to its different nature. Thus, to be precise, I recommend to use the term â€žosmotic stress" instead of â€ždrought" for PEG-6000 treatment.
Differentially abundant proteins vs Differentially expresed proteins: I recommend the authors to use the term â€ždifferentially abundant proteins" instead of â€ždifferentially expressed proteins" since Proteomics methods determine protein relative abundance which always represents a result of two opposite processes, protein biosynthesis (â€žprotein expression") and protein degradation.
Materials and methods:
In Materials and methods, the source of seeds of the two cultivars of Tibetan hulless barley, drought-senstiive DQ and drought-tolerant XL, has to be given. The authors should write from which institution the seeds were obtained.
RT-qPCR analysis: In Figure 5, the authors present their original data on gene expression levels of six core genes in plant defense response. However, no basic information on the methodology of RT-qPCR including the sequences of forward and reverse primers and the housekeeping gene is given in Materials and methods. The authors have to add basic information on RT-qPCR methodology corresponding to the results presented in Figure 5.
In Figure 5 legend, loess method is mentioned for fitting a set of data points with smooth curves; however, no reference on loess method is given in Materials and methods.

Results:
I would recommend the authors to add a graphical abstracto r Figure 7 or a summarising table providing a summary of the differences in response to osmotic stress between the two Tibetan hulless barley cultivars, DQ and XL, at proteome level, based on the results of the present study.
Discussion:
I think that the authors should discuss their results in a broader context of other proteomic studies focused on drought or osmotic stress response in barley or wheat such as Ford et al. 2011, Wendelboe-Nelson and Morris 2012, Ashoub et al. 2013, Ghabooli et al. 2013, VÃ-tÃ¡mvÃ¡s et al. 2015 Frontiers in Plant Science 479, and others.
Formal comments on the text:
Use SI units for volume, i.e., use â€ždm3" instead of â€žl", â€žcm3" instead of â€žmL" and â€žmm3" instead of â€žÎ¼l."
Abstract, line 4: Do not use contracted forms in Scientific text, i.e., write â€žThus, it is critical to explore…" (not â€žThus, itÂ´s critical to explore…").
Background, page 2, line 4: Remove the word â€žbe" in the sentence â€žThe droughty agricultural areas are estimated to double by the end of the 21st century…"
Background, page 3, line 15: Correct the term â€žsalicylic acid" (not â€žsalicylic").
Materials and methods, page 4, line 22: Modify the word form â€žcentrifuge" to â€žcentrifuged" in the sentence â€žAfter centrifuging…, 100 mm3 of ABC (0.05 M NH4HCO3 in water) was added into the filer unit and centrifuged at 14,000 g."
Materials and methods, page 4, line 24: Modify the word form in the words â€žafter centrifuge" to â€žafter centrifugation."
Materials and methods, page 4, line 36: Add a comma both preceding and following the word â€žfinally" in the sentence â€ž…and, finally, 100% buffer A for 15 min."
Materials and methods, page 6, line 18: Correct the typing error in the term â€žheatmap package" (not â€žpheatmap package").
Analyses, page 7, line 2: Add â€ža" preceding the words â€žprevious study", i.e., â€ž…and this is in accordance with a previous study…"
Analyses, page 7, line 7: Modify the heading â€žPairwise differential abundance analysis" according to my note on DAPs vs DEPs in terminology.
Figure 5 legend, line 1. Correct the typing error in the word â€žplant" (not â€žpant") in the term â€žplant defense response."
Final recommendation: Minor revision.

Methods

Are the methods appropriate to the aims of the study, are they well described, and are necessary controls included? Choose an item.

Conclusions

Are the conclusions adequately supported by the data shown? Choose an item.

Reporting Standards

Does the manuscript adhere to the journal's guidelines on [minimum standards of reporting?] Choose an item.

Choose an item.

Statistics

Are you able to assess all statistics in the manuscript, including the appropriateness of statistical tests used? Choose an item.

Quality of Written English

Please indicate the quality of language in the manuscript: Choose an item.

Declaration of Competing Interests

Please complete a declaration of competing interests, considering the following questions:

- Have you in the past five years received reimbursements, fees, funding, or salary from an organisation that may in any way gain or lose financially from the publication of this manuscript, either now or in the future?
- Do you hold any stocks or shares in an organisation that may in any way gain or lose financially from the publication of this manuscript, either now or in the future?
- Do you hold or are you currently applying for any patents relating to the content of the manuscript?
- Have you received reimbursements, fees, funding, or salary from an organization that holds or has applied for patents relating to the content of the manuscript?
- Do you have any other financial competing interests?
- Do you have any non-financial competing interests in relation to this paper?

If you can answer no to all of the above, write 'I declare that I have no competing interests' below. If your reply is yes to any, please give details below.

'I declare that I have no competing interests'

I agree to the open peer review policy of the journal. I understand that my name will be included on my report to the authors and, if the manuscript is accepted for publication, my named report including any attachments I upload will be posted on the website along with the authors' responses. I agree for my report to be made available under an Open Access Creative Commons CC-BY license (http://creativecommons.org/licenses/by/4.0/). I understand that any comments which I do not wish to be included in my named report can be included as confidential comments to the editors, which will not be published.

Choose an item.

To further support our reviewers, we have joined with Publons, where you can gain additional credit to further highlight your hard work (see: https://publons.com/journal/530/gigascience). On publication of this paper, your review will be automatically added to Publons, you can then choose whether or not to claim your Publons credit. I understand this statement.

Yes Choose an item.
