# Supplementary material for: Comparative proteomics analysis of Tibetan hull-less barley under osmotic stress via data-independent acquisition mass spectrometry
Source: Gigascience. 2020 Mar 3;9(3):giaa019. doi: 10.1093/gigascience/giaa019 (PMC7053489; doi:10.1093/gigascience/giaa019)
Supplement: giaa019_Reviewer_2_Report_Original_Submission — N.P. Singh -- 11/6/2019 Reviewed [file giaa019_reviewer_2_report_original_submission.pdf]

Reviewer Report

Title: Comparative Proteomics Analysis of Tibetan Hulless Barley under Osmotic Stress via Data-Independent Acquisition Mass Spectrometry

Version: Original Submission **Date:** 11/6/2019

Reviewer name: N.P. Singh

Reviewer Comments to Author:

The author very well explained the hypothesis, methods and results in the manuscript. The manuscript may be accepted after some minor revisions. The author has to revise the manuscript thoroughly from English editing expert along with the following revisions:
Page 5, Paragraph no. 3, line no. 8: Please clarify the statement 'The role of proteins ....translational regulations'.
Page 6, Paragraph no. 2, line no. 7: Please clarify the statement 'Time-course.......... respectively'.
Page 8, Paragraph no. 4, line no. 5: Rewrite the text 'To evaluate .... samples'.
Page 10, Paragraph no. 2, line no. 10: Please specify the statement 'This results ...... treatment group'.
Page 11, Paragraph no. 3, line no. 4: Rewrite the text 'Significantly ....changeâ‰¥2'.
Page 12, Paragraph no. 2, second last line: Explain the statement 'This result showed .... water deprivation '.
Page 15, Caption for the supplementary Figure S2d 'Principal component analysis (PCA) score plot for proteins in wheat XL and DL' the word 'wheat' is mentioned and the work has been carried out on the barley. please explain?

Methods

Are the methods appropriate to the aims of the study, are they well described, and are necessary controls included? Choose an item.

Conclusions

Are the conclusions adequately supported by the data shown? Choose an item.

Reporting Standards

Does the manuscript adhere to the journal's guidelines on minimum standards of reporting? Choose an item.

Choose an item.

Statistics

Are you able to assess all statistics in the manuscript, including the appropriateness of statistical tests used? Choose an item.

Quality of Written English

Please indicate the quality of language in the manuscript: Choose an item.

Declaration of Competing Interests

Please complete a declaration of competing interests, considering the following questions:

- Have you in the past five years received reimbursements, fees, funding, or salary from an organisation that may in any way gain or lose financially from the publication of this manuscript, either now or in the future?
- Do you hold any stocks or shares in an organisation that may in any way gain or lose financially from the publication of this manuscript, either now or in the future?
- Do you hold or are you currently applying for any patents relating to the content of the manuscript?
- Have you received reimbursements, fees, funding, or salary from an organization that holds or has applied for patents relating to the content of the manuscript?
- Do you have any other financial competing interests?
- Do you have any non-financial competing interests in relation to this paper?

If you can answer no to all of the above, write 'I declare that I have no competing interests' below. If your reply is yes to any, please give details below.

I declare that I have no competing interests

I agree to the open peer review policy of the journal. I understand that my name will be included on my report to the authors and, if the manuscript is accepted for publication, my named report including any attachments I upload will be posted on the website along with the authors' responses. I agree for my report to be made available under an Open Access Creative Commons CC-BY license (http://creativecommons.org/licenses/by/4.0/). I understand that any comments which I do not wish to be included in my named report can be included as confidential comments to the editors, which will not be published.

Choose an item.

To further support our reviewers, we have joined with Publons, where you can gain additional credit to further highlight your hard work (see: https://publons.com/journal/530/gigascience). On publication of this paper, your review will be automatically added to Publons, you can then choose whether or not to claim your Publons credit. I understand this statement.

Yes Choose an item.
